# Supplementary material for: Community-centred interventions for improving public mental health among adults from ethnic minority populations in the UK: a scoping review
Source: BMJ Open. 2021 Apr 8;11(4):e041102. doi: 10.1136/bmjopen-2020-041102 (PMC8039264; doi:10.1136/bmjopen-2020-041102)
Supplement: Supplementary data [file bmjopen-2020-041102supp001.pdf]

Appendix 1. Search strategy in all databases

Database Searched	Ethnicity Terms	Outcome Terms (Mental Health)	Geography Terms	Population terms
**Medline**	minority groups.mp. OR Minority Groups/ OR ethnic groups.mp. OR exp Ethnic Groups/ OR ethnic*.mp. OR Race.mp. OR exp Continental Population Groups/ OR racial.mp. OR<br><br>Black.mp. OR exp African Continental Ancestry Group/ OR Black African*.mp OR British African*.mp OR (Black adj1 British).mp. OR Afro$Caribbean*.mp OR Black adj1 caribbean.mp OR (Afr* adj3 Caribbean) OR Black*.mp. OR<br><br>Asian.mp. OR exp Asian Continental Ancestry Group/ OR Indian*.mp. OR exp Pakistan/ OR Pakistan*.mp. OR Bangladesh*.mp. OR exp Bangladesh/ OR Bengali*.mp/ OR Chinese.mp. OR<br><br>Arab*.mp. OR exp Arabs OR	Mental Health.mp. OR exp Mental Health/ OR mental well$being.mp OR exp Stress, Psychological/ OR psychological well$being.mp. OR Resilience, Psychological/ OR resilien*.mp. OR exp Occupational Stress OR stress.mp.	exp United Kingdom/ OR UK.mp OR United Kingdom.mp. OR Great Britain.mp OR Britain.mp OR GB.mp OR England.mp OR Scotland.mp OR Wales.mp OR North* Ireland.mp OR British.mp OR Scottish.mp OR Welsh.mp OR North* Irish.mp	Adult*<br>Wom?n<br>Men<br>people

	Gypsy or "Irish traveller" OR<br><br>(white and black African*).mp. OR (white and black caribbean).mp. OR (white and Asian).mp. OR<br>mixed ethnicit*.mp			
**Embase**	minority groups.mp. OR exp Minority Groups/ OR ethnic groups.mp. OR exp Ethnic Group/ OR ethnic*.mp. OR exp race/ OR "ethnic or racial aspects"/ OR Race.mp. OR racial.mp. OR<br><br>Black*.mp. OR exp Black person/ OR black african*.mp OR British African*.mp OR (Black adj1 British).mp. OR Afro$Caribbean*.mp OR (Black adj1 caribbean).mp OR (Afr* adj3 Caribbean).mp. OR<br><br>Asian.mp. OR exp Asian/ OR exp Asian continental ancestry group/ OR Indian*.mp. OR exp Indian/ OR Pakistan*.mp. OR exp Pakistan/ OR exp Bangladesh/ OR Bangladesh*.mp. OR Bengali*.mp OR exp "Bengali (people)"/ OR Chinese.mp. OR exp Chinese/ OR	Mental Health.mp. OR exp Mental Health/ OR [mental well$being.mp] OR exp psychological well-being/ OR psychological stress.mp. OR exp mental Stress/OR psychGological well$being.mp. OR exp psychological resilience/ OR resilien*.mp OR job stress/ OR life stress/ OR chronic stress/ OR emotional stress/ OR social stress/ OR behavioural stress/ OR stress.mp.	Exp united Kingdom/ OR United Kingdom.mp. OR UK.mp. OR Great Britain.mp OR Britain.mp OR GB.mp OR England.mp OR Scotland.mp OR Wales.mp OR North* Ireland.mp OR British.mp OR Scottish.mp OR Welsh.mp OR North* Irish.mp	Adult*<br>Wom?n<br>Men<br>people

	Arab*.mp. OR exp Arab/ OR<br><br>Gypsy or "Irish traveller" OR<br><br>(white and black African*).mp. OR (white and black Caribbean*).mp. OR (white and Asian*).mp.] OR mixed ethnic*.mp			
**PsychINFO**	minority groups.mp. OR exp Minority Group/ OR ethnic groups.mp. OR exp 'Racial and Ethnic groups'/ OR ethnic*.mp. OR Race.mp. OR racial.mp. OR 'race and ethnic discrimination'/ OR<br><br>exp Blacks/ OR black*.mp. OR exp African Cultural Groups/ or black African*.mp. OR British African*.mp OR (Black adj1 British).mp. OR Afro$Caribbean*.mp OR (Black adj1 caribbean).mp OR (afr* adj3 Caribbean).mp. OR Asian.mp. OR exp Asians/ OR Indian*.mp. OR Pakistan*.mp. OR Bangladesh*.mp. OR Bengali*.mp OR Chinese.mp. OR<br><br>Arab*.mp. OR<br><br>Gypsy or "Irish traveller" OR	Mental Health.mp. OR exp Mental Health/ OR mental well$being.mp OR exp Well Being/ OR exp Psychological Stress/or psychological well$being.mp. OR exp "Resilience (Psychological)"/OR resilien*.mp. OR exp Occupational Stress/ OR stress.mp. OR exp Chronic stress/ OR exp Social Stress/	United Kingdom.mp. OR UK.mp. OR Great Britain.mp OR Britain.mp OR GB.mp OR England.mp OR Scotland.mp OR Wales.mp OR North* Ireland.mp OR British.mp OR Scottish.mp OR Welsh.mp OR North* Irish.mp	Adult*<br>Wom?n<br>Men<br>people

	(white and black African*).mp. OR (white and black Caribbean*).mp. OR (white and Asian*).mp. OR<br><br>mixed ethnicit*.mp			
**SCOPUS**	("minority group*" OR ethnic* OR race OR racial OR african* OR (black W/1 british) OR afro$caribbean* OR (black W/1 caribbean) OR indian OR pakistani OR arab* OR asian OR indian OR bangladesh* OR bengali OR chinese OR "mixed ethnic*" OR "mixed race")	("mental health" OR "mental well?being" OR "psychological well?being" OR "psychological stress" OR "psychological resilien*")	"united kingdom" OR "Great Britain" OR England OR Scotland OR Wales OR (North* Ireland) OR British OR Scottish OR Welsh OR (North* Irish)	Adult*<br>Wom?n<br>Men<br><br>people
**CINAHL**	(minority group*) OR (ethnic* group*) OR ethnic* OR race OR racial OR "black African" OR "African Cultural Groups" OR (british W/1 african) OR (black W/1 british) OR Afro$ Caribbean* OR (black W/1 caribbean) OR (afr* W/3 caribbean) OR asian OR indian OR pakistan* OR bangladesh* OR bengali OR chinese OR arab* OR	(Mental Health) OR (mental well?being) OR (Well?Being) OR (Psychological Stress) OR (psychological well?being) OR exp (psychological resilien*) OR (Chronic stress) OR (Social Stress)	"united kingdom" OR "Great Britain" OR England OR Scotland OR Wales OR (North* Ireland) OR British OR Scottish OR Welsh OR (North* Irish)	Adult*<br>Wom?n<br>Men<br><br>people

4

	“mixed ethnicit*” or Gypsy or “Irish traveller”			
**Cochrane**	"minority group*" OR ethnic* OR race OR racial OR african* OR (black near/1 british) OR afro$caribbean* OR (black near/1 caribbean) OR asian OR indian OR pakistan* OR bangladesh* OR bengali* OR chinese OR arab* OR "mixed ethnicit*" OR gypsy OR “Irish traveller”	(Mental Health) OR (mental well?being) OR (Well?Being) OR (Psychological Stress) OR (psychological well?being) OR exp (psychological resilien*) OR (Chronic stress) OR (Social Stress)	“united kingdom” OR “Great Britain” OR England OR Scotland OR Wales OR (North* Ireland) OR British OR Scottish OR Welsh OR (North* Irish)	Adult* Wom?n Men people
