# Supplementary material for: Community-centred interventions for improving public mental health among adults from ethnic minority populations in the UK: a scoping review
Source: BMJ Open. 2021 Apr 8;11(4):e041102. doi: 10.1136/bmjopen-2020-041102 (PMC8039264; doi:10.1136/bmjopen-2020-041102)
Supplement: Author's manuscript [file bmjopen-2020-041102.draft_revisions.pdf]

BMJ Open

BMJ Open is committed to open peer review. As part of this commitment we make the peer review history of every article we publish publicly available.

When an article is published we post the peer reviewers' comments and the authors' responses online. We also post the versions of the paper that were used during peer review. These are the versions that the peer review comments apply to.

The versions of the paper that follow are the versions that were submitted during the peer review process. They are not the versions of record or the final published versions. They should not be cited or distributed as the published version of this manuscript.

BMJ Open is an open access journal and the full, final, typeset and author-corrected version of record of the manuscript is available on our site with no access controls, subscription charges or pay-per-view fees (http://bmjopen.bmj.com).

If you have any questions on BMJ Open's open peer review process please email info.bmjopen@bmj.com

BMJ Open

Community-centred interventions for improving public mental health among adults from minority ethnic populations in the United Kingdom: a scoping review

Journal:	*BMJ Open*
Manuscript ID	bmjopen-2020-041102
Article Type:	Original research
Date Submitted by the Author:	01-Jun-2020
Complete List of Authors:	Zijlstra, Geiske; Imperial College London School of Public Health, Department of Primary Care and Public Health<br>Baskin, Cleo; Imperial College London School of Public Health, Department of Primary Care and Public Health<br>McGrath, Mike; UCL, Division of Psychiatry<br>Lee, C; University of Cambridge Institute of Public Health, Forvie Site<br>Duncan, Fiona; Durham University, Sport and Exercise Sciences<br>Oliver, Emily; Durham University, Department of Sport and Exercise Sciences<br>Osborn, David; UCL, Division of Psychiatry; Camden and Islington NHS Foundation Trust<br>Dykxhoorn, Jen; UCL, Division of Psychiatry; UCL, Department of Primary Care and Population Health<br>Kaner, Eileen; Newcastle University, Population Health Sciences Institute<br>LaFortune, Louise; University of Cambridge, Institute of Public Health<br>Walters, Kate; University College London, Department of Primary Care and Population Health<br>Kirkbride, James; University College London, Division of Psychiatry<br>Gnani, Shamini; Imperial College London School of Public Health, Department of Primary Care and Public Health
Keywords:	MENTAL HEALTH, PUBLIC HEALTH, Adult psychiatry < PSYCHIATRY

SCHOLARONE™
Manuscripts

I, the Submitting Author has the right to grant and does grant on behalf of all authors of the Work (as defined in the below author licence), an exclusive licence and/or a non-exclusive licence for contributions from authors who are: i) UK Crown employees; ii) where BMJ has agreed a CC-BY licence shall apply, and/or iii) in accordance with the terms applicable for US Federal Government officers or employees acting as part of their official duties; on a worldwide, perpetual, irrevocable, royalty-free basis to BMJ Publishing Group Ltd ("BMJ") its licensees and where the relevant Journal is co-owned by BMJ to the co-owners of the Journal, to publish the Work in this journal and any other BMJ products and to exploit all rights, as set out in our licence.

The Submitting Author accepts and understands that any supply made under these terms is made by BMJ to the Submitting Author unless you are acting as an employee on behalf of your employer or a postgraduate student of an affiliated institution which is paying any applicable article publishing charge ("APC") for Open Access articles. Where the Submitting Author wishes to make the Work available on an Open Access basis (and intends to pay the relevant APC), the terms of reuse of such Open Access shall be governed by a Creative Commons licence – details of these licences and which Creative Commons licence will apply to this Work are set out in our licence referred to above.

Other than as permitted in any relevant BMJ Author's Self Archiving Policies, I confirm this Work has not been accepted for publication elsewhere, is not being considered for publication elsewhere and does not duplicate material already published. I confirm all authors consent to publication of this Work and authorise the granting of this licence.

For peer review only

Community-centred interventions for improving public mental health among adults from minority ethnic populations in the United Kingdom: a scoping review

Zijlstra G[1*], Baskin C[1*], McGrath M [3], Lee C[6], Duncan F[2], Oliver E. J.[2], Osborn D[3,7], Dykxhoorn J[3,4], Kaner E[5], Lafortune L[6], Walters K[4], Kirkbride JB[3], Gnani S[1], The Public Mental Health Programme

*These authors contributed equally to this work.

1 Department of Primary Care and Public Health, School of Public Health, Imperial College London, Reynolds Building, St Dunstan's Road, London, W6 8RP.

2 Department of Sport and Exercise Sciences, Durham University, 42 Old Elvet, Durham, DH1 3HN,

3 Division of Psychiatry, UCL, Maple House, 149 Tottenham Court Road, London, W1T 7NF.

4 Department of Primary Care and Population Health, UCL Royal Free Campus, Rowland Hill Street, London NW3 2PF

5 Population Health Sciences Institute, Baddiley-Clark Building, Newcastle upon Tyne, NE2 4AX.

6 Cambridge Institute of Public Health, Forvie Site, Robinson Way, Cambridge, CB2 0SR.

7 Camden and Islington NHS Foundation Trust, NW1OPE

Corresponding author: Cleo Baskin

Department of Primary Care and Public Health, Imperial College London

Reynolds Building, St Dunstan's Road, London, W6 8RP

Phone: +447366416304

Email: cleo.baskin18@imperial.ac.uk

Word count: 3998

For peer review only

Abstract

Objectives: Undertake a scoping review to determine the effectiveness of community-centred interventions designed to improve the mental health and well-being of adults from Black, Asian and Minority Ethnic (BAME) populations in the United Kingdom.

Methods: We searched six electronic academic databases for studies published between January 1990-September 2019: Medline, Embase, PsychINFO, Scopus, CINAHL and Cochrane. We used the Preferred Reporting Items for Systematic Reviews and Meta-Analyses (PRISMA) extension for Scoping Reviews checklist and Template for Intervention description and replication (TIDieR) guide for intervention description and data extraction. Quality was assessed using Cochrane Risk of Bias tools. Grey literature results were deemed beyond the scope of this review; preliminary searches found numerous (>50) small scale community interventions without available outcomes data.

Results: Of 4,501 studies, seven met the eligibility criteria of UK-based community interventions targeting mental health in BAME adults: four randomised control trials (RCT), one pre- and post-pilot study, one cross-sectional study and one ethnographic study. Interventions included therapy-style sessions, peer-support groups, educational materials, gym access, and a family services programme. Common components included a focus on tackling social isolation, using lay health workers from within the community, signposting, and cultural adaptation. Only three studies reported a statistically significant positive outcome on mental health outcomes and six were appraised as having a high risk of bias. Knowledge gaps emerged around effective interventions for men, some BAME groups, and tackling the wider determinants of mental health.

For peer review only - http://bmjopen.bmj.com/site/about/guidelines.xhtml

Conclusions: There is a paucity of high-quality evidence regarding community-based interventions focussed on improving public mental health among BAME groups. Decision makers need scientific evidence to inform effective approaches to mitigating health disparities. Our next steps are to map promising community activities and interventions that are currently being provided to help identify emerging evidence.

Keywords

Mental health, well-being, Black, Asian and Minority Ethnic groups, minority groups, ethnicity, community-based

Strengths and limitations of this study

- This scoping review addresses the large evidence gap which exists on the effectiveness of community-centred interventions in improving public mental health of UK black and minority ethnic (BAME) populations, whom are disproportionately affected by poor mental health.
- A six-stage scoping review framework, including stakeholder consultation, was applied to summarise the evidence for community-centred interventions.
- We included all community interventions irrespective of setting and type.
- Analysis of review data were limited to studies published in peer reviewed journals and citation tracking of references.
- We used national definitions for BAME categories but acknowledge challenges in viewing ethnic minority groups as homogenous in their mental health needs.

For peer review only

Introduction

Poor mental well-being disproportionately affects people from minority ethnic populations in the UK(1-6). This is thought to be associated with increased exposure to factors such as socio-economic disadvantage(7,8), racism(9), lower uptake of and poorer satisfaction with health care(10), increased stigma associated with mental illness(11), and high rates of chronic disease(12). Further, the long-standing societal disadvantage and discrimination towards these groups has demonstrable consequences on mental health outcomes, for example through overrepresentation in the criminal justice system(13); lower uptake and poorer satisfaction with care(14); and poorer recovery rates and less likelihood to be in employment(15,16).

In the UK, the term Black, Asian, and Minority Ethnic(BAME) is frequently used to refer to populations and groups who are in a racial or ethnic minority, including some white ethnic minorities (e.g. Traveller communities)(17). This term covers a wide range of people with a diverse range of health needs(18,19). Existing evidence suggests significant and longstanding ethnic inequalities in mental health outcomes(20-24), as emphasised by the Race Equality Foundation's 2020 report(16) and differential and complex risks for poor mental health; certain ethnicities being at higher risk than others. For example, the risk of psychosis among the Black Caribbean population is nearly seven times higher than the White British population(25), but not for Asian groups(26).

Strengthening public mental health has potential to effectively and sustainably reduce social and racial inequalities in mental health outcomes(26,27). In pursuit of this, many UK initiatives have been implemented, such as the 2005 'Delivering Race Equality in

For peer review only

Mental Healthcare Action Plan'(28), nevertheless, they have failed to successfully narrow the observed mental health gap(29,30).

Community-centred interventions (i.e., those that take place in a community setting, or are delivered by the community and/or voluntary sector) are increasingly recognised to have potential to influence factors that protect and promote mental health and wellbeing, such as social connectedness, access to safe and affordable housing, and power in local decision making(31,32). Although the National Health Service(NHS) England in 2017 introduced local financial incentives to improve mental health outcomes for BAME groups(19), local decision making has been hindered by the lack of evidence. The aim of this scoping review was to summarise the evidence for community-centred interventions focussed on improving the public mental health of BAME groups in the UK.

For peer review only

Methods

We undertook a scoping review, an approach for summarizing evidence and identifying knowledge gaps in unclear and emerging fields(33,34), using Arksey and O'Malley's six-stage methodological framework(35). The optional sixth stage of stakeholder consultation was undertaken by involving stakeholder and peer researchers as part of a wider research programme(36). The public were not involved in the design or conduct our research. Scoping reviews were not eligible for prospective registration with the international prospective register for systematic reviews (PROSPERO) at the time of conducting the review(37).

I) Study identification

A systematic search of six electronic databases between January 1990 and September 2019 was conducted: Medline, Embase, PsycINFO, Scopus, CINAHL and Cochrane. The search strategy (Appendix 1) was created with the support of a medical librarian and built around four concepts: ethnicity, age range, geography and mental health outcomes. Intervention terms were not included in order to capture all relevant studies.

We excluded evidence from the grey literature. An initial, rapid search of material that included local government, relevant third sector and NHS websites identified numerous activities and possible interventions (over 50 in a small geographical area), but the vast majority had little or no publicly available outcomes data. Synthesis of this data would have required a different methodological approach and beyond the scope of this review.

For peer review only

Eligibility criteria

Table 1 outlines the inclusion and exclusion criteria used to determine study eligibility. To reflect the intent to inform UK policy and practice and to be representative of the current classification of ethnic groups, only UK studies published from 1990 onwards were included. Systematic data collection on ethnic classification in the UK began with the 1991 Census(38).

We included studies where participants were working aged adults; we adapted the UK definition of working aged adults to be 18 to 64 years who were either well or had a common mental disorder, such as anxiety and depression (excluding those aged 16-18 years). Common mental disorders were included to reflect the high prevalence of these conditions, 1 in 6 adults in England(39) and often go undiagnosed, and so are more amenable to interventions focused on prevention and the promotion of positive mental health(40). Studies focusing on people with severe mental illness (including suicide and psychotic disorders), or people affected by young onset dementia, were not included due to the need for specialist mental health treatment and tailored support. Studies that targeted new migrants and refugee groups were also excluded due to the specific and complex mental health needs of this population, such as the high prevalence of post-traumatic stress disorder(41,42). Studies were included if migrants made up part of the participants, but not if the study was designed specifically for them.

Only community-centred interventions were eligible for inclusion. We defined community-centred broadly to include all interventions that were not clinical in nature,

For peer review only - http://bmjopen.bmj.com/site/about/guidelines.xhtml

that is, did not involve the treatment of patients by health professionals, for example, cognitive behavioural therapy and drug therapy(43,44). Therapy-style sessions that were delivered by lay health workers were included. There is a wide range of overlapping concepts in community- based and community- centred terms, and these were captured by our broad search strategy.

Table 1. Eligibility criteria

Inclusion criteria	Exclusion criteria
Adults aged 18 – 64 years	Children aged 0 -17 or adults aged over 64 years
Individuals from Asian/ Asian British, Black/ African/ Caribbean/ Black British, Mixed/Multiple ethnic groups and Arab ethnic groups	All White British ethnicities other than Gypsy or Irish Traveller, refugees and asylum seekers
Adults with no known mental disorder or diagnosed with a common mental disorder, such as anxiety and depression	Adults with severe mental illness and dementia
Community-centred interventions, i.e. take place in a community setting, or in a health setting but delivered by the community and/or voluntary sector	Clinical interventions that involved treatment by a clinician in any setting
Study measures any domain or aspect of mental health and well-being	No outcome measures for mental health or well-being
All studies based on primary research,	Abstracts, posters, books or chapters, editorials and letters
Study conducted in the UK	Study conducted outside of the UK
Published from 1990 onwards	Published before 1990
Full-text research article available	No full text research article available

The UK Office of National Statistics 2011 Census ethnicity terms were used (Table 2)(45). Ethnic groups were deemed a minority if they fell under ‘Mixed’, ‘Asian’, ‘Black’ and ‘Other’ categories(17). All ethnic groups under the ‘White’ category were excluded apart from the ‘Gypsy or Irish traveller’ group, which was included because of their

For peer review only - http://bmjopen.bmj.com/site/about/guidelines.xhtml

significant social and economic disadvantage and health inequalities compared to any other 'White' category(16).

Table 2. Ethnicity as Classified by the Office for National Statistics(47)

**White**	**English / Welsh / Scottish / Northern Irish / British**
	Irish
	Gypsy or Irish Traveller
	Any other White background
**Mixed / Multiple ethnic groups**	White and Black Caribbean
	White and Black African
	White and Asian
	Any other Mixed / Multiple ethnic background
**Asian / Asian British**	Indian
	Pakistani
	Bangladeshi
	Chinese
	Any other Asian background
**Black / African / Caribbean / Black British**	African
	Caribbean
	Any other Black / African / Caribbean background
**Other ethnic group**	Arab
	Any other ethnic group

II) Study selection

Two independent reviewers (GZ,CB) screened titles and abstracts, and full text reviews of eligible articles. All conflicts were resolved by a third reviewer (SG); this was needed for 32% (n=14) of full text articles. Full texts that were not freely available were accessed via the British Library. Additional articles were identified by examining references and forward-backward citation searching (Figure 1). Articles considered eligible for inclusion were read in full by GZ,CB and SG. Covidence systematic review software(46) was used to remove duplicates and screen search results.

III) Charting of data

For peer review only

Data were extracted by two independent reviewers (GZ,CB). A data extraction framework was developed to capture key study characteristics: author; year of publication; setting; study design; population demographics; sample size; theory of change; and outcomes and results. Intervention details were extracted using the Template for Intervention Description and Replication(TIDieR) guide: rationale; materials; procedures; provider; methods; location; timing; tailoring; modifications; and assessment of intervention adherence(47).

IV) Collation and summary of results

Quality appraisal was performed by two reviewers(GZ,CB) using the Cochrane tool for risk of bias for RCTs(48). Qualitative studies were appraised using the Cochrane Qualitative and Implementation Methods Group guidance and the Mixed-Method Appraisal Tool(MMAT) for mixed methods studies(49,50). A narrative data synthesis was conducted including thematic textual descriptions. Review findings were reported using The Preferred Reporting Items for Systematic Reviews and Meta-Analyses(PRISMA) extension for Scoping Reviews checklist(51).

For peer review only

Results

We identified a total of 4,483 non-duplicate article titles and an additional 18 titles from forward-backward citation searching (Figure 1). Forty-five studies were suitable for full-text screening. Seven studies were deemed eligible for inclusion and data extraction: four RCTs (including one pilot); one pre- and post-intervention pilot study; one cross sectional; and one ethnographic study (Table 3).

The interventions were highly heterogenous, including therapy-style sessions delivered by lay health workers(52,53), peer-support groups(54,55), an educational leaflet(56), free gym access(57), and a family services programme(58). All were conducted in England and the most frequently targeted BAME population groups were of Indian and Pakistani heritage. Five studies specifically targeted participants with anxiety or depression.

Interventions were either single or multiple sessions taking place over a minimum of 10-weeks to eight months (it is unclear whether elements of the family services programme went on for longer). Follow-up ranged from two to nine months. Most interventions aimed to improve mental health by expanding social networks and facilitating peer-to-peer support(52,54,55,58). Three studies aimed to increase access to services by facilitating linkages to complementary social care and mental health services or signposting (52,53,58). Qualitative data commonly found a reduction in social isolation and stress, and improved mood and self-confidence(53,55,57,58). One RCT reported significant improvement in social functioning(54) and two had a statistically significant positive impact on depression outcomes(55,56). However, all but one study(54) were appraised as having a high risk

For peer review only - http://bmjopen.bmj.com/site/about/guidelines.xhtml

of bias. Common limitations of the studies included small sample sizes (n=9-123), short follow-up periods (less than nine months), and participant recruitment.

For peer review only

Table 3. Summary of community-based intervention studies targeted at BAME groups to improve mental health and well-being (N=7)

Author; year	*Study design*	*Participant characteristics*	*Theory of Change*	*Intervention description*	*Results*
Jacob et al; 2002	RCT	Asian women with depression	Educate patients on depression to positively affect their perspective and outcomes of depression	Participants (n=70) received an educational leaflet about depression or usual care	**GHQ-12**: recovery from common mental disorder (defined as ≤ 2): OR 3.4 (95% CI: 1.01 to 11.5) at 2 months
Gater et al; 2010	RCT	British Pakistani women with depression	Improve mental health through reducing social isolation	Participants (n=123) received a social group intervention, antidepressants, or both	**HRSD** (depression): no significant effects<br>**Social functioning**: Significant effect at 3 months for social intervention vs. antidepressants, 6.1 (95% CI: 1.4 to 10.8), p= 0.02, and combined group vs. antidepressants, 5.9 (95% CI: 1.5 to 10.2), p= 0.017. No significant effect at 9 months<br>**Satisfaction**: Significant effects at 3 and 9 months for social vs. antidepressants, 4.9 (95% CI: 2.9 to 7.0), p=0.002; 4.7 (95% CI: 2.6 to 6.7), 0.002 and combined v. antidepressants, 4.3 (95% CI: 2.6 to 6.0), p=0.001; 4.0 (95% CI: 1.8 to 6.2), p=0.005
Afuwape et al; 2010	RCT	Black adults with anxiety and/or depression	Improve psychosocial functioning and feelings of hope through a culturally acceptable care package	Participants (n=40) received individual therapy and group sessions on advice on services, health education and mentoring	**GHQ-29**: Significant effect at 3 months: adjusted mean difference 7.76 (95% CI: 0.86 to 14.65), p = 0.03<br>**GAF**: no significant effect<br>**SF-36**: mental health components had significant effect, −11.93 (95% CI: −21.99 to −1.88), p=0.02.<br>No effect on physical health. No significant differences in cost
Lovell et al; 2014	Pilot RCT	South Asian and Somali women with moderate depression and/or anxiety	Improve treatment of depression and anxiety using social and community interventions	Participants (n=20) received individual or group therapy-style sessions, or usual care. Activities focussed on health and wellbeing; sign posting to service and education opportunities	**CORE-OM:** no effect<br>Small non-significant improvements in depression (PHQ9), health related quality of life (EQ5D) and functioning (WSAS) at 5 months

For peer review only - http://bmjopen.bmj.com/site/about/guidelines.xhtml

Chaudhry et al; 2009	Observational: Pre- and post-intervention	British Pakistani women with depression	Informal social support and mental and physical health education to reduce depression	Participants (n=9) received 10 weekly group sessions in various locations: psycho education, personal grooming, exercise and yoga	**SRQ**: reduction in depression scores pre- 15 (SD = 3.08) to post - 11.7 (SD = 5.95), p = 0.039.<br>SCAN interviews post-intervention diagnosed 2 participants as no longer depressed
Rabiee et al; 2015	Cross-sectional; mixed methods	Deprived constituency in Birmingham	Regular exercise can help improve mood, self-esteem, confidence and quality of life	Gym for Free pilot project providing adults free access to leisure centres	Increased energy levels, confidence, mental well-being, reduction in stress and anxiety improved stress relief, and anger management
Gray; 2003	Ethnographic investigation	BAME families in Tower Hamlets	Reducing social isolation and poverty by improving parenting skills, self-esteem and meeting a welfare advisor	Personalised family support workers; trained volunteers of similar cultural backgrounds	Support workers with shared ethnic and cultural sensitivities developed close relationships over 8 months<br>Reduction in social isolation, bullying and racism<br>Increased advocacy with other professionals and access to relevant services

Abbreviations: GHQ-12, General Health Questionnaire-12; HRSD, Hamilton Rating Scale for Depression; GHQ-29, General Health Questionnaire 29; GAF, Global assessment of Functioning; SF-36, Short form survey 36; CORE-OM, Clinical Outcomes in Routine evaluation- outcome measure scale; SRQ, Self-Reporting Questionnaire.

For peer review only

Outcome measures

Mental health outcomes were predominantly measured with standardised screening instruments for common mental disorders and social functioning (Table 4). Qualitative data were collected mainly through focus groups and semi-structured interviews(53,54,57). Only one study reported on costs, which were calculated by measuring the difference in use of health and social services between the intervention and control group(52).

Table 4. Reported quantitative and qualitative outcomes measures across included studies (n = 7)

Author; year	*Quantitative mental health and well-being outcome measures*	*Qualitative outcomes*
Jacob et al; 2002	• GHQ-12: General Health Questionnaire 12	Short Explanatory Model Interview (SEMI): self-reported perspective on depression
Gater et al; 2010	• HRSD: Hamilton Rating Scale for Depression<br>• Verona Service Satisfaction Scale (measuring social functioning and satisfaction)	Feedback forms and focus groups
Afuwape et al; 2010	• GHQ-28: General Health Questionnaire 28<br>• GAF: Global assessment of Functioning<br>• SF-36: Short form survey (measuring quality of life)<br>• LEDS: Life Events and Difficulties Schedule	None
Lovell et al; 2014	• PHQ-9: Patient Health Questionnaire (measuring depression<br>• COREOM: Clinical outcomes in routine evaluation – outcome measure scale (measuring global distress)<br>• GAD-7: Generalised Anxiety Disorder Assessment<br>• WSAS: Work and Social Adjustment Scale<br>• EQ-5D (measuring health related quality of life)	Semi-structured interviews regarding the acceptability of the intervention
Chaudhry et al; 2009	• SRQ: Urdu versions of the Self Reporting<br>• Questionnaire (screening for mental disorders)<br>• SCAN: The Schedule for Clinical Assessment in Neuropsychiatry	Anecdotal feedback collected by facilitators
Rabiee et al; 2015	• Self-completed questionnaire evaluating experience in accessing services and perceived changes in health and well-being	Focus groups analysed using established frameworks and guidelines (59, 60)
Gray; 2003	None	Family case records and interviews analysed using established frameworks (61,62)

For peer review only - http://bmjopen.bmj.com/site/about/guidelines.xhtml

Addressing social isolation and loneliness

Chaudhry et al.(55) conducted a mixed methods pre- and post-intervention pilot study(n=9) on the efficacy and cultural acceptability of a social group for British Pakistani women with depression. Women diagnosed with depression using the Schedule for Clinical Assessment in Neuropsychiatry (SCAN) were recruited from an ongoing population-based study. Participants attended at least six of ten weekly meetings at a local Pakistani community centre where they engaged in self-selected activities including personal grooming, exercise, yoga, and visits to museums and local shopping malls. They also received a session on psychoeducation. Women were accompanied by female Urdu speakers to and from the community centre to avoid objection from family and community of them being alone; this was reported as essential to the intervention's success.

A significant reduction in depressive symptoms was found at the end of the 10 sessions (SRQ scores pre-intervention:15,SD=3.08; post-intervention:11.7, SD= 5.95, p=0.039) and three women reported a reduction in suicidal ideas. These positive outcomes suggest that a reduction in depressive symptoms is partly attributed to reducing social isolation by bringing the women together. However, this assumption is made from participant's anecdotal feedback. Heterogeneity in the content and attendance of each session makes it difficult to assess which component of the intervention was responsible for improved outcomes and how many sessions were necessary to have an effect. The small sample size and lack of control group further limit the generalisability of these findings.

For peer review only

Chaudhry's pilot study formed the basis of an RCT conducted by Gater et al.(54) who recruited British Pakistani women (n=123) with depression, diagnosed through structured clinical interviews (revised Clinical Interview Schedule), from general practice surgeries. The women were randomised into one of three intervention arms: social group intervention, antidepressant, or both. Like the pilot study, the social group intervention involved a small network of women attending 10 weekly sessions of social activities. These took place at a community centre and were facilitated by Urdu-speaking women who had completed a five-day training programme. Refreshments and free transportation were provided.

There was no significant change in depression at three and nine months in all three arms of the RCT. There was significantly greater improvement in social functioning in the social intervention and combined treatment groups at three months (6.1, 95% CI: 1.4,10.8 and 5.9, 95% CI:1.5,10.2), however this effect did not persist at 9 months. Focus groups, held at the end of each session, found the intervention to be too short and that a major barrier to participating was resistance from family members. Participants found it helpful to confide in others and intended to maintain new friendships. Overall, this study was appraised as low risk and provides evidence in favour of social group interventions for short-term improvement of positive mental health.

Promoting access and use of services

Gray(58) describes the findings of an ethnographic investigation into the perceptions of the Family Support Service, a project operating in Tower Hamlets in London. The service encompasses three projects including; 'Family Support', where home-based

For peer review only - http://bmjopen.bmj.com/site/about/guidelines.xhtml

support is provided to families including parenting skills; 'Building Bridges', where families with a parent with a severe mental illness are supported; and 'Quality Protects', where support workers help families together with the local authority in assessment and child protection. The service was provided by Family Support Workers(FSWs) who were recruited from the local community or had similar backgrounds to the client group.

30 case records of families were examined, and 22 interviews were conducted with FSWs, families themselves, and professionals managing or referring to these services. FSWs were found to have had a positive impact on families in various ways, including bringing families together; reducing social isolation, bullying and racism; promoting equality of access and pertinent use of services; and liaising successfully with other health and social care services. It was highly valued that projects were culturally sensitive and that FSWs were of the same ethnicity. Gray establishes the importance of power dynamics in the intervention, as facilitators' decisions have potentially significant consequence to the families. This makes the FSWs not only essential to delivering the intervention but also integral to achieving a positive outcome for families.

Jacob et al.(56) conducted an RCT in one general practice in Ealing, London evaluating the effect of a culturally-appropriate educational leaflet on depression for women of Indian, Bangladeshi and Pakistani heritage (n=70). Only women diagnosed with depression, through a two-phase procedure using the Self-Reporting Questionnaire (SRQ) and the Clinical Interview Schedule–Revised(CIS–R), were included in the study. The included participants then had their perspective

For peer review only

(explanatory models) on depression assessed using a Short Explanatory Model Interview (SEMI). Participants were then randomly allocated to receive a leaflet about the nature, causes, prevalence and treatment of depression or to usual care from their general practitioner. The leaflet was available in English, Hindi and Punjabi and read out to illiterate participants, yet only those conversant in Hindi and English were included. After two months all participants repeated the General Health Questionnaire (GHQ) and re-discussed the SEMI, particularly focusing on whether depression was seen as an illness and if medical help was necessary. There was no statistically significant difference between the intervention and control group on the explanatory model of depression at follow-up. The number of women who achieved recovery from depression (defined as GHQ-12 $\leq$ 2) at two months was significantly associated with receiving the leaflet (42.9% vs. 20% for controls, $p<0.05$; no median score of confidence intervals reported). However, this was also significantly associated with lower level of psychiatric morbidity at entry into the trial. As such, the mechanism of this improvement is unclear.

Rabiee et al.(57) conducted a survey questionnaire(n=257) and focus groups(n=4) to evaluate the physical and mental health impact of a Gym for Free scheme in a deprived constituency in Birmingham. Participants were asked to fill in a survey detailing various physical characteristics, frequency of exercise, the motivations for joining the gym and perceived emotional benefits. Benefits of the scheme included improved confidence(n=92) and energy(n=121), reduced stress(n=77), and improved anger management(n=8).

For peer review only - http://bmjopen.bmj.com/site/about/guidelines.xhtml

Nine individuals participated in focus groups, including two men (one White and one Black) and seven women (four Pakistani, two Indian and one White). These participants valued the intervention taking place within the community as it helped build social networks. The link between physical and mental well-being was acknowledged and the scheme was praised for enabling these discussions. Gender of focus group respondents, but not their ethnicity, was coded to avoid identifying respondents. Although the authors suggest a financial barrier es that inhibits people joining the gym, no formal data were collected on cost or participant finances. The generalisability of these findings is limited as participants were selected through opportunistic sampling and may over-represent younger adults motivated to use the gym.

Lay health workers delivering complex interventions

Afuwape et al.(52) conducted an RCT testing the feasibility, efficacy and cultural acceptability of the London Cares of Life Project(CoLP). CoLP is a complex social intervention designed to improve the mental health of adults of black African or black Caribbean origin(n=40). Participants with anxiety and/or depression, diagnosed using World Health Organisation checklist criteria, were randomised to receive the CoLP intervention immediately ('rapid access') or to three month waiting list control with information on local mental health services ('standard access'). Over three months, community health workers (psychology graduates trained to deliver the intervention) provided: practical advice to address social needs, health education; and gave brief therapies based on cognitive behavioural therapy principles. At three-month follow-up, individuals in the rapid access group showed significantly improved levels of depressive symptoms (adjusted mean difference=7.76, CI 95%: 0.86 to 14.65)

For peer review only - http://bmjopen.bmj.com/site/about/guidelines.xhtml

although there was no evidence of improved general functioning (adjusted mean difference= -0.78, CI 95%: -10.40 to 8.84). This study found no difference in costings between the intervention and control group; health and social care utilisation costs were calculated for three months.

The long-term effect of the intervention is unknown; follow-up was limited to three months. The risk of bias is increased by the small sample size, which meant that despite randomisation, baseline characteristics differed; participants in the non-intervention arm had a significantly greater prevalence of past psychiatric history. The generalisability to the wider BAME population is potentially limited due to the exclusion of non-English speaking participants and the screening tools used not having clear evidence of validity in these populations.

A pilot RCT by Lovell et al.(53) examined the effect of a wellbeing intervention, based on brief cognitive behavioural strategies, aimed at decreasing anxiety, depression and social isolation in two separate groups: BAME populations (aged 21-58 years, n=20) and the elderly (95.7% White, n=37). Only the results from the BAME group were included in this analysis. Participants chose between receiving the intervention individually, as part of a group, or receiving signposting sessions. Interventions in all three arms were delivered by wellbeing facilitators, after a three-day training programme, who liaised with health and social care professionals involved in the participant's care. Individual interventions comprised eight 30-minute sessions over 16 weeks, groups were delivered 8-10 weekly two-hour sessions, and those who participated in signposting were seen three times over a 16-week period.

For peer review only - http://bmjopen.bmj.com/site/about/guidelines.xhtml

This intervention had no statistically significant effect on global distress, common mental disorder, anxiety, social function or health related quality of life. Semi-structured interviews findings established that involvement of family and community, a health professional's recommendation, and having an empathetic facilitator (deemed more important than sharing ethnicity) were important factors in the intervention's acceptability.

This study is limited by its small sample size of ethnic minority participants (n=20). Additionally, the element of patients choosing their intervention arm complicates the analysis of the mechanism of effect, albeit reflecting the realities of engagement and participation when delivering interventions.

For peer review only

Discussion

Main findings

We found seven studies of community-centred interventions that aimed to improve mental health and wellbeing among BAME groups in the UK. Although studies varied in design, four key interventional characteristics emerged.

First, interventions aimed to address social isolation through building peer-to-peer support and social networks. Existing research suggests that people from BAME populations find it easier and more appropriate to talk within their social network rather than to professionals; formal mental health services are perceived as a last resort(63,64). We found that social support interventions mainly targeted women and it is unclear whether men would find these acceptable.

Second, interventions were focussed on overcoming cultural barriers in accessing care using facilitators that shared the same ethnicity and or life experience as participants. Healthcare providers that are language and culturally naïve are a barrier to BAME groups in effectively communicating their mental health needs (10,65,66). This concurs with emerging evidence that culturally adapted interventions lead to better outcomes among minority ethnic groups(67,68). Addressing practical considerations, such as travel, also increased participation.

Third, interventions were delivered by lay health workers; over recent years, task-shifting in healthcare from health professionals to lay health workers has been common due to the need to meet escalating demand for care(69). Lay health workers

For peer review only - http://bmjopen.bmj.com/site/about/guidelines.xhtml

from the same community as patients are perceived as more accessible(70) and help reduce the associated stigma of accessing mental health services (71,72).

Four, interventions were complex (that is containing several interacting components) with a strong emphasis on signposting and or facilitating linkages to complementary, or additional services. Signposting has been widely adopted in England through social prescribing schemes, but to date there has been little emphasis on cultural appropriateness(73). Evidence suggests that mental health services provided by the voluntary and community sector and embedded in communities increases trust among BAME communities, which in turn promotes awareness of mental health problems and access to mental health services(16).

Most of the studies focussed on people with existing common mental conditions rather than promoting positive mental health. Health promotion and primary prevention alongside universal approaches are critical components of strong public mental health and sustainable health systems(74). We found community interventions focussed on specific groups, mainly South Asian and Afro-Caribbean populations and there were no interventions designed for and targeting men, despite men being less likely to seek help for common mental health problems (64,75).

Limitations

A limitation of this study was the omission of grey literature. Preliminary grey literature searching, which covered two local authority boroughs, found over 50 community-centred services. However, there were no evaluation data. This confirmed that the bulk of relevant interventions reside outside publication in peer review journals. We

For peer review only - http://bmjopen.bmj.com/site/about/guidelines.xhtml

also only included community-centred interventions that targeted BAME groups. It is possible that BAME groups access public mental health interventions through universal provision, yet uptake of screening programmes and utilisation of mental health care services shows BAME populations have lower use(76).

A further limitation was excluding all white ethnic minority groups apart from Irish Travellers. With increasing migration, particularly from Europe, there is a growing white ethnic minority population subject to similar systemic health inequalities and challenges to mental health and well-being as other BAME groups. This evidence gap was also found in a recently published review on racial disparities in mental health(16). Asylum seekers and migrants were also not included in this study. While there is a body of research on community-centred interventions for this population that likely has important overlapping components, research indicates that more specialised interventions with a greater focus on trauma reduction are needed(77,78).

Implications for policy makers and future research

We recognise that the evidence for ethnic differences in some common mental disorders is complex, variable and indeed equivocal for specific ethnic groups. However, there is a disconnect between the scale of community-centred intervention provisions and published evidence that may imply a bias in terms of scientific priorities and funding that needs urgent rectification, as current evidence is limited and weak.

It is important that future research seeks to understand how successful interventions work and for whom, taking into account intersectionality such as between gender and ethnicity and its associated disadvantage. There is a need to identify successful

For peer review only - http://bmjopen.bmj.com/site/about/guidelines.xhtml

interventions that can be scaled and implemented elsewhere(79). Third sector organisations who provide these interventions are often bound to deliver short-term projects and lack resources for robust evaluations. It is important that mental healthcare commissioners provide sufficient resources to evaluate services from the onset of delivery including economic analysis; community interventions are potentially an affordable means to improving public mental health(80).

Labels such as ‘Asian’ or ‘Black African’ present significant and ongoing challenges to mental health research by viewing ethnic minority groups as homogenous in their mental health needs, despite evidence indicating otherwise(81-83). We recommend that national census categories are consistently used across all sectors, so that important variations and inequities can be identified and investigated further.

Conclusion

Despite well-documented ethnic disparities in mental health, there is a paucity of high-quality evidence regarding community-based interventions that focus on improving public mental health among BAME groups. Decision makers need scientific evidence to help commission appropriate services and to inform effective approaches to mitigating these health disparities. Our next steps are to map the promising community activities and interventions that are currently being provided to help identify emerging evidence.

For peer review only - http://bmjopen.bmj.com/site/about/guidelines.xhtml

Acknowledgements

Many thanks to the library staff at Imperial College London for their insights in the design of search terms and their support in the use of resources.

Funding Statement

This study is funded by the National Institute for Health Research (NIHR) School for Public Health Research (SPHR) (Grant Reference Number PD-SPH-2015). The views expressed are those of the author(s) and not necessarily those of the NIHR or the Department of Health and Social Care.

The NIHR School for Public Health Research is a partnership between the Universities of Sheffield; Bristol; Cambridge; Imperial; and University College London; The London School for Hygiene and Tropical Medicine (LSHTM); LiLaC - a collaboration between the Universities of Liverpool and Lancaster; and Fuse - The Centre for Translational Research in Public Health a collaboration between Newcastle, Durham, Northumbria, Sunderland and Teesside Universities.

Competing Interests

The authors have no competing interests to declare.

Authors' Contributions

SG conceived the idea. GZ, CB and SG designed and conducted the study, which was supervised by SG. GZ and CB wrote the original draft of the manuscript. Reviewing and editing was undertaken by GZ, CB and SG. All authors contributed to interpretation of the results and commented on the final manuscript.

For peer review only

References

1. Bhui KS, McKenzie K. Rates and Risk Factors by Ethnic Group for Suicides Within a Year of Contact With Mental Health Services in England and Wales. *Psychiatr Serv*. 2008 Apr;59(4):414–20.doi: 10.1176/ps.2008.59.4.414
2. Weich S, Nazroo J, Sporston K, et al. Common mental disorders and ethnicity in England: The EMPIRIC study. *Psychol Med*. 2004 Nov;34(8):1543–51.doi: 10.1017/s0033291704002715
3. Williams ED, Tillin T, Richards M, et al. Depressive symptoms are doubled in older British South Asian and Black Caribbean people compared with Europeans: Associations with excess co-morbidity and socioeconomic disadvantage. *Psychol Med*. 2015;45(9):1861–71.doi: 10.1017/S0033291714002967
4. Adkison-Bradley C, Maynard D, Johnson P, Carter S. British African Caribbean women and depression. *Br J Guid Couns*. 2009 Feb;37(1):65–72.doi: 10.1080/03069880802535887
5. Ngwena J. Black and minority ethnic groups (BME) suicide, admission with suicide or self-harm: An inner city study. *J Public Heal*. 2014;22(2):155–63.doi: 10.1007/s10389-013-0600-9
6. Karlsen S, Nazroo JY, McKenzie K, et al. Racism, psychosis and common mental disorder among ethnic minority groups in England. *Psychol Med*. 2005 Dec;35(12):1795–803.doi: 10.1017/S0033291705005830
7. Powell A, Francis-Devine B, Foley N. Unemployment by ethnic background. House of Commons Library. 2020.
8. Barnard H, Turner C. Poverty and ethnicity: A review of evidence. York:JRF.
9. Wallace S, Nazroo J, Bécares L. Cumulative effect of racial discrimination on the mental health of ethnic minorities in the United Kingdom. *Am J Public Health*. 2016 Jul 1;106(7):1294–300.doi: 10.2105/AJPH.2016.202121
10. Memon A, Taylor K, Mohebati LM, et al. Perceived barriers to accessing mental health services among black and minority ethnic (BME) communities: A qualitative study in Southeast England. *BMJ Open*. 2016 Nov 1;6(11):e012337.

For peer review only

11. Mantovani N, Pizzolati M, Edge D. Exploring the relationship between stigma and help-seeking for mental illness in African-descended faith communities in the UK. *Heal Expect*. 2017 Jun;20(3):373–84.doi: 10.1111/hex.12464.

12. Williams ED, Kooner I, Steptoe A, et al. Psychosocial factors related to cardiovascular disease risk in UK South Asian men: A preliminary study. *Br J Health Psychol*. 2007;12(4):559–70.doi: 10.1348/135910706X144441

13. Uhrig N. Black, Asian and Minority Ethnic disproportionality in the Criminal Justice System in England and Wales. Ministry of Justice Analytical Services. 2016.

14. Lyratzopoulos G, Elliott M, Barbiere JM, et al. Understanding ethnic and other socio-demographic differences in patient experience of primary care: Evidence from the English General Practice Patient Survey. *BMJ Qual Saf*. 2012;21(1):21–9.doi: 10.1136/bmjqs-2011-000088. Epub 2011 Sep 7.

15. Morgan C, Fearon P, Lappin J, et al. Ethnicity and long-term course and outcome of psychotic disorders in a UK sample: The ÆSOP-10 study. *Br J Psychiatry*. 2017 Aug 2;211(2):88–94.doi: 10.1192/bjp.bp.116.193342

16. Bignall T, Jeraj S, Helsby E, et al. Racial disparities in mental health: literature and evidence review — NHS Networks. 2020 [cited 2020 Mar 19]. Available from: https://www.networks.nhs.uk/news/racial-disparities-in-mental-health-literature-and-evidence-review

17. Definitions | Institute of Race Relations. [cited 2020 May 6]. Available from: http://www.irr.org.uk/research/statistics/definitions/

18. Glover G, Evison F. Use of new Mental Health Services by Minority Ethnic Groups in England. England: North East Public Health Observatory. 2009.

19. Local Government Improvement and Development. Culturally responsive JSNAs: a review of race equality and Joint Strategic Needs Assessment (JSNA) practice. 2010.

20. Appleby L, Asherson P, Aznar C, et al. Mental Health and Wellbeing in England: Adult Psychiatric Morbidity Survey 2014. NHS Digital. 2014.

21. Fearon P, Kirkbride JB, Morgan C, et al. Incidence of schizophrenia and other psychoses in ethnic minority groups: Results from the MRC AESOP Study. Psychol Med. 2006 Nov;36(11):1541–50.doi: 10.1017/S0033291706008774.

22. McKenzie K, Bhui K, Nanchahal K, et al. Suicide rates in people of South Asian origin in England and Wales: 1993-2003. *Br J Psychiatry*. 2008

For peer review only

For peer review only - http://bmjopen.bmj.com/site/about/guidelines.xhtml

Nov;193(5):406–9.doi: 10.1192/bjp.bp.107.042598.

23. Kirkbride J, Hameed Y, Ioannidis K, et al. Ethnic minority status, age-at-immigration and psychosis risk in rural environments: evidence from the SEPEA study. *Schizophr Bull.*2017 Oct 21;43(6):1251-1261.doi: 10.1093/scbul/sbx010.
24. Fearon P, Kirkbride JB, Morgan C, et al. Incidence of schizophrenia and other psychoses in ethnic minority groups: results from the MRC AESOP Study. Incidence of schizophrenia and other psychoses in ethnic minority groups: results from the MRC AESOP Study. *Psychol Med.* 2006 Nov; 36(11):1541-1550.doi: 10.1017/S0033291706008774
25. Baker C. Mental health statistics: prevalence, services and funding in England. House of Commons Library. 2018.
26. Public mental health - GOV.UK [Internet]. [cited 2020 Mar 19]. Available from: https://www.gov.uk/government/collections/public-mental-health
27. Davies SC. Annual Report of the Chief Medical Officer: Public Mental Health Priorities: Investing in the Evidence. 2014.
28. Department of Health. Delivering race equality in mental health care, an Action Plan for reform inside and outside services; and the Government's response to the indpeendent inquiry into the death of David Bennett. London: 2005.
29. Care Quality Commission. Count me in 2010: Results of the 2010 national census of inpatients and patients on supervised community treatment in mental health and learning disability services in England and Wales. London: 2011.
30. Salway S, Holman D, Lee C, et al. Transforming the health system for the UK's multiethnic population. *BMJ*. 2020; 368:m268
31. Health matters: community-centred approaches for health and wellbeing - GOV.UK [Internet]. [cited 2020 Mar 16]. Available from: https://www.gov.uk/government/publications/health-matters-health-and-wellbeing-community-centred-approaches/health-matters-community-centred-approaches-for-health-and-wellbeing
32. NICE. Impact Mental Health. NICE 2019.
33. Peters MDJ, Godfrey CM, Khalil H, et al. Guidance for conducting systematic scoping reviews. *Int J Evid Based Healthc*. 2015;13(3):141–6.doi: 10.1097/XEB.0000000000000050

For peer review only

For peer review only - http://bmjopen.bmj.com/site/about/guidelines.xhtml

34. Tricco AC, Lillie E, Zarin W, et al. A scoping review on the conduct and reporting of scoping reviews. *BMC Med Res Methodol.* 2016;16(1):1–10.doi: 10.1186/s12874-016-0116-4
35. Arksey H, O'Malley L. Scoping studies: Towards a methodological framework. *Int J Soc Res Methodol Theory Pract*. 2005 Feb;8(1):19–32.doi: 10.1080/1364557032000119616
36. Public mental health programme - NIHR School for Public Health Research NIHR SPHR [Internet]. [cited 2020 May 6]. Available from: https://sphr.nihr.ac.uk/category/research/public-mental-health/
37. Booth A, Clarke M, Dooley G, et al. The nuts and bolts of PROSPERO: An international prospective register of systematic reviews. *Syst Rev.* 2012 Feb 9;1;2.doi: 10.1186/2046-4053-1-2.
38. 50 years of collecting ethnicity data - History of government [Internet]. [cited 2020 Apr 7]. Available from: https://history.blog.gov.uk/2019/03/07/50-years-of-collecting-ethnicity-data/
39. Adult Psychiatric Morbidity Survey: Survey of Mental Health and Wellbeing, England, 2014. NHS Digital. [cited 2020 Mar 20]. Available from: https://webarchive.nationalarchives.gov.uk/20180328140249/http://digital.nhs.uk/catalogue/PUB21748
40. National Institute for Health and Care Excellence. Common mental health problems: identification and pathways to care. 2011.
41. Porter M, Haslam N. Predisplacement and postdisplacement of refugees and internally displaced persons. *J Am Med Assoc*. 2005;294(5):610–2.doi: 10.1001/jama.294.5.602
42. Steel Z, Chey T, Marnane C, et al. Association of Torture and Other Potentially Traumatic Events With Mental Health Outcomes Among Populations Exposed to Mass Conflict and Displacement: A Systematic Review and Meta-analysis. *J Am Med Assoc*. 2019;302(5).doi: 10.1001/jama.2009.1132.
43. Depression in adults: recognition and management - Clinical Guideline 90. 2009.
44. Generalised anxiety disorder and panic in adults: management - Clinical guideline 113. 2011.
45. National Office for Statistics. 2011 Census Analysis: Ethnicity and Religion of the Non-UK Born Population in England and Wales. 2015;(June):5.

For peer review only - http://bmjopen.bmj.com/site/about/guidelines.xhtml

46. Veritas Health Innovation. Covidence systematic review software. Veritas Health Innovation, Melbourne, Australia.

47. Hoffmann TC, Glasziou PP, Boutron I, Milne R, Perera R, Moher D, et al. Better reporting of interventions: Template for intervention description and replication (TIDieR) checklist and guide. *BMJ*. 2014 Mar;348:g1687

48. Higgins JP, Altman DG. Assessing Risk of Bias in Included Studies. In: Cochrane Handbook for Systematic Reviews of Interventions: Cochrane Book Series. 2008.

49. Noyes J, Booth A, Flemming K, et al. Cochrane Qualitative and Implementation Methods Group guidance series—paper 3: methods for assessing methodological limitations, data extraction and synthesis, and confidence in synthesized qualitative findings. *J Clin Epidemiol*. 2018 May;97:49–58.doi: 10.1016/j.jclinepi.2017.06.020. Epub 2017 Dec 13.

50. Pluye P, Gagnon MP, Griffiths F, et al. A scoring system for appraising mixed methods research, and concomitantly appraising qualitative, quantitative and mixed methods primary studies in Mixed Studies Reviews. Int J Nurs Stud. 2009 Apr; 46 (4):529-46.doi: 10.1016/j.ijnurstu.2009.01.009.Epub 2009 Feb 23.

51. Tricco AC, Lillie E, Zarin W, et al. PRISMA extension for scoping reviews (PRISMA-ScR): Checklist and explanation. Vol. 169, Annals of Internal Medicine. American College of Physicians; 2018. p. 467–73.

52. Afuwape SA, Craig TKJ, Harris T, et al. The Cares of Life Project (CoLP): An exploratory randomised controlled trial of a community-based intervention for black people with common mental disorder. *J Affect Disord*. 2010;127(1–3):370–4.doi: 10.1016/j.jad.2010.05.017. Epub 2010 Jun 14.

53. Lovell K, Lamb J, Gask L, et al. Development and evaluation of culturally sensitive psychosocial interventions for under-served people in primary care. *BMC Psychiatry*. 2014 Aug 1;14:217.doi: 10.1186/s12888-014-0217-8.

54. Gater R, Waheed W, Husain N, Tomenson B, Aseem S, Creed F. Social intervention for British Pakistani women with depression: Randomised controlled trial. *Br J Psychiatry.* 2010;197(3):227–33.doi: 10.1192/bjp.bp.109.066845.

55. Chaudhry N, Waheed W, Husain N, et al. Development and pilot testing of a social intervention for depressed women of Pakistani family origin in the UK. *J*

For peer review only

Ment Heal. 2009;18(6):504–9.doi: 10.3109/09638230902968209.

56. Jacob KS, Bhugra D, Mann AH. A Randomised Controlled Trial of an Educational Intervention for Depression Among Asian Women in Primary Care in the United Kingdom. *Int J Soc Psychiatry*. 2002 Jun;48(2):139-48.doi: 10.1177/002076402128783190.
57. Rabiee F, Robbins A, Khan M. Gym for Free: The short-term impact of an innovative public health policy on the health and wellbeing of residents in a deprived constituency in Birmingham, UK. *Health Educ J.* 2015;74(6):691–704.
58. Gray B. Social Exclusion, Poverty, Health and Social Care in Tower Hamlets: The Perspectives of Families on the Impact of the Family Support Service. *Br J Soc Work*. 2003;33(3):361–80.doi: 10.1093 bjsw33.3.361
59. Krueger R, Casey M. Focus groups: A practical guide for applied research. Thousand Oaks, CA: Sage Publications 2000.
60. Rabiee F. Focus-group interview and data analysis. *The Proceedings of the Nutrition Society*. 2004, 63(4):655-660.doi: 10.1079/pns2004399
61. Garfinkel E, Cliffs H. Studies in ethnomethodology.1967.
62. Glaser BG, Strauss AL. Discovery of grounded theory: Strategies for qualitative research. Discovery of Grounded Theory: Strategies for Qualitative Research. Taylor and Francis; 1967. 1–271 p.
63. Tabassum R, Macaskill A, Ahmad I. Attitudes towards mental health in an urban Pakistani community in the United Kingdom. *Int J Soc Psychiatry*. 2000;46(3):170–81.doi: 10.1177/002076400004600303.
64. Sheikh S, Furnham A. A cross-cultural study of mental health beliefs and attitudes towards seeking professional help. *Soc Psychiatry Psychiatr Epidemiol*. 2000;35(7):326–34.doi: 10.1007/s001270050246.
65. Tabassum R, Macaskill A. Attitudes towards mental health in an urban Pakistani community in the United Kingdom. *Int J Soc Psychiatry*. 2000;46(3):170–81.doi: 10.1177/002076400004600303.
66. Li PL, Logan S, Yee L, et al. Barriers to meeting the mental health needs of the Chinese community. *J Public Health Med*. 1999 Mar;21(1):74–80.doi: 10.1093/pubmed/21.1.74.
67. Chowdhary N, Jotheeswaran AT, Nadkarni A, et al. The methods and outcomes of cultural adaptations of psychological treatments for depressive disorders: a systematic review. *Psychol Med.* 2014 Apr;44(6):1131-46.doi:

For peer review only

10.1017/S0033291713001785. Epub 2013 Jul 19.

68. Benish S, Quintana S, Wampold B. Culturally Adapted Psychotherapy and the Legitimacy of Myth: A Direct-Comparison Meta-Analysis. *J Couns Psychol*. 2011 Jul;58(3):279-89.doi: 10.1037/a0023626.

69. Shahmalak U, Blakemore A, Waheed MW, et al. The experiences of lay health workers trained in task-shifting psychological interventions: a qualitative systematic review. *Int J Ment Health Syst*. 2019;13:64.doi: 10.1186/s13033-019-0320-9.

70. Singla D, Lazarus A, Atif N, et al. “Someone like us”: Delivering maternal mental health through peers in two South Asian contexts. *J Affect Disord.*2014 Oct;168:452-8.doi: 10.1016/j.jad.2014.07.017.

71. McClay CA, Morrison J, McConnachie A, et al. A community-based group-guided self-help intervention for low mood and stress: Study protocol for a randomized controlled trial. *Trials.* 2013; 14:392.doi: 10.1186/1745-6215-14-392.

72. Mueller M, Kennerley H, McManus F et al. Oxford Guide to Surviving as a CBT Therapist. Oxford University Press 2010.

73. Bickerdike L, Booth A, Wilson PM, et al. Social prescribing: Less rhetoric and more reality. A systematic review of the evidence. *BMJ Open*. 2017 Apr;7(4).doi:10.1136/bmjopen-2016-013384

74. Mcdaid D, Park A-L, Wahlbeck K. The Economic Case for the Prevention of Mental Illness. *Annu Rev Public Heal*. 2019 Apr 1;40:373–89.doi: 10.1146/annurev-publhealth-040617-013629. Epub 2019 Jan 2.

75. Oliver MI, Pearson N, Coe N, et al. Help-seeking behaviour in men and women with common mental health problems: Cross-sectional study. *Br J Psychiatry*. 2005 Apr;186:297-301.doi: 10.1192/bjp.186.4.297.

76. Marlow LAV, Wardle J, Waller J. Understanding cervical screening non-attendance among ethnic minority women in England. *Br J Cancer*. 2015 Sep 1;113(5):833–9. doi: 10.1038/bjc.2015.248.

77. Byrow Y, Pajak R, McMahon T, et al. Barriers to mental health help-seeking amongst refugee men. *Int J Environ Res Public Health*. 2019 Aug 1;16(15):2634.doi: 10.3390/ijerph16152634.

78. Hynie M. The Social Determinants of Refugee Mental Health in the Post-Migration Context: A Critical Review. *Can J Psychiatry.* 2018 May;63(5):297-

For peer review only - http://bmjopen.bmj.com/site/about/guidelines.xhtml

303.doi: 10.1177/0706743717746666.

79. Pawson R, Greenhalgh T, Harvey G, et al. Realist review - A new method of systematic review designed for complex policy interventions. J Health Serv Res Policy. 2005 Jul;10 Suppl 1:21-34.doi: 10.1258/1355819054308530

80. McLaughlin KA. The Public Health Impact of Major Depression: A Call for Interdisciplinary Prevention Efforts, *Prevention Science*. 2011 Nov; 12(4): 361-371. doi: 10.1007/s11121-011-0231-8

81. Care Quality Commission. Count me in: 2010 Results of the 2010 national census of inpatients and patients on supervised community treatment in mental health and learning disability services in England and Wales. London, UK: Care Quality Commission; 2011.

82. Care Quality Commission. Count Me In: Results of the 2009 National Census of Inpatients and Patients on Supervised Community Treatment Orders in Mental Health and Learning. London, UK: Care Qiuality Commission; 2010.

83. Weich S, Nazroo J, Sprostoin K, et al. Common mental disorders and ethnicity in England: the EMPIRIC Study. *Psychol Med.* 2004; 34(8):1543-1551.doi:10.1017/s0033291704002715

For peer review only

Figure 1. PRISMA flow chart of search strategy

Legend: The PRIMSA diagram details our search and selection process applied during the overview.

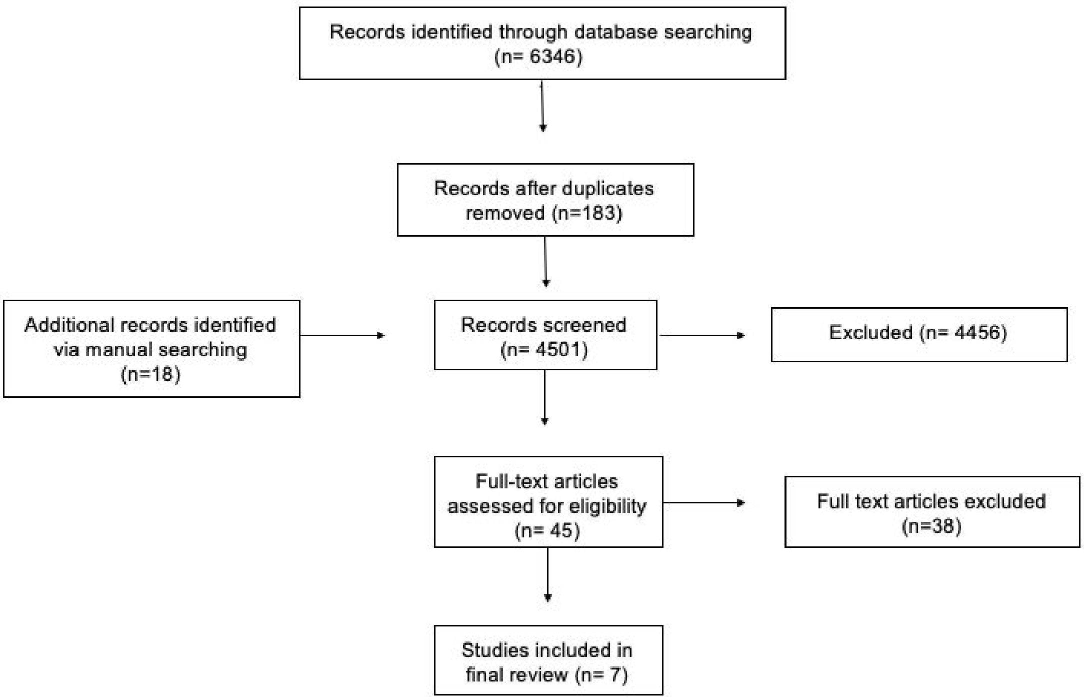

view only

Appendix 1. Search strategy in all databases

Database Searched	Ethnicity Terms	Outcome Terms (Mental Health)	Geography Terms	Population terms
**Medline**	minority groups.mp. OR Minority Groups/ OR ethnic groups.mp. OR exp Ethnic Groups/ OR ethnic*.mp. OR Race.mp. OR exp Continental Population Groups/ OR racial.mp. OR<br><br>Black.mp. OR exp African Continental Ancestry Group/ OR Black African*.mp OR British African*.mp OR (Black adj1 British).mp. OR Afro$Caribbean*.mp OR Black adj1 caribbean.mp OR (Afr* adj3 Caribbean) OR Black*.mp. OR<br><br>Asian.mp. OR exp Asian Continental Ancestry Group/ OR Indian*.mp. OR exp Pakistan/ OR Pakistan*.mp. OR Bangladesh*.mp. OR exp Bangladesh/ OR Bengali*.mp/ OR Chinese.mp. OR<br><br>Arab*.mp. OR exp Arabs OR	Mental Health.mp. OR exp Mental Health/ OR mental well$being.mp OR exp Stress, Psychological/ OR psychological well$being.mp. OR Resilience, Psychological/ OR resilien*.mp. OR exp Occupational Stress OR stress.mp.	exp United Kingdom/ OR UK.mp OR United Kingdom.mp. OR Great Britain.mp OR Britain.mp OR GB.mp OR England.mp OR Scotland.mp OR Wales.mp OR North* Ireland.mp OR British.mp OR Scottish.mp OR Welsh.mp OR North* Irish.mp	Adult*<br>Wom?n<br>Men<br>people

For peer review only - http://bmjopen.bmj.com/site/about/guidelines.xhtml

	Gypsy or "Irish traveller" OR<br><br>(white and black African*).mp. OR (white and black caribbean).mp. OR (white and Asian).mp. OR mixed ethnicit*.mp			
**Embase**	minority groups.mp. OR exp Minority Groups/ OR ethnic groups.mp. OR exp Ethnic Group/ OR ethnic*.mp. OR exp race/ OR "ethnic or racial aspects"/ OR Race.mp. OR racial.mp. OR<br><br>Black*.mp. OR exp Black person/ OR black african*.mp OR British African*.mp OR (Black adj1 British).mp. OR Afro$Caribbean*.mp OR (Black adj1 caribbean).mp OR (Afr* adj3 Caribbean).mp. OR<br><br>Asian.mp. OR exp Asian/ OR exp Asian continental ancestry group/ OR Indian*.mp. OR exp Indian/ OR Pakistan*.mp. OR exp Pakistan/ OR exp Bangladesh/ OR Bangladesh*.mp. OR Bengali*.mp OR exp "Bengali (people)"/ OR Chinese.mp. OR exp Chinese/ OR	Mental Health.mp. OR exp Mental Health/ OR [mental well$being.mp] OR exp psychological well-being/ OR psychological stress.mp. OR exp mental Stress/OR psychGological well$being.mp. OR exp psychological resilience/ OR resilien*.mp OR job stress/ OR life stress/ OR chronic stress/ OR emotional stress/ OR social stress/ OR behavioural stress/ OR stress.mp.	Exp united Kingdom/ OR United Kingdom.mp. OR UK.mp. OR Great Britain.mp OR Britain.mp OR GB.mp OR England.mp OR Scotland.mp OR Wales.mp OR North* Ireland.mp OR British.mp OR Scottish.mp OR Welsh.mp OR North* Irish.mp	Adult* Wom?n Men people

For peer review only - http://bmjopen.bmj.com/site/about/guidelines.xhtml

	Arab*.mp. OR exp Arab/ OR<br><br>Gypsy or “Irish traveller” OR<br><br>(white and black African*).mp. OR (white and black Caribbean*).mp. OR (white and Asian*).mp.] OR mixed ethnic*.mp			
**PsychINFO**	minority groups.mp. OR exp Minority Group/ OR ethnic groups.mp. OR exp ‘Racial and Ethnic groups’/ OR ethnic*.mp. OR Race.mp. OR racial.mp. OR ‘race and ethnic discrimination’/ OR<br><br>exp Blacks/ OR black*.mp. OR exp African Cultural Groups/ or black African*.mp. OR British African*.mp OR (Black adj1 British).mp. OR Afro$Caribbean*.mp OR (Black adj1 caribbean).mp OR (afr* adj3 Caribbean).mp. OR Asian.mp. OR exp Asians/ OR Indian*.mp. OR Pakistan*.mp. OR Bangladesh*.mp. OR Bengali*.mp OR Chinese.mp. OR<br><br>Arab*.mp. OR<br><br>Gypsy or “Irish traveller” OR	Mental Health.mp. OR exp Mental Health/ OR mental well$being.mp OR exp Well Being/ OR exp Psychological Stress/or psychological well$being.mp. OR exp “Resilience (Psychological)”/OR resilien*.mp. OR exp Occupational Stress/ OR stress.mp. OR exp Chronic stress/ OR exp Social Stress/	United Kingdom.mp. OR UK.mp. OR Great Britain.mp OR Britain.mp OR GB.mp OR England.mp OR Scotland.mp OR Wales.mp OR North* Ireland.mp OR British.mp OR Scottish.mp OR Welsh.mp OR North* Irish.mp	Adult*<br>Wom?n<br>Men<br>people

For peer review only - http://bmjopen.bmj.com/site/about/guidelines.xhtml

	(white and black African*).mp. OR (white and black Caribbean*).mp. OR (white and Asian*).mp. OR<br><br>mixed ethnicit*.mp			
**SCOPUS**	("minority group*" OR ethnic* OR race OR racial OR african* OR (black W/1 british) OR afro$caribbean* OR (black W/1 caribbean) OR indian OR pakistani OR arab * OR asian OR indian OR bangladesh* OR bengali OR chinese OR "mixed ethnic*" OR "mixed race")	("mental health" OR "mental well?being" OR "psychological well?being" OR "psychological stress" OR "psychological resilien*")	"united kingdom" OR "Great Britain" OR England OR Scotland OR Wales OR (North* Ireland) OR British OR Scottish OR Welsh OR (North* Irish)	Adult*<br>Wom?n<br>Men<br><br>people
**CINAHL**	(minority group*) OR (ethnic* group*) OR ethnic* OR race OR racial OR "black African" OR "African Cultural Groups" OR (british W/1 african) OR (black W/1 british) OR Afro$ Caribbean* OR (black W/1 caribbean) OR (afr* W/3 caribbean) OR asian OR indian OR pakistan* OR bangladesh* OR bengali OR chinese OR arab* OR	(Mental Health) OR (mental well?being) OR (Well?Being) OR (Psychological Stress) OR (psychological well?being) OR exp (psychological resilien*) OR (Chronic stress) OR (Social Stress)	"united kingdom" OR "Great Britain" OR England OR Scotland OR Wales OR (North* Ireland) OR British OR Scottish OR Welsh OR (North* Irish)	Adult*<br>Wom?n<br>Men<br><br>people

For peer review only - http://bmjopen.bmj.com/site/about/guidelines.xhtml

	“mixed ethnicit*” or Gypsy or “Irish traveller”			
**Cochrane**	"minority group*" OR ethnic* OR race OR racial OR african* OR (black near/1 british) OR afro$caribbean* OR (black near/1 caribbean) OR asian OR indian OR pakistan* OR bangladesh* OR bengali* OR chinese OR arab* OR "mixed ethnicit*" OR gypsy OR “Irish traveller”	(Mental Health) OR (mental well?being) OR (Well?Being) OR (Psychological Stress) OR (psychological well?being) OR exp (psychological resilien*) OR (Chronic stress) OR (Social Stress)	“united kingdom” OR “Great Britain” OR England OR Scotland OR Wales OR (North* Ireland) OR British OR Scottish OR Welsh OR (North* Irish)	Adult* Wom?n Men people

For peer review only - http://bmjopen.bmj.com/site/about/guidelines.xhtml

For peer review only

Community-centred interventions for improving public mental health among adults from minority ethnic populations in the United Kingdom: a scoping review

Journal:	*BMJ Open*
Manuscript ID	bmjopen-2020-041102.R1
Article Type:	Original research
Date Submitted by the Author:	13-Jan-2021
Complete List of Authors:	Baskin, Cleo; Imperial College London School of Public Health, Department of Primary Care and Public Health<br>Zijlstra, Geiske; Imperial College London School of Public Health, Department of Primary Care and Public Health<br>McGrath, Mike; UCL, Division of Psychiatry<br>Lee, C; University of Cambridge Institute of Public Health, Forvie Site<br>Duncan, Fiona; Durham University, Sport and Exercise Sciences<br>Oliver, Emily; Durham University, Department of Sport and Exercise Sciences<br>Osborn, David; UCL, Division of Psychiatry; Camden and Islington NHS Foundation Trust<br>Dykxhoorn, Jen; UCL, Division of Psychiatry; UCL, Department of Primary Care and Population Health<br>Kaner, Eileen; Newcastle University, Population Health Sciences Institute<br>LaFortune, Louise; University of Cambridge, Institute of Public Health<br>Walters, Kate; University College London, Department of Primary Care and Population Health<br>Kirkbride, James; University College London, Division of Psychiatry<br>Gnani, Shamini; Imperial College London School of Public Health, Department of Primary Care and Public Health
**Primary Subject Heading**:	Mental health
Secondary Subject Heading:	Health policy, Mental health, Public health
Keywords:	MENTAL HEALTH, PUBLIC HEALTH, Adult psychiatry < PSYCHIATRY

SCHOLARONE™ Manuscripts

I, the Submitting Author has the right to grant and does grant on behalf of all authors of the Work (as defined in the below author licence), an exclusive licence and/or a non-exclusive licence for contributions from authors who are: i) UK Crown employees; ii) where BMJ has agreed a CC-BY licence shall apply, and/or iii) in accordance with the terms applicable for US Federal Government officers or employees acting as part of their official duties; on a worldwide, perpetual, irrevocable, royalty-free basis to BMJ Publishing Group Ltd ("BMJ") its licensees and where the relevant Journal is co-owned by BMJ to the co-owners of the Journal, to publish the Work in this journal and any other BMJ products and to exploit all rights, as set out in our licence.

The Submitting Author accepts and understands that any supply made under these terms is made by BMJ to the Submitting Author unless you are acting as an employee on behalf of your employer or a postgraduate student of an affiliated institution which is paying any applicable article publishing charge ("APC") for Open Access articles. Where the Submitting Author wishes to make the Work available on an Open Access basis (and intends to pay the relevant APC), the terms of reuse of such Open Access shall be governed by a Creative Commons licence – details of these licences and which Creative Commons licence will apply to this Work are set out in our licence referred to above.

Other than as permitted in any relevant BMJ Author's Self Archiving Policies, I confirm this Work has not been accepted for publication elsewhere, is not being considered for publication elsewhere and does not duplicate material already published. I confirm all authors consent to publication of this Work and authorise the granting of this licence.

For peer review only

Community-centred interventions for improving public mental health among adults from minority ethnic populations in the United Kingdom: a scoping review

Zijlstra G[1*], Baskin C[1*], McGrath M [3], Lee C[6], Duncan F[2], Oliver E. J.[2], Osborn D[3,7], Dykxhoorn J[3,4], Kaner E[5], Lafortune L[6], Walters K[4], Kirkbride JB[3], Gnani S[1]

*Cleo Baskin and Geiske Zijlstra contributed equally to this paper.

1 Department of Primary Care and Public Health, School of Public Health, Imperial College London, Reynolds Building, St Dunstan's Road, London, W6 8RP.

2 Department of Sport and Exercise Sciences, Durham University, 42 Old Elvet, Durham, DH1 3HN,

3 Division of Psychiatry, UCL, Maple House, 149 Tottenham Court Road, London, W1T 7NF.

4 Department of Primary Care and Population Health, UCL Royal Free Campus, Rowland Hill Street, London NW3 2PF

5 Population Health Sciences Institute, Baddiley-Clark Building, Newcastle upon Tyne, NE2 4AX.

6 Cambridge Institute of Public Health, Forvie Site, Robinson Way, Cambridge, CB2 0SR.

7 Camden and Islington NHS Foundation Trust, NW1OPE

Corresponding author: Cleo Baskin

Department of Primary Care and Public Health, Imperial College London

Reynolds Building, St Dunstan's Road, London, W6 8RP

Phone: +447366416304

Email: cleo.baskin18@ic.ac.uk

Word count: 4000

For peer review only

Abstract

Objectives: Undertake a scoping review to determine the effectiveness of community-centred interventions designed to improve the mental health and well-being of adults from Black, Asian and Minority Ethnic (BAME) populations in the United Kingdom.

Methods: We searched six electronic academic databases for studies published between January 1990-September 2019: Medline, Embase, PsychINFO, Scopus, CINAHL and Cochrane. We used the Preferred Reporting Items for Systematic Reviews and Meta-Analyses (PRISMA) extension for Scoping Reviews checklist and Template for Intervention description and replication (TIDieR) guide for intervention description and data extraction. Quality was assessed using Cochrane Risk of Bias tools. Grey literature results were deemed beyond the scope of this review; preliminary searches found numerous (>50) small scale community interventions without available outcomes data.

Results: Of 4,501 studies, seven met the eligibility criteria of UK-based community interventions targeting mental health in BAME adults: four randomised control trials (RCT), one pre- and post-pilot study, one cross-sectional study and one ethnographic study. Interventions included therapy-style sessions, peer-support groups, educational materials, gym access, and a family services programme. Common components included a focus on tackling social isolation, using lay health workers from within the community, signposting, and cultural adaptation. Only three studies reported a statistically significant positive outcome on mental health outcomes and six were appraised as having a high risk of bias. Studies included mostly Indian and Pakistani ethnic groups. Knowledge gaps

For peer review only - http://bmjopen.bmj.com/site/about/guidelines.xhtml

emerged around effective interventions for men, populations such as Chinese, Arab and Travellers, and tackling the wider determinants of mental health.

Conclusions: There is a paucity of high-quality evidence regarding community-based interventions focussed on improving public mental health among BAME groups. Decision makers need scientific evidence to inform effective approaches to mitigating health disparities. Our next steps are to map promising community activities and interventions that are currently being provided to help identify emerging evidence.

Keywords

Mental health, well-being, Black, Asian and Minority Ethnic groups, minority groups, ethnicity, community-based

Strengths and limitations of this study

- This scoping review addresses the large evidence gap which exists on the effectiveness of community-centred interventions in improving public mental health of UK Black, Asian and Minority Ethnic (BAME) populations, who are disproportionately affected by poor mental health.
- A six-stage scoping review framework, including stakeholder consultation, was applied to summarise the evidence for community-centred interventions.
- We included all community interventions irrespective of setting and type.
- Analysis of review data were limited to studies published in peer-reviewed journals and citation tracking of references.
- We used national definitions for BAME categories but acknowledge challenges in viewing ethnic minority groups as homogenous in their mental health needs.

For peer review only - http://bmjopen.bmj.com/site/about/guidelines.xhtml

Introduction

Poor mental well-being disproportionately affects people from minority ethnic populations in the UK(1-8). Bias, racism and discrimination have had implications on many of the societal, structural and institutional risk factors for poor mental health. These include worse employment(9), housing conditions(10), and healthcare(11) and an increased likelihood to enter the criminal justice system(12) and live in poverty(13). Stigma toward mental illness is also reported to be higher among minority ethnic groups(14).

These risks affect mental health through numerous pathways, for example stigma and the lack of racially, culturally and/or ethnically appropriate healthcare may cause delays in help-seeking, ultimately leading to poorer outcomes(15,16). Additionally, stress- both acute and chronic- can affect physical health and mental wellbeing. The COVID-19 pandemic has exacerbated these risks, and has had a disproportionate impact on ethnic minorities in the UK(17).

In the UK, the term Black, Asian, and Minority Ethnic(BAME) is frequently used to refer to individuals who are in a racial or ethnic minority(18). This term has been criticised as grouping together a heterogenous population with diverse health needs; evidence indicates differential and complex risks for poor mental health(19,20). For example, the risk of psychosis among the Black Caribbean population is nearly seven times higher than the White British population(21), but not for Asian groups(22).

Strengthening public mental health involves both the promotion of mental health and wellbeing and the prevention of mental illness. It has the potential to effectively and

For peer review only

sustainably reduce social and racial inequalities in mental health outcomes(23,24). In pursuit of this, many UK initiatives have been implemented, such as the 2005 ‘Delivering Race Equality in Mental Healthcare Action Plan’(25), nevertheless, they have failed to successfully narrow the observed mental health gap(26,27).

Community-centred interventions (i.e., those that take place in a community setting, or are delivered by the community and/or voluntary sector) are increasingly recognised to have potential to influence the cultural and social factors that protect and promote mental health and wellbeing. These include social connectedness, access to safe and affordable housing, and power in local decision making(28,29). Although the National Health Service (NHS) England in 2017 introduced local financial incentives to improve mental health outcomes for BAME groups(30), local decision making has been hindered by a lack of evidence. The aim of this scoping review was to summarise the evidence for community-centred interventions focussed on improving the public mental health of BAME groups in the UK.

For peer review only

For peer review only - http://bmjopen.bmj.com/site/about/guidelines.xhtml

Methods

We undertook a scoping review, an approach for summarizing evidence and identifying knowledge gaps in unclear and emerging fields(31,32), using Arksey and O'Malley's six-stage methodological framework(33). The optional sixth stage of stakeholder consultation was undertaken by involving stakeholders and peer researchers as part of a wider research programme(34). Scoping reviews were not eligible for prospective registration with the international prospective register for systematic reviews (PROSPERO) at the time of conducting the review(35).

I) Study identification

A systematic search of six electronic databases between January 1990 and September 2019 was conducted: Medline, Embase, PsycINFO, Scopus, CINAHL and Cochrane. The search strategy (Appendix 1) was created with the support of a medical librarian and was based on the PICOC (population; intervention; comparison; outcomes; context) framework. It included key terms for ethnicity, age range, geography and mental health outcomes, but no intervention and comparison terms were included to optimise capture of all relevant studies.

We excluded evidence from the grey literature, as an initial search of primary data from local government, relevant third sector and NHS websites identified numerous activities and possible interventions (over 50 in a small geographical area), but outcomes data were not publicly available or were limited. Consequently, data synthesis exceeded the methodological approach of a scoping review; a mapping methodology would be more appropriate(36).

For peer review only

Eligibility criteria

Table 1 outlines the inclusion and exclusion criteria used to determine the eligibility of a study. We included studies published 1990 onwards so that our findings would inform contemporary policy and practice. Furthermore, systematic ethnicity data collection in the UK began with the 1991 Census(37). We included only UK studies due to variation in how race, ethnicity and ancestry are represented in different countries. This is a consequence of each countries' unique pattern of migration and its political and social context.

We included studies where participants were working aged adults (aged 18-64 years adapted from the UK definition of 16-64 years) who were either well or had a common mental disorder, such as anxiety and depression. Common mental disorders were included to reflect the high prevalence of these conditions in the general population, one in six adults in England(38). They often go undiagnosed and so are more amenable to interventions focused on prevention and the promotion of positive mental health(39). Studies focusing on people with severe mental illness (including suicide and psychotic disorders), or people affected by young onset dementia, were not included due to the need for specialist mental health treatment and tailored support. Studies that specifically targeted new migrants and refugee groups were also excluded due to the specific and complex mental health needs of this population, such as the high prevalence of post-traumatic stress disorder (40,41). Studies were included if new migrants or refugees happened to be recruited as participants but not if the study's intervention was specifically designed for them.

For peer review only - http://bmjopen.bmj.com/site/about/guidelines.xhtml

Only community-centred interventions were eligible for inclusion. We defined community-centred broadly to include all interventions that were not clinical in nature, that is, did not involve the treatment of patients by health professionals such as cognitive behavioural therapy and drug therapy(42,43). Therapy-style sessions that were delivered by lay health workers were included. There is a wide range of overlapping concepts in community- based and community- centred terms, and these were captured by our broad search strategy.

Table 1. Eligibility criteria

Inclusion criteria	Exclusion criteria
Adults aged 18 – 64 years	Children aged 0 -17 or adults aged over 64 years
Individuals from Asian/ Asian British, Black/ African/ Caribbean/ Black British, Mixed/Multiple ethnic groups and Arab ethnic groups	All White British ethnicities other than Gypsy or Irish Traveller, refugees and asylum seekers
Adults with no known mental disorder or diagnosed with a common mental disorder, such as anxiety and depression	Adults with severe mental illness and dementia
Community-centred interventions, i.e., take place in a community setting, or in a health setting but delivered by the community and/or voluntary sector	Clinical interventions that involved treatment by a clinician in any setting
Study measures any domain or aspect of mental health and well-being	No outcome measures for mental health or well-being
All studies based on primary research	Abstracts, posters, books or chapters, editorials and letters
Study conducted in the UK	Study conducted outside of the UK
Published from 1990 onwards	Published before 1990
Full-text research article available	No full text research article available

The UK Office of National Statistics 2011 Census ethnicity terms were used (Table 2)(44). Ethnic groups were deemed a minority if they fell under ‘Mixed’, ‘Asian’, ‘Black’

For peer review only - http://bmjopen.bmj.com/site/about/guidelines.xhtml

and 'Other' categories. All ethnic groups under the 'White' category were excluded, except for the 'Gypsy or Irish traveller' group due to their comparatively higher social and economic disadvantage and observed health inequalities(8).

Table 2. Ethnicity as Classified by the Office for National Statistics

**White**	**English / Welsh / Scottish / Northern Irish / British**
	Irish
	Gypsy or Irish Traveller
	Any other White background
**Mixed / Multiple ethnic groups**	White and Black Caribbean
	White and Black African
	White and Asian
	Any other Mixed / Multiple ethnic background
**Asian / Asian British**	Indian
	Pakistani
	Bangladeshi
	Chinese
	Any other Asian background
**Black / African / Caribbean / Black British**	African
	Caribbean
	Any other Black / African / Caribbean background
**Other ethnic group**	Arab
	Any other ethnic group

II) Study selection

Two independent reviewers(GZ,CB) screened non-duplicate titles and abstracts against eligibility criteria (n=4501). The abstracts that matched criteria (Table 1) were reviewed in full be GZ and CB (n=45). All conflicts were resolved by a third reviewer (SG); this was needed for 31% (n=14) of full text articles. Full texts that were not freely available were accessed via the British Library. Additional articles were identified by examining references and forward-backward citation searching (Figure 1). Covidence systematic review software(45) was used to remove duplicates and screen titles.

III) Charting of data

Data were extracted by two independent reviewers(GZ,CB). A data extraction framework was developed to capture key study characteristics: author; year of

For peer review only - http://bmjopen.bmj.com/site/about/guidelines.xhtml

publication; setting; study design; population demographics; sample size; theory of change; and outcomes and results. Intervention details were extracted using the Template for Intervention Description and Replication(TIDieR) guide: rationale; materials; procedures; provider; methods; location; timing; tailoring; modifications; and assessment of intervention adherence(46).

IV) Collation and summary of results

Quality appraisal was performed by two reviewers(GZ,CB) using the Cochrane tool for risk of bias for RCTs(47). Qualitative studies were appraised using the Cochrane Qualitative and Implementation Methods Group guidance and the Mixed-Method Appraisal Tool(MMAT) for mixed methods studies(48,49). A narrative data synthesis of the papers was conducted by identifying themes and mechanisms common to the community-based interventions. Review findings were reported using The Preferred Reporting Items for Systematic Reviews and Meta-Analyses (PRISMA) extension for Scoping Reviews checklist(50).

V) Patient and Public Involvement

The research question was informed by peoples' experiences and stakeholder workshops. This study did not involve the recruitment of patients, and no patients were involved in the design, or conduct of the study.

For peer review only

For peer review only - http://bmjopen.bmj.com/site/about/guidelines.xhtml

Results

We identified a total of 4,483 non-duplicate titles and an additional 18 from forward-backward citation searching (Figure 1). Seven out of the 45 studies reviewed in full-text matched the eligibility criteria and were included in this review: four RCTs (including one pilot); one pre- and post-intervention pilot study; one cross sectional; and one ethnographic study (Table 3).

Intervention type and focus

The interventions were highly heterogenous in design and focus. They included therapy-style sessions delivered by lay health workers(51,52), peer-support groups(53,54), an educational leaflet(55), free gym access(56), and a family services programme(57). All interventions were conducted in England and the most frequently targeted BAME population groups were of Indian and Pakistani heritage. There was a specific focus of interventions for women (n=4) and people with anxiety or depression (n=5).

Interventions were either single sessions or took place over a minimum of 10-weeks to eight months (the duration of the family services programme is unclear). Most aimed to improve mental health by expanding social networks and facilitating social support(51,53,54,57). Three studies aimed to increase access to services by facilitating linkages or signposting to complementary social care and mental health services (51,52,57). Qualitative data commonly found a reduction in social isolation and stress, and improved mood and self-confidence(52,54,56,57). One RCT reported significant improvement in social functioning(53) and two had a statistically significant positive impact on depression outcomes(54,55). All but one study(53) were appraised

as having a high risk of bias with limitations relating to small sample sizes (n=9-123, mean=43, median=30), short follow-up periods (ranging from two to nine months), and participant recruitment.

For peer review only

For peer review only - http://bmjopen.bmj.com/site/about/guidelines.xhtml

Table 3. Summary of community-based intervention studies targeted at Black, Asian and Minority Ethnic groups to improve mental health and well-being (N=7)

Author; year	Study design	Participant characteristics	Theory of Change	Intervention description	Results
**Jacob et al; 2002**	RCT	Asian women with depression in Ealing, London	Educate patients on depression to positively affect their perspective and outcomes of depression	Participants (n=70) received an educational leaflet about depression or usual care	**GHQ-12**: recovery from common mental disorder (defined as $\leq$ 2): OR 3.4 (95% CI: 1.01 to 11.5) at 2 months
**Gater et al; 2010**	RCT	British Pakistani women with depression in Northwest England	Improve mental health through reducing social isolation	Participants (n=123) received a social group intervention or antidepressant medication, or both	**HRSD** (depression): no significant effects<br><br>**Social functioning**: Significant effect at 3 months for social intervention vs. antidepressants, 6.1 (95% CI: 1.4 to 10.8), p= 0.02, and combined group vs. antidepressants, 5.9 (95% CI: 1.5 to 10.2), p= 0.017. No significant effect at 9 months<br><br>**Satisfaction**: Significant effects at 3 and 9 months for social vs. antidepressants, 4.9 (95% CI: 2.9 to 7.0), p=0.002; 4.7 (95% CI: 2.6 to 6.7), 0.002 and combined v. antidepressants, 4.3 (95% CI: 2.6 to 6.0), p=0.001; 4.0 (95% CI: 1.8 to 6.2), p=0.005<br><br>Qualitative feedback found a major hindrance to participation was resistance from family members. Participants engaged in the trial because of the culturally appropriate format and content of the sessions. They described their experience as 'relief from worries', 'feeling fresh' and 'better than their expectations'.
**Afuwape et al; 2010**	RCT	Black adults with anxiety and/or depression in Southwark, London	Improve psychosocial functioning and feelings of hope through a culturally acceptable care package	Participants (n=40) received individual therapy and group sessions on advice on services, health education and mentoring	**GHQ-29**: Significant effect at 3 months: adjusted mean difference 7.76 (95% CI: 0.86 to 14.65), p = 0.03<br><br>**GAF**: no significant effect<br><br>**SF-36**: mental health components had significant effect, −11.93 (95% CI: −21.99 to −1.88), p=0.02.<br>No effect on physical health. No significant differences in cost

For peer review only - http://bmjopen.bmj.com/site/about/guidelines.xhtml

**Lovell et al; 2014**	Pilot RCT	South Asian and Somalian women with moderate depression and/or anxiety in Northwest England	Improve treatment of depression and anxiety using social and community interventions	Participants (n=20) received individual or group therapy-style sessions, or usual care. Activities focussed on health and wellbeing; sign posting to service and education opportunities	**CORE-OM:** no effect<br><br>Small non-significant improvements in depression (PHQ9), health related quality of life (EQ5D) and functioning (WSAS) at 5 months<br><br>Qualitative data suggested that patients found the intervention acceptable, both in terms of content and delivery.
**Chaudhry et al; 2009**	Observational: Pre- and post-intervention	British Pakistani women diagnosed with depression In Manchester	Informal social support and mental and physical health education to reduce depression	Participants (n=9) received 10 weekly group sessions in various locations: psycho education, personal grooming, exercise and yoga	**SRQ**: reduction in depression scores pre- 15 (SD = 3.08) to post - 11.7 (SD = 5.95), p = 0.039.<br>SCAN interviews post-intervention diagnosed 2 participants as no longer depressed<br><br>**SCAN:** interviews post-intervention diagnosed 2 participants as no longer depressed<br><br>Anecdotal feedback from the participants identified that the relationships developed between the participants and facilitators and the provision of transport were the most important components of the intervention.
**Rabiee et al; 2015**	Cross-sectional; mixed methods	Deprived constituency in Birmingham	Regular exercise can help improve mood, self-esteem, confidence and quality of life	Gym for Free pilot project providing adults free access to leisure centres	Results indicate increased energy levels, confidence, mental well-being, reduction in stress and anxiety improved stress relief, and anger management
**Gray; 2003**	Ethnographic investigation	BAME families in Tower Hamlets, London	Reducing social isolation and poverty by improving parenting skills, self-esteem and meeting a welfare advisor	Personalised family support workers; trained volunteers of similar ethnic and cultural backgrounds	Family case records and interviews suggest close relationships were formed with the support workers of the same ethnic and cultural identity. They also portray a reduction in social isolation, bullying and racism Increased advocacy with other professionals and access to relevant services

For peer review only - http://bmjopen.bmj.com/site/about/guidelines.xhtml

Abbreviations: GHQ-12, General Health Questionnaire-12; HRSD, Hamilton Rating Scale for Depression; GHQ-29, General Health Questionnaire 29; GAF, Global assessment of Functioning; SF-36, Short form survey 36; CORE-OM, Clinical Outcomes in Routine evaluation- outcome measure scale; SRQ, Self-Reporting Questionnaire; SCAN, Schedule for clinical assessment in Neuropsychiatry

For peer review only

Outcome measures

Mental health outcomes were predominantly measured using standardised screening instruments for common mental disorders and social functioning (Table 4). Qualitative data were predominantly collected through focus groups and semi-structured interviews(52-54,56,57). Only one study reported on costs, calculated by measuring the difference in use of health and social services between the intervention and control group(51).

Table 4. Mental health and wellbeing outcome measures reported in the included studies (n = 7)

Author; year	*Quantitative measures*	*Qualitative outcomes*
Jacob et al; 2002	• **GHQ-12:** General Health Questionnaire 12<br>• **Short Explanatory Model Interview (SEMI):** self-reported perspective on depression	None
Gater et al; 2010	• **HRSD:** Hamilton Rating Scale for Depression<br>• **Verona Service Satisfaction Scale** (adapted)<br>• **Social functioning** (measured using a tool specifically created for British Pakistani women)	Feedback forms and focus groups
Afuwape et al; 2010	• **GHQ-28:** General Health Questionnaire 28<br>• **GAF:** Global assessment of Functioning<br>• **SF-36:** Short form survey (measuring quality of life)<br>• **LEDS:** Life Events and Difficulties Schedule	None
Lovell et al; 2014	• **PHQ-9:** Patient Health Questionnaire (measuring depression<br>• **COREOM:** Clinical outcomes in routine evaluation – outcome measure scale (measuring global distress)<br>• **GAD-7:** Generalised Anxiety Disorder Assessment<br>• **WSAS:** Work and Social Adjustment Scale<br>• **EQ-5D** (measuring health related quality of life)	Semi-structured interviews regarding the acceptability of the intervention
Chaudhry et al; 2009	• **SRQ:** Self Reporting Questionnaire (Urdu version; screening for mental disorders)<br>• **SCAN:** The Schedule for Clinical Assessment in Neuropsychiatry	Anecdotal feedback collected by facilitators
Rabiee et al; 2015	• Self-completed questionnaire evaluating experience in accessing services and perceived changes in health and well-being	Focus groups analysed using established frameworks and guidelines (58,59)
Gray; 2003	None	Family case records and interviews analysed using established frameworks (60,61)

For peer review only - http://bmjopen.bmj.com/site/about/guidelines.xhtml

Addressing social isolation and loneliness

Chaudhry et al.(54) evaluated the efficacy and cultural acceptability of establishing a social group for British Pakistani women with depression. Women were diagnosed with depression using the Schedule for Clinical Assessment in Neuropsychiatry (SCAN) and were recruited from an ongoing population-based study. Participants attended at least six of ten weekly meetings at a community centre for the Pakistani community. Meetings included one session of psychoeducation and self-selected activities including personal grooming, exercise, yoga, and visits to museums and local shopping malls.

The intervention was successful in improving mental health outcomes; a significant reduction in depressive symptoms was found at the end of the 10 sessions (SRQ scores pre-intervention:15, SD=3.08; post-intervention:11.7, SD= 5.95, p=0.039) and three women reported a reduction in suicidal ideas. These outcomes, and participants' anecdotal feedback, suggest that a reduction in depressive symptoms is partly attributed to reducing social isolation by bringing the women together. Participants highly valued the provision of free transport by female Urdu speakers to avoid any objections from family and community members. Heterogeneity in the content and attendance of each session makes it difficult to assess the mechanism for improved outcomes. The small sample size and lack of control group further limit the generalisability of these findings.

Chaudhry's pilot study formed the basis of the RCT conducted by Gater et al.(53). British Pakistani women (n=123) with depression were recruited from general practice surgeries. The women were randomised into one of three intervention arms: a social

For peer review only - http://bmjopen.bmj.com/site/about/guidelines.xhtml

group intervention, antidepressant medication, or both the social group intervention and antidepressant medication. The social group involved ten women, who attended ten weekly sessions of social activities. These took place at a community centre and were facilitated by Urdu-speaking women who had completed a five-day training programme.

The intervention had mixed effects on mental health outcomes. There was no significant change in depression in all three arms. Social functioning significantly improved in both the social intervention and combined treatment groups at three months (6.1, 95% CI: 1.4,10.8 and 5.9, 95% CI:1.5,10.2). However, this effect did not persist at 9 months. Focus groups, held at the end of each session, found that the intervention was too short and that a major barrier to participating was resistance from family members. Participants had found it helpful to confide in others and were intending to maintain new friendships. Overall, this study was appraised as low risk and provides evidence in favour of social group interventions for short-term improvements of positive mental health.

Promoting access and use of services

Gray(57) evaluates a Family Support Service which provides home-based support and parenting lessons for families; support for parents with a severe mental illness; and help from support workers and the local authority in child protection. The service was delivered by Family Support Workers who were recruited from the local community or had similar backgrounds to the client group.

For peer review only - http://bmjopen.bmj.com/site/about/guidelines.xhtml

Thirty case records of families were examined, and 22 interviews were conducted with support workers, families themselves, and professionals managing or referring to these services. Support workers were found to have had a positive impact on families by bringing them together; reducing social isolation, bullying and racism; promoting equality of access and pertinent use of services; and liaising successfully with other health and social care services. It was highly valued that projects were culturally sensitive and that support workers were of the same ethnicity. Power dynamics were important as facilitators' decisions were of potentially significant consequence to the families. This makes the support workers not only essential to delivering the intervention but also integral to achieving a positive outcome for families.

Jacob et al.(55) evaluated the effect of a culturally appropriate educational leaflet on depression delivered in general practice to women of Indian, Bangladeshi and Pakistani heritage (n=70). Only women diagnosed with common mental disorder through General health Questionnaire (GHQ) were included in the study. Participants first had their perspective (explanatory models) on depression assessed using the Short Explanatory Model Interview (SEMI). They were then randomly allocated to receive either a leaflet about the nature, causes, prevalence and treatment of depression or to usual care from their general practitioner. The leaflet was available in English, Hindi and Punjabi and read out to illiterate participants. After two months all participants repeated the GHQ and re-discussed the SEMI, particularly focusing on the idea of depression as an illness and whether medical help is necessary.

There was no statistically significant difference between the intervention and control group on the explanatory model of depression at follow-up. The number of women

For peer review only - http://bmjopen.bmj.com/site/about/guidelines.xhtml

who recovered from depression (defined as GHQ-12 ≤ 2) at two months was significantly associated with receiving the educational leaflet (42.9% vs. 20% for controls, $p<0.05$; no median score of confidence intervals were reported). However, women who were in the intervention group had significantly lower baseline levels of psychiatric morbidity at entry into the trial. As such, this selection bias may explain the observed differences between the two groups.

Rabiee et al.(56) conducted a survey questionnaire(n=257) and focus groups to evaluate the physical and mental health impact of a 'Gym for Free' scheme in four leisure centres. The survey was intended to elicit participants' experiences of accessing the service as well as information about their height and weight and perceived changes in their general health and wellbeing. Benefits of the scheme included improved confidence(n=92) and energy(n=121), reduced stress(n=77), and improved anger management(n=8).

Only nine participants participated in three focus groups: two men (one White and one Black) and seven women (four Pakistani, two Indian and one White). Participants valued building their social networks through the scheme and they acknowledged the links between their physical and mental well-being. Although the authors suggest a financial barrier inhibiting people joining the gym, no formal data were collected on cost or participant finances. The generalisability of these findings is limited as participants were selected through opportunistic sampling and may over-represent younger adults who are motivated to use the gym.

For peer review only - http://bmjopen.bmj.com/site/about/guidelines.xhtml

Lay health workers delivering complex interventions

Afuwape et al.(51) evaluated the London Cares of Life Project(CoLP) which is a complex social intervention designed to improve the mental health of adults of black African or black Caribbean origin(n=40). Participants with anxiety and/or depression, diagnosed using the World Health Organisation checklist criteria, were randomised to receive the intervention immediately or to a three-month waiting list control and information on local mental health services. During the three-month intervention, community health workers (psychology graduates trained to deliver the intervention) provided: practical advice to address social needs; health education; and gave brief therapies based on cognitive behavioural therapy principles.

Those who received the intervention showed significant improvement at 3-months in depressive symptoms (adjusted mean difference=7.76, CI 95%: 0.86 to 14.65). However, there was no evidence of improved general functioning (adjusted mean difference= -0.78, CI 95%: -10.40 to 8.84). The effect of the intervention beyond three months in unknown. There was no difference in costings between the intervention and control group; health and social care utilisation costs were calculated for three months. The risk of bias is increased by the small sample size and baseline characteristics; participants in the non-intervention arm had a significantly greater prevalence of past psychiatric history. The generalisability to the wider BAME population is potentially limited due to the exclusion of non-English speaking participants and the use of screening tools that do not have clear evidence of validity in these populations.

Lovell et al.(52) examined the effect of a wellbeing intervention, based on cognitive behavioural strategies, aimed at decreasing anxiety, depression and social isolation

For peer review only

in both BAME and elderly populations. Only the results from the BAME group were included in this analysis (aged 21-58 years, n=20). Participants chose between receiving the intervention either individually, as part of a group, or as signposting sessions. Interventions in all three arms were delivered by wellbeing facilitators, after a three-day training programme, who liaised with the participants' health and social care professionals. Individual interventions comprised eight 30-minute sessions over 16 weeks, groups were delivered 8-10 weekly two-hour sessions, and those who participated in signposting sessions were seen three times over a 16-week period.

This intervention had no statistically significant effect on mental health outcomes. The semi-structured interviews established that acceptability was enhanced by involving family and the community, having a health professional's recommendation, and having an empathetic facilitator. This study is limited by its small sample size. Additionally, the element of patients choosing their intervention arm complicates the analysis of the mechanism of effect, albeit reflecting the realities of engagement and participation when delivering interventions.

For peer review only

For peer review only - http://bmjopen.bmj.com/site/about/guidelines.xhtml

Discussion

Main findings

We found seven studies of community-centred interventions that aimed to improve mental health and wellbeing among BAME groups in the UK. Although studies varied in design, four key interventional characteristics emerged.

First, interventions aimed to address social isolation through building peer-to-peer support and social networks(53,54,56). Evidence suggests BAME populations find it easier to start conversations on mental health within their own cultural networks rather than with health professionals; formal mental health services perceived as a last resort(62,63). We found that social support interventions mainly targeted women and it is unclear whether men would find these acceptable.

Second, interventions aimed to overcome cultural and other barriers in accessing care. For example, by translating educational materials(55), addressing practical considerations, such as travel(53,54) and using facilitators that shared the same ethnicity and/or life experience as participants(53-55,57). This concurs with emerging evidence that culturally adapted interventions lead to better outcomes among minority ethnic groups(64,65).

Third, interventions were delivered by lay health workers(51,52,57). Over recent years, task-shifting in healthcare from health professionals to lay health workers has been common due to the need to meet escalating demand for care(66). Lay health workers from the same community as patients may be perceived as more accessible(67) and help reduce the associated stigma of accessing mental health

For peer review only - http://bmjopen.bmj.com/site/about/guidelines.xhtml

services(68,69). They may also overcome language and cultural barriers that minority ethnic groups face when trying to communicate mental health needs to their healthcare provider(70,71). However, lay health workers, unless they have received anti-stigma training, may hold beliefs that are more stigmatising than those held by health professionals(72).

Four, interventions had an emphasis on signposting and or facilitating linkages to complementary, or additional services. Signposting has been widely adopted in England through social prescribing schemes, but to date there has been little emphasis on cultural appropriateness(73). Evidence suggests that mental health services provided by the voluntary and community sector and embedded in communities increases trust among BAME communities, which in turn promotes awareness of mental health problems and access to mental health services(2).

We found community interventions focussed on specific groups, mainly South Asian and Afro-Caribbean populations. There were no interventions designed for and targeting men, despite men being less likely to seek help for common mental health problems(74).

Limitations

A limitation of this study was the omission of grey literature. Preliminary grey literature searching found over 50 community-centred services, which confirms that most relevant interventions reside outside publication in peer review journals and indicates significant publication bias. We were unable to include these interventions in this review due to the limitation and availability of primary data. Furthermore, we consider

For peer review only

a mapping methodology to be more appropriate to comprehensively summarise this evidence(36). We also only included community-centred interventions that targeted BAME groups. It is possible that BAME groups access public mental health interventions through universal provision, yet uptake of screening programmes and utilisation of mental health care services shows BAME populations have lower use(75).

A further limitation is the generalisability of our results that include all BAME populations in the UK; homogenous recommendations cannot be made to a culturally and ethnically heterogenous population. Furthermore, we excluded all white ethnic minority groups apart from Irish Travellers. However, the UK has a white ethnic minority European population, such as people from Poland, who may be subject to similar systemic health inequalities and challenges to their mental health and well-being as other BAME groups(2).

Interventions specifically targeting refugees, asylum seekers and new migrants were also excluded. In the UK, for example, refugee's makeup approximately 0.2%(76) of the population and new migrants 1.1%(77) and research indicates that these groups are more likely to require specialised clinical interventions focussed on reducing psychological trauma(78,79). However, in light of the overall paucity of evidence in this field and the likelihood of shared need, future research should consider whether this exclusion is necessary.

For peer review only - http://bmjopen.bmj.com/site/about/guidelines.xhtml

Implications for policy makers and future research

We recognise that the evidence for ethnic differences in some common mental disorders is complex, variable and indeed equivocal for specific ethnic groups. However, there is a disconnect between the scale of community-centred intervention provisions and published evidence. This may imply a bias in terms of scientific priorities and funding that needs urgent rectification, as current evidence is limited and weak.

It is important that future research seeks to understand *how* successful interventions work to improve mental health and for whom, taking into account intersectionality such as between gender and ethnicity. This requires resources to be appropriately allocated to third sector organisations, who typically provide these interventions, as they are often bound to deliver short-term projects and lack resources for robust evaluations. Evaluations should also include economic analysis; community interventions are potentially an affordable means to improving public mental health(80).

These findings also suggest the need for more activity on preventing mental illness and promoting wellbeing; most of the studies focussed on people with existing common mental conditions. Health promotion and primary prevention alongside universal approaches are critical components of strong public mental health and sustainable health systems(81). Additionally, further research is needed to understand the societal, structural and institutional challenges affecting community-centred public mental health interventions for BAME groups to help identify potential solutions.

For peer review only - http://bmjopen.bmj.com/site/about/guidelines.xhtml

Lastly, labels such as 'Asian' or 'Black African' present challenges to mental health research by viewing ethnic minority groups as homogenous in their mental health needs, despite evidence indicating otherwise(82-84). We recommend that national census categories are consistently used across all sectors, so that important variations and inequities can be identified and investigated further.

Conclusion

Despite well-documented ethnic disparities in mental health, there is a paucity of high-quality evidence regarding community-centred interventions that focus on improving public mental health among BAME groups. Decision makers need scientific evidence to help commission appropriate services and to inform effective approaches to mitigating these health disparities. Our next steps are to map the promising community activities and interventions that are currently being provided to help identify emerging evidence.

Data Sharing

Data sharing not applicable as no datasets generated and/or analysed for this study. This was a scoping review with no original individual participant data.

Figure legends

Figure 1. PRISMA flow chart of search strategy

Legend: The PRIMSA diagram details our search and selection process applied during the overview.

For peer review only - http://bmjopen.bmj.com/site/about/guidelines.xhtml

Acknowledgements

Many thanks to the library staff at Imperial College London for their insights in the design of search terms and their support in the use of resources.

Funding Statement

This study is funded by the National Institute for Health Research (NIHR) School for Public Health Research (SPHR) (Grant Reference Number PD-SPH-2015). The views expressed are those of the author(s) and not necessarily those of the NIHR or the Department of Health and Social Care.

The NIHR School for Public Health Research is a partnership between the Universities of Sheffield; Bristol; Cambridge; Imperial; and University College London; The London School for Hygiene and Tropical Medicine (LSHTM); LiLaC - a collaboration between the Universities of Liverpool and Lancaster; and Fuse - The Centre for Translational Research in Public Health a collaboration between Newcastle, Durham, Northumbria, Sunderland and Teesside Universities.

Competing Interests

The authors have no competing interests to declare.

Authors' Contributions

SG conceived the idea. GZ, CB and SG designed and conducted the study, which was supervised by SG. GZ and CB wrote the original draft of the manuscript. MM, CL, FD, EO, DO, JD, EK, LL, KW and JBK contributed to the interpretation of the results and

For peer review only

revised the manuscript critically for intellectual content and technical and language edits. Further reviewing and editing was undertaken by GZ, CB and SG.

References

1. Bhui KS, McKenzie K. Rates and Risk Factors by Ethnic Group for Suicides Within a Year of Contact With Mental Health Services in England and Wales. *Psychiatr Serv*. 2008 Apr;59(4):414–20.doi: 10.1176/ps.2008.59.4.414
2. Weich S, Nazroo J, Sporston K, et al. Common mental disorders and ethnicity in England: The EMPIRIC study. *Psychol Med*. 2004 Nov;34(8):1543–51.doi: 10.1017/s0033291704002715
3. Williams ED, Tillin T, Richards M, et al. Depressive symptoms are doubled in older British South Asian and Black Caribbean people compared with Europeans: Associations with excess co-morbidity and socioeconomic disadvantage. *Psychol Med*. 2015;45(9):1861–71.doi: 10.1017/S0033291714002967
4. Adkison-Bradley C, Maynard D, Johnson P, Carter S. British African Caribbean women and depression. *Br J Guid Couns*. 2009 Feb;37(1):65–72.doi: 10.1080/03069880802535887
5. Ngwena J. Black and minority ethnic groups (BME) suicide, admission with suicide or self-harm: An inner city study. *J Public Heal*. 2014;22(2):155–63.doi: 10.1007/s10389-013-0600-9
6. Karlsen S, Nazroo JY, McKenzie K, et al. Racism, psychosis and common mental disorder among ethnic minority groups in England. *Psychol Med*. 2005 Dec;35(12):1795–803.doi: 10.1017/S0033291705005830
7. Morgan C, Fearon P, Lappin J, et al. Ethnicity and long-term course and outcome of psychotic disorders in a UK sample: The ÆSOP-10 study. *Br J Psychiatry*. 2017 Aug 2;211(2):88–94.doi: 10.1192/bjp.bp.116.193342
8. Bignall T, Jeraj S, Helsby E, et al. Racial disparities in mental health: literature and evidence review — NHS Networks. 2020 [cited 2020 Mar 19]. Available

For peer review only - http://bmjopen.bmj.com/site/about/guidelines.xhtml

from: https://www.networks.nhs.uk/news/racial-disparities-in-mental-health-literature-and-evidence-review

9. Powell A, Francis-Devine B, Foley N. Unemployment by ethnic background. House of Commons Library. 2020.

10. “Overcrowded households,” Ethnicity facts and figures, September 9, 2020, service.gov.uk.

11. Memon A, Taylor K, Mohebati LM, et al. Perceived barriers to accessing mental health services among black and minority ethnic (BME) communities: A qualitative study in Southeast England. *BMJ Open*. 2016 Nov 1;6(11):e012337.

12. Uhrig N. Black, Asian and Minority Ethnic disproportionality in the Criminal Justice System in England and Wales. Ministry of Justice Analytical Services. 2016.

13. Barnard H, Turner C. Poverty and ethnicity: A review of evidence. York:JRF.

14. Mantovani N, Pizzolati M, Edge D. Exploring the relationship between stigma and help-seeking for mental illness in African-descended faith communities in the UK. *Heal Expect*. 2017 Jun;20(3):373–84.doi: 10.1111/hex.12464.

15. Abbey S, Charbonneau M, Tranulis C, Moss P, et al. Stigma and discrimination. Can J Psychiatry. 2012;56(10):1–9.

16. Henderson C, Noblett J, Parke H, et al. Mental health-related stigma in healthcare and mental health–care settings. Lancet Psychiatry. 2014;1(6):467–482.

17. Problems amid Progress: Improving Lives and Livlihood for ethnic minorities in the United Kingdom. October 2020, McKinsey.

For peer review only - http://bmjopen.bmj.com/site/about/guidelines.xhtml

18. Definitions | Institute of Race Relations. [cited 2020 May 6]. Available from: http://www.irr.org.uk/research/statistics/definitions/

19. Khunti K, Routen A, Pareek M, Treweek S, Platt L. The language of ethnicity. BMJ. 2020 Nov 23;371.

20. Bignall T, Jeraj S, Helsby E, et al. Racial disparities in mental health: literature and evidence review — NHS Networks. 2020 [cited 2020 Mar 19]. Available from: https://www.networks.nhs.uk/news/racial-disparities-in-mental-health-literature-and-evidence-review

21. Fearon P, Kirkbride JB, Morgan C, et al. Incidence of schizophrenia and other psychoses in ethnic minority groups: Results from the MRC AESOP Study. Psychol Med. 2006 Nov;36(11):1541–50.doi: 10.1017/S0033291706008774.

22. McKenzie K, Bhui K, Nanchahal K, et al. Suicide rates in people of South Asian origin in England and Wales: 1993-2003. *Br J Psychiatry*. 2008 Nov;193(5):406–9.doi: 10.1192/bjp.bp.107.042598.

23. Public mental health - GOV.UK [Internet]. [cited 2020 Mar 19]. Available from: https://www.gov.uk/government/collections/public-mental-health

24. Davies SC. Annual Report of the Chief Medical Officer: Public Mental Health Priorities: Investing in the Evidence. 2014.

25. Department of Health. Delivering race equality in mental health care, an Action Plan for reform inside and outside services; and the Government's response to the indpeendent inquiry into the death of David Bennett. London: 2005.

26. Care Quality Commission. Count me in 2010: Results of the 2010 national census of inpatients and patients on supervised community treatment in mental health and learning disability services in England and Wales. London: 2011.

27. Salway S, Holman D, Lee C, et al. Transforming the health system for the UK's multiethnic population. *BMJ*. 2020; 368:m268

28. Health matters: community-centred approaches for health and wellbeing - GOV.UK [Internet]. [cited 2020 Mar 16]. Available from: https://www.gov.uk/government/publications/health-matters-health-and-

For peer review only - http://bmjopen.bmj.com/site/about/guidelines.xhtml

wellbeing-community-centred-approaches/health-matters-community-centred-approaches-for-health-and-wellbeing

29. NICE. Impact Mental Health. NICE 2019.

30. Local Government Improvement and Development. Culturally responsive JSNAs: a review of race equality and Joint Strategic Needs Assessment (JSNA) practice. 2010.

31. Peters MDJ, Godfrey CM, Khalil H, et al. Guidance for conducting systematic scoping reviews. *Int J Evid Based Healthc*. 2015;13(3):141–6.doi: 10.1097/XEB.0000000000000005

32. Tricco AC, Lillie E, Zarin W, et al. A scoping review on the conduct and reporting of scoping reviews. *BMC Med Res Methodol.* 2016;16(1):1–10.doi: 10.1186/s12874-016-0116-4

33. Arksey H, O'Malley L. Scoping studies: Towards a methodological framework. *Int J Soc Res Methodol Theory Pract*. 2005 Feb;8(1):19–32.doi: 10.1080/1364557032000119616

34. Public mental health programme - NIHR School for Public Health Research NIHR SPHR [Internet]. [cited 2020 May 6]. Available from: https://sphr.nihr.ac.uk/category/research/public-mental-health/

35. Booth A, Clarke M, Dooley G, et al. The nuts and bolts of PROSPERO: An international prospective register of systematic reviews. *Syst Rev.* 2012 Feb 9;1;2.doi: 10.1186/2046-4053-1-2.

36. Duncan, F. H., McGrath, M., Baskin, C., Osborn, D., Dykxhoorn, J., Kaner, E. F., Oliver & Kirkbride, J. (2020). Delivery of community-centred public mental health interventions in diverse areas in England: a mapping study protocol. *BMJ open*, *10*(7), e037631.

37. 50 years of collecting ethnicity data - History of government [Internet]. [cited 2020 Apr 7]. Available from: https://history.blog.gov.uk/2019/03/07/50-years-of-collecting-ethnicity-data/

38. Adult Psychiatric Morbidity Survey: Survey of Mental Health and Wellbeing,

For peer review only

For peer review only - http://bmjopen.bmj.com/site/about/guidelines.xhtml

England, 2014. NHS Digital. [cited 2020 Mar 20]. Available from: https://webarchive.nationalarchives.gov.uk/20180328140249/http://digital.nhs.uk/catalogue/PUB21748

39. National Institute for Health and Care Excellence. Common mental health problems: identification and pathways to care. 2011.

40. Porter M, Haslam N. Predisplacement and postdisplacement of refugees and internally displaced persons. *J Am Med Assoc*. 2005;294(5):610–2.doi: 10.1001/jama.294.5.602

41. Steel Z, Chey T, Marnane C, et al. Association of Torture and Other Potentially Traumatic Events With Mental Health Outcomes Among Populations Exposed to Mass Conflict and Displacement: A Systematic Review and Meta-analysis. *J Am Med Assoc*. 2019;302(5).doi: 10.1001/jama.2009.1132.

42. Depression in adults: recognition and management - Clinical Guideline 90. 2009.

43. Generalised anxiety disorder and panic in adults: management - Clinical guideline 113. 2011.

44. National Office for Statistics. 2011 Census Analysis: Ethnicity and Religion of the Non-UK Born Population in England and Wales. 2015;(June):5.

45. Veritas Health Innovation. Covidence systematic review software. Veritas Health Innovation, Melbourne, Australia.

46. Hoffmann TC, Glasziou PP, Boutron I, Milne R, Perera R, Moher D, et al. Better reporting of interventions: Template for intervention description and replication (TIDieR) checklist and guide. *BMJ*. 2014 Mar;348:g1687

47. Higgins JP, Altman DG. Assessing Risk of Bias in Included Studies. In: Cochrane Handbook for Systematic Reviews of Interventions: Cochrane Book Series. 2008.

48. Noyes J, Booth A, Flemming K, et al. Cochrane Qualitative and Implementation Methods Group guidance series—paper 3: methods for assessing methodological limitations, data extraction and synthesis, and confidence in synthesized qualitative findings. *J Clin Epidemiol*. 2018 May;97:49–58.doi: 10.1016/j.jclinepi.2017.06.020. Epub 2017 Dec 13.

49. Pluye P, Gagnon MP, Griffiths F, et al. A scoring system for appraising mixed methods research, and concomitantly appraising qualitative, quantitative and

For peer review only - http://bmjopen.bmj.com/site/about/guidelines.xhtml

mixed methods primary studies in Mixed Studies Reviews. Int J Nurs Stud. 2009 Apr; 46 (4):529-46.doi: 10.1016/j.ijnurstu.2009.01.009.Epub 2009 Feb 23.

50. Tricco AC, Lillie E, Zarin W, et al. PRISMA extension for scoping reviews (PRISMA-ScR): Checklist and explanation. Vol. 169, Annals of Internal Medicine. American College of Physicians; 2018. p. 467–73.

51. Afuwape SA, Craig TKJ, Harris T, et al. The Cares of Life Project (CoLP): An exploratory randomised controlled trial of a community-based intervention for black people with common mental disorder. *J Affect Disord*. 2010;127(1–3):370–4.doi: 10.1016/j.jad.2010.05.017. Epub 2010 Jun 14.

52. Lovell K, Lamb J, Gask L, et al. Development and evaluation of culturally sensitive psychosocial interventions for under-served people in primary care. *BMC Psychiatry*. 2014 Aug 1;14:217.doi: 10.1186/s12888-014-0217-8.

53. Gater R, Waheed W, Husain N, Tomenson B, Aseem S, Creed F. Social intervention for British Pakistani women with depression: Randomised controlled trial. *Br J Psychiatry.* 2010;197(3):227–33.doi: 10.1192/bjp.bp.109.066845.

54. Chaudhry N, Waheed W, Husain N, et al. Development and pilot testing of a social intervention for depressed women of Pakistani family origin in the UK. *J Ment Heal.* 2009;18(6):504–9.doi: 10.3109/09638230902968209.

55. Jacob KS, Bhugra D, Mann AH. A Randomised Controlled Trial of an Educational Intervention for Depression Among Asian Women in Primary Care in the United Kingdom. *Int J Soc Psychiatry*. 2002 Jun;48(2):139-48.doi: 10.1177/002076402128783190.

56. Rabiee F, Robbins A, Khan M. Gym for Free: The short-term impact of an innovative public health policy on the health and wellbeing of residents in a deprived constituency in Birmingham, UK. *Health Educ J.* 2015;74(6):691–704.

57. Gray B. Social Exclusion, Poverty, Health and Social Care in Tower Hamlets: The Perspectives of Families on the Impact of the Family Support Service. *Br J Soc Work*. 2003;33(3):361–80.doi: 10.1093 bjsw33.3.361

58. Krueger R, Casey M. Focus groups: A practical guide for applied research. Thousand Oaks, CA: Sage Publications 2000.

59. Rabiee F. Focus-group interview and data analysis. *The Proceedings of the*

For peer review only - http://bmjopen.bmj.com/site/about/guidelines.xhtml

Nutrition Society. 2004, 63(4):655-660.doi: 10.1079/pns2004399

60. Garfinkel E, Cliffs H. Studies in ethnomethodology.1967.

61. Glaser BG, Strauss AL. Discovery of grounded theory: Strategies for qualitative research. Discovery of Grounded Theory: Strategies for Qualitative Research. Taylor and Francis; 1967. 1–271 p.

62. Tabassum R, Macaskill A, Ahmad I. Attitudes towards mental health in an urban Pakistani community in the United Kingdom. *Int J Soc Psychiatry*. 2000;46(3):170–81.doi: 10.1177/002076400004600303.

63. Sheikh S, Furnham A. A cross-cultural study of mental health beliefs and attitudes towards seeking professional help. *Soc Psychiatry Psychiatr Epidemiol*. 2000;35(7):326–34.doi: 10.1007/s001270050246.

64. Chowdhary N, Jotheeswaran AT, Nadkarni A, et al. The methods and outcomes of cultural adaptations of psychological treatments for depressive disorders: a systematic review. *Psychol Med.* 2014 Apr;44(6):1131-46.doi: 10.1017/S0033291713001785. Epub 2013 Jul 19.

65. Benish S, Quintana S, Wampold B. Culturally Adapted Psychotherapy and the Legitimacy of Myth: A Direct-Comparison Meta-Analysis. *J Couns Psychol*. 2011 Jul;58(3):279-89.doi: 10.1037/a0023626.

66. Shahmalak U, Blakemore A, Waheed MW, et al. The experiences of lay health workers trained in task-shifting psychological interventions: a qualitative systematic review. *Int J Ment Health Syst*. 2019;13:64.doi: 10.1186/s13033-019-0320-9.

67. Tabassum R, Macaskill A. Attitudes towards mental health in an urban Pakistani community in the United Kingdom. *Int J Soc Psychiatry*. 2000;46(3):170–81.doi: 10.1177/002076400004600303.

68. Li PL, Logan S, Yee L, et al. Barriers to meeting the mental health needs of the Chinese community. *J Public Health Med*. 1999 Mar;21(1):74–80.doi: 10.1093/pubmed/21.1.74.

For peer review only - http://bmjopen.bmj.com/site/about/guidelines.xhtml

69. Singla D, Lazarus A, Atif N, et al. “Someone like us”: Delivering maternal mental health through peers in two South Asian contexts. *J Affect Disord.*2014 Oct;168:452-8.doi: 10.1016/j.jad.2014.07.017.

70. McClay CA, Morrison J, McConnachie A, et al. A community-based group-guided self-help intervention for low mood and stress: Study protocol for a randomized controlled trial. *Trials.* 2013; 14:392.doi: 10.1186/1745-6215-14-392.

71. Mueller M, Kennerley H, McManus F et al. Oxford Guide to Surviving as a CBT Therapist. Oxford University Press 2010.

72. Iheanacho, T., Kapadia, D., Ezeanolue, C.O., Osuji, A.A., Ogidi, A.G., Ike, A., Patel, D., Stefanovics, E., Rosenheck, R., Obiefune, M. and Ezeanolue, E.E., 2016. Attitudes and beliefs about mental illness among church-based lay health workers: experience from a prevention of mother-to-child HIV transmission trial in Nigeria. *International Journal of Culture and Mental Health*, *9*(1), pp.1-13

73. Bickerdike L, Booth A, Wilson PM, et al. Social prescribing: Less rhetoric and more reality. A systematic review of the evidence. *BMJ Open*. 2017 Apr;7(4).doi:10.1136/bmjopen-2016-013384

74. Oliver MI, Pearson N, Coe N, et al. Help-seeking behaviour in men and women with common mental health problems: Cross-sectional study. *Br J Psychiatry*. 2005 Apr;186:297-301.doi: 10.1192/bjp.186.4.297.

75. Mcdaid D, Park A-L, Wahlbeck K. The Economic Case for the Prevention of Mental Illness. *Annu Rev Public Heal*. 2019 Apr 1;40:373–89.doi: 10.1146/annurev-publhealth-040617-013629. Epub 2019 Jan 2

76. Global Trends: Forced Displacement in 2019, 2019. UNHCR. [cited 2020 Dec

For peer review only

For peer review only - http://bmjopen.bmj.com/site/about/guidelines.xhtml

17] Available from: https://www.unhcr.org/5ee200e37.pdf

77. Migration Statistics Quarterly Report: August 2020, 2020. ONS. [cited 2020 Dec 17] Available from: https://www.ons.gov.uk/peoplepopulationandcommunity/populationandmigration/internationalmigration/bulletins/migrationstatisticsquarterlyreport/august2020

78. Byrow Y, Pajak R, McMahon T, et al. Barriers to mental health help-seeking amongst refugee men. *Int J Environ Res Public Health*. 2019 Aug 1;16(15):2634.doi: 10.3390/ijerph16152634.

79. Hynie M. The Social Determinants of Refugee Mental Health in the Post-Migration Context: A Critical Review. *Can J Psychiatry.* 2018 May;63(5):297-303.doi: 10.1177/0706743717746666.

80. McLaughlin KA. The Public Health Impact of Major Depression: A Call for Interdisciplinary Prevention Efforts, *Prevention Science*. 2011 Nov; 12(4): 361-371. doi: 10.1007/s11121-011-0231-8

81. Mcdaid D, Park A-L, Wahlbeck K. The Economic Case for the Prevention of Mental Illness. *Annu Rev Public Heal*. 2019 Apr 1;40:373–89.doi: 10.1146/annurev-publhealth-040617-013629. Epub 2019 Jan 2.

82. Care Quality Commission. Count me in: 2010 Results of the 2010 national census of inpatients and patients on supervised community treatment in mental health and learning disability services in England and Wales. London, UK: Care Quality Commission; 2011.

83. Care Quality Commission. Count Me In: Results of the 2009 National Census of Inpatients and Patients on Supervised Community Treatment Orders in Mental Health and Learning. London, UK: Care Qiuality Commission; 2010.

For peer review only - http://bmjopen.bmj.com/site/about/guidelines.xhtml

84. Weich S, Nazroo J, Sprostoin K, et al. Common mental disorders and ethnicity in England: the EMPIRIC Study. *Psychol Med.* 2004; 34(8):1543-1551.doi:10.1017/s0033291704002715

For peer review only

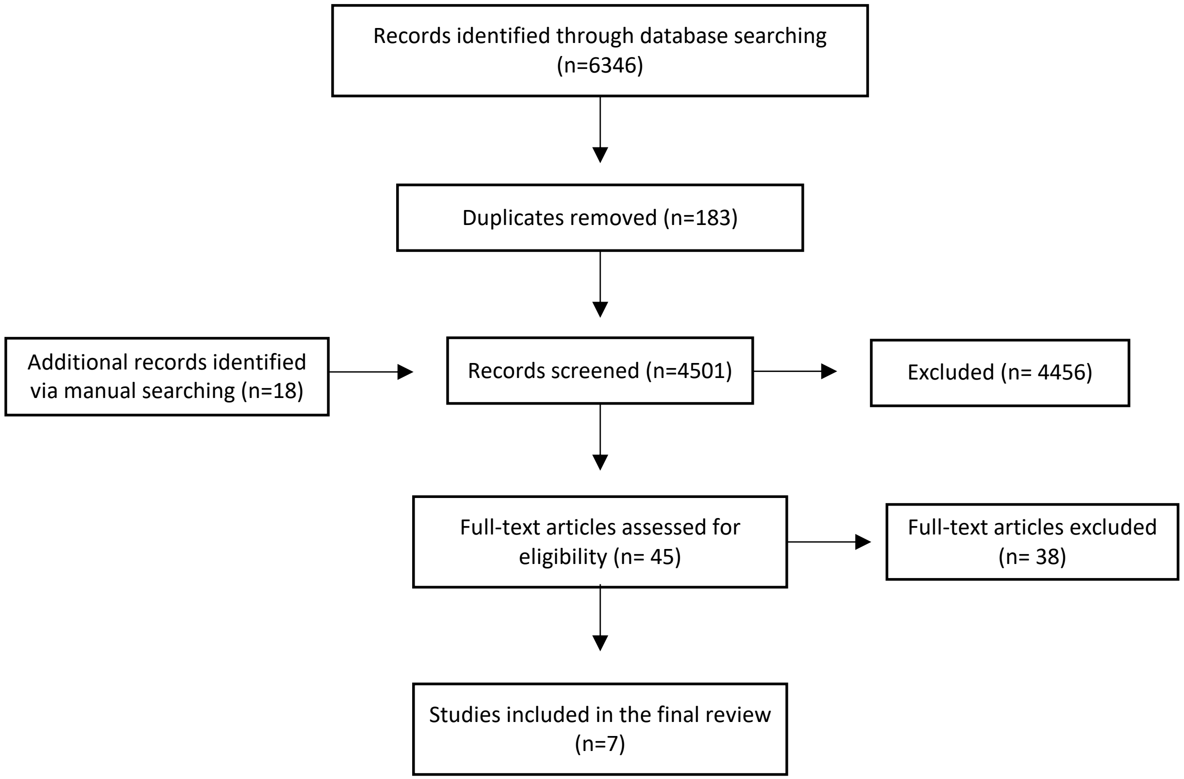
Records identified through database searching (n=6346)
Duplicates removed (n=183)
Additional records identified via manual searching (n=18)
Records screened (n=4501)
Excluded (n= 4456)
Full-text articles assessed for eligibility (n= 45)
Full-text articles excluded (n= 38)
Studies included in the final review (n=7)

Appendix 1. Search strategy in all databases

Database Searched	Ethnicity Terms	Outcome Terms (Mental Health)	Geography Terms	Population terms
**Medline**	minority groups.mp. OR Minority Groups/ OR ethnic groups.mp. OR exp Ethnic Groups/ OR ethnic*.mp. OR Race.mp. OR exp Continental Population Groups/ OR racial.mp. OR<br><br>Black.mp. OR exp African Continental Ancestry Group/ OR Black African*.mp OR British African*.mp OR (Black adj1 British).mp. OR Afro$Caribbean*.mp OR Black adj1 caribbean.mp OR (Afr* adj3 Caribbean) OR Black*.mp. OR<br><br>Asian.mp. OR exp Asian Continental Ancestry Group/ OR Indian*.mp. OR exp Pakistan/ OR Pakistan*.mp. OR Bangladesh*.mp. OR exp Bangladesh/ OR Bengali*.mp/ OR Chinese.mp. OR<br><br>Arab*.mp. OR exp Arabs OR	Mental Health.mp. OR exp Mental Health/ OR mental well$being.mp OR exp Stress, Psychological/ OR psychological well$being.mp. OR Resilience, Psychological/ OR resilien*.mp. OR exp Occupational Stress OR stress.mp.	exp United Kingdom/ OR UK.mp OR United Kingdom.mp. OR Great Britain.mp OR Britain.mp OR GB.mp OR England.mp OR Scotland.mp OR Wales.mp OR North* Ireland.mp OR British.mp OR Scottish.mp OR Welsh.mp OR North* Irish.mp	Adult*<br>Wom?n<br>Men<br>people

	Gypsy or “Irish traveller” OR<br><br>(white and black African*).mp. OR (white and black caribbean).mp. OR (white and Asian).mp. OR<br>mixed ethnicit*.mp			
**Embase**	minority groups.mp. OR exp Minority Groups/ OR ethnic groups.mp. OR exp Ethnic Group/ OR ethnic*.mp. OR exp race/ OR “ethnic or racial aspects”/ OR Race.mp. OR racial.mp. OR<br><br>Black*.mp. OR exp Black person/ OR black african*.mp OR British African*.mp OR (Black adj1 British).mp. OR Afro$Caribbean*.mp OR (Black adj1 caribbean).mp OR (Afr* adj3 Caribbean).mp. OR<br><br>Asian.mp. OR exp Asian/ OR exp Asian continental ancestry group/ OR Indian*.mp. OR exp Indian/ OR Pakistan*.mp. OR exp Pakistan/ OR exp Bangladesh/ OR Bangladesh*.mp. OR Bengali*.mp OR exp “Bengali (people)”/ OR Chinese.mp. OR exp Chinese/ OR	Mental Health.mp. OR exp Mental Health/ OR [mental well$being.mp] OR exp psychological well-being/ OR psychological stress.mp. OR exp mental Stress/OR psychGological well$being.mp. OR exp psychological resilience/ OR resilien*.mp OR job stress/ OR life stress/ OR chronic stress/ OR emotional stress/ OR social stress/ OR behavioural stress/ OR stress.mp.	Exp united Kingdom/ OR United Kingdom.mp. OR UK.mp. OR Great Britain.mp OR Britain.mp OR GB.mp OR England.mp OR Scotland.mp OR Wales.mp OR North* Ireland.mp OR British.mp OR Scottish.mp OR Welsh.mp OR North* Irish.mp	Adult*<br>Wom?n<br>Men<br>people

For peer review only - http://bmjopen.bmj.com/site/about/guidelines.xhtml

	Arab*.mp. OR exp Arab/ OR<br><br>Gypsy or "Irish traveller" OR<br><br>(white and black African*).mp. OR (white and black Caribbean*).mp. OR (white and Asian*).mp.] OR mixed ethnic*.mp			
**PsychINFO**	minority groups.mp. OR exp Minority Group/ OR ethnic groups.mp. OR exp 'Racial and Ethnic groups'/ OR ethnic*.mp. OR Race.mp. OR racial.mp. OR 'race and ethnic discrimination'/ OR<br><br>exp Blacks/ OR black*.mp. OR exp African Cultural Groups/ or black African*.mp. OR British African*.mp OR (Black adj1 British).mp. OR Afro$Caribbean*.mp OR (Black adj1 caribbean).mp OR (afr* adj3 Caribbean).mp. OR Asian.mp. OR exp Asians/ OR Indian*.mp. OR Pakistan*.mp. OR Bangladesh*.mp. OR Bengali*.mp OR Chinese.mp. OR<br><br>Arab*.mp. OR<br><br>Gypsy or "Irish traveller" OR	Mental Health.mp. OR exp Mental Health/ OR mental well$being.mp OR exp Well Being/ OR exp Psychological Stress/or psychological well$being.mp. OR exp "Resilience (Psychological)"/OR resilien*.mp. OR exp Occupational Stress/ OR stress.mp. OR exp Chronic stress/ OR exp Social Stress/	United Kingdom.mp. OR UK.mp. OR Great Britain.mp OR Britain.mp OR GB.mp OR England.mp OR Scotland.mp OR Wales.mp OR North* Ireland.mp OR British.mp OR Scottish.mp OR Welsh.mp OR North* Irish.mp	Adult*<br>Wom?n<br>Men<br>people

For peer review only - http://bmjopen.bmj.com/site/about/guidelines.xhtml

	(white and black African*).mp. OR (white and black Caribbean*).mp. OR (white and Asian*).mp. OR<br><br>mixed ethnicit*.mp			
**SCOPUS**	("minority group*" OR ethnic* OR race OR racial OR african* OR (black W/1 british) OR afro$caribbean* OR (black W/1 caribbean) OR indian OR pakistani OR arab* OR asian OR indian OR bangladesh* OR bengali OR chinese OR "mixed ethnic*" OR "mixed race")	("mental health" OR "mental well?being" OR "psychological well?being" OR "psychological stress" OR "psychological resilien*")	"united kingdom" OR "Great Britain" OR England OR Scotland OR Wales OR (North* Ireland) OR British OR Scottish OR Welsh OR (North* Irish)	Adult*<br>Wom?n<br>Men<br><br>people
**CINAHL**	(minority group*) OR (ethnic* group*) OR ethnic* OR race OR racial OR "black African" OR "African Cultural Groups" OR (british W/1 african) OR (black W/1 british) OR Afro$ Caribbean* OR (black W/1 caribbean) OR (afr* W/3 caribbean) OR asian OR indian OR pakistan* OR bangladesh* OR bengali OR chinese OR arab* OR	(Mental Health) OR (mental well?being) OR (Well?Being) OR (Psychological Stress) OR (psychological well?being) OR exp (psychological resilien*) OR (Chronic stress) OR (Social Stress)	"united kingdom" OR "Great Britain" OR England OR Scotland OR Wales OR (North* Ireland) OR British OR Scottish OR Welsh OR (North* Irish)	Adult*<br>Wom?n<br>Men<br><br>people

For peer review only - http://bmjopen.bmj.com/site/about/guidelines.xhtml

	“mixed ethnicit*” or Gypsy or “Irish traveller”			
**Cochrane**	"minority group*" OR ethnic* OR race OR racial OR african* OR (black near/1 british) OR afro$caribbean* OR (black near/1 caribbean) OR asian OR indian OR pakistan* OR bangladesh* OR bengali* OR chinese OR arab* OR "mixed ethnicit*" OR gypsy OR “Irish traveller”	(Mental Health) OR (mental well?being) OR (Well?Being) OR (Psychological Stress) OR (psychological well?being) OR exp (psychological resilien*) OR (Chronic stress) OR (Social Stress)	“united kingdom” OR “Great Britain” OR England OR Scotland OR Wales OR (North* Ireland) OR British OR Scottish OR Welsh OR (North* Irish)	Adult* Wom?n Men<br><br>people

For peer review only

Preferred Reporting Items for Systematic reviews and Meta-Analyses extension for Scoping Reviews (PRISMA-ScR) Checklist

SECTION	ITEM	PRISMA-ScR CHECKLIST ITEM	REPORTED ON PAGE #
**TITLE**			
Title	1	Identify the report as a scoping review.	1
**ABSTRACT**			
Structured summary	2	Provide a structured summary that includes (as applicable): background, objectives, eligibility criteria, sources of evidence, charting methods, results, and conclusions that relate to the review questions and objectives.	2
**INTRODUCTION**			
Rationale	3	Describe the rationale for the review in the context of what is already known. Explain why the review questions/objectives lend themselves to a scoping review approach.	5,6
Objectives	4	Provide an explicit statement of the questions and objectives being addressed with reference to their key elements (e.g., population or participants, concepts, and context) or other relevant key elements used to conceptualize the review questions and/or objectives.	5
**METHODS**			
Protocol and registration	5	Indicate whether a review protocol exists; state if and where it can be accessed (e.g., a Web address); and if available, provide registration information, including the registration number.	6
Eligibility criteria	6	Specify characteristics of the sources of evidence used as eligibility criteria (e.g., years considered, language, and publication status), and provide a rationale.	7,8
Information sources*	7	Describe all information sources in the search (e.g., databases with dates of coverage and contact with authors to identify additional sources), as well as the date the most recent search was executed.	6
Search	8	Present the full electronic search strategy for at least 1 database, including any limits used, such that it could be repeated.	appendix
Selection of sources of evidence†	9	State the process for selecting sources of evidence (i.e., screening and eligibility) included in the scoping review.	9
Data charting process‡	10	Describe the methods of charting data from the included sources of evidence (e.g., calibrated forms or forms that have been tested by the team before their use, and whether data charting was done independently or in duplicate) and any processes for obtaining and confirming data from investigators.	10
Data items	11	List and define all variables for which data were sought and any assumptions and simplifications made.	10
Critical appraisal of individual sources of evidence§	12	If done, provide a rationale for conducting a critical appraisal of included sources of evidence; describe the methods used and how this information was used in any data synthesis (if appropriate).	10,11

For peer review only - http://bmjopen.bmj.com/site/about/guidelines.xhtml

SECTION	ITEM	PRISMA-ScR CHECKLIST ITEM	REPORTED ON PAGE #
Synthesis of results	13	Describe the methods of handling and summarizing the data that were charted.	10,11
**RESULTS**			
Selection of sources of evidence	14	Give numbers of sources of evidence screened, assessed for eligibility, and included in the review, with reasons for exclusions at each stage, ideally using a flow diagram.	10
Characteristics of sources of evidence	15	For each source of evidence, present characteristics for which data were charted and provide the citations.	14
Critical appraisal within sources of evidence	16	If done, present data on critical appraisal of included sources of evidence (see item 12).	17-22
Results of individual sources of evidence	17	For each included source of evidence, present the relevant data that were charted that relate to the review questions and objectives.	17-22
Synthesis of results	18	Summarize and/or present the charting results as they relate to the review questions and objectives.	14,15
**DISCUSSION**			
Summary of evidence	19	Summarize the main results (including an overview of concepts, themes, and types of evidence available), link to the review questions and objectives, and consider the relevance to key groups.	23
Limitations	20	Discuss the limitations of the scoping review process.	24,25
Conclusions	21	Provide a general interpretation of the results with respect to the review questions and objectives, as well as potential implications and/or next steps.	26,27
**FUNDING**			
Funding	22	Describe sources of funding for the included sources of evidence, as well as sources of funding for the scoping review. Describe the role of the funders of the scoping review.	27

JBI = Joanna Briggs Institute; PRISMA-ScR = Preferred Reporting Items for Systematic reviews and Meta-Analyses extension for Scoping Reviews.
* Where *sources of evidence* (see second footnote) are compiled from, such as bibliographic databases, social media platforms, and Web sites.
† A more inclusive/heterogeneous term used to account for the different types of evidence or data sources (e.g., quantitative and/or qualitative research, expert opinion, and policy documents) that may be eligible in a scoping review as opposed to only studies. This is not to be confused with *information sources* (see first footnote).
‡ The frameworks by Arksey and O'Malley (6) and Levac and colleagues (7) and the JBI guidance (4, 5) refer to the process of data extraction in a scoping review as data charting.
§ The process of systematically examining research evidence to assess its validity, results, and relevance before using it to inform a decision. This term is used for items 12 and 19 instead of "risk of bias" (which is more applicable to systematic reviews of interventions) to include and acknowledge the various sources of evidence that may be used in a scoping review (e.g., quantitative and/or qualitative research, expert opinion, and policy document).

From: Tricco AC, Lillie E, Zarin W, O'Brien KK, Colquhoun H, Levac D, et al. PRISMA Extension for Scoping Reviews (PRISMAScR): Checklist and Explanation. Ann Intern Med. 2018;169:467–473. doi: 10.7326/M18-0850.

For peer review only - http://bmjopen.bmj.com/site/about/guidelines.xhtml

BMJ Open

Community-centred interventions for improving public mental health among adults from minority ethnic populations in the United Kingdom: a scoping review

Journal:	*BMJ Open*
Manuscript ID	bmjopen-2020-041102.R2
Article Type:	Original research
Date Submitted by the Author:	17-Feb-2021
Complete List of Authors:	Baskin, Cleo; Imperial College London School of Public Health, Department of Primary Care and Public Health<br>Zijlstra, Geiske; Imperial College London School of Public Health, Department of Primary Care and Public Health<br>McGrath, Mike; UCL, Division of Psychiatry<br>Lee, C; University of Cambridge Institute of Public Health, Forvie Site<br>Duncan, Fiona; Durham University, Sport and Exercise Sciences<br>Oliver, Emily; Durham University, Department of Sport and Exercise Sciences<br>Osborn, David; UCL, Division of Psychiatry; Camden and Islington NHS Foundation Trust<br>Dykxhoorn, Jen; UCL, Division of Psychiatry; UCL, Department of Primary Care and Population Health<br>Kaner, Eileen; Newcastle University, Population Health Sciences Institute<br>LaFortune, Louise; University of Cambridge, Institute of Public Health<br>Walters, Kate; University College London, Department of Primary Care and Population Health<br>Kirkbride, James; University College London, Division of Psychiatry<br>Gnani, Shamini; Imperial College London School of Public Health, Department of Primary Care and Public Health
**Primary Subject Heading**:	Mental health
Secondary Subject Heading:	Health policy, Mental health, Public health
Keywords:	MENTAL HEALTH, PUBLIC HEALTH, Adult psychiatry < PSYCHIATRY

SCHOLARONE™
Manuscripts

I, the Submitting Author has the right to grant and does grant on behalf of all authors of the Work (as defined in the below author licence), an exclusive licence and/or a non-exclusive licence for contributions from authors who are: i) UK Crown employees; ii) where BMJ has agreed a CC-BY licence shall apply, and/or iii) in accordance with the terms applicable for US Federal Government officers or employees acting as part of their official duties; on a worldwide, perpetual, irrevocable, royalty-free basis to BMJ Publishing Group Ltd ("BMJ") its licensees and where the relevant Journal is co-owned by BMJ to the co-owners of the Journal, to publish the Work in this journal and any other BMJ products and to exploit all rights, as set out in our licence.

The Submitting Author accepts and understands that any supply made under these terms is made by BMJ to the Submitting Author unless you are acting as an employee on behalf of your employer or a postgraduate student of an affiliated institution which is paying any applicable article publishing charge ("APC") for Open Access articles. Where the Submitting Author wishes to make the Work available on an Open Access basis (and intends to pay the relevant APC), the terms of reuse of such Open Access shall be governed by a Creative Commons licence – details of these licences and which Creative Commons licence will apply to this Work are set out in our licence referred to above.

Other than as permitted in any relevant BMJ Author's Self Archiving Policies, I confirm this Work has not been accepted for publication elsewhere, is not being considered for publication elsewhere and does not duplicate material already published. I confirm all authors consent to publication of this Work and authorise the granting of this licence.

For peer review only

Community-centred interventions for improving public mental health among adults from minority ethnic populations in the United Kingdom: a scoping review

Baskin C[1*], Zijlstra G[1*], McGrath M [3], Lee C[6], Duncan F[2], Oliver E. J.[2], Osborn D[3,7], Dykxhoorn J[3,4], Kaner E[5], Lafortune L[6], Walters K[4], Kirkbride JB[3], Gnani S[1]

*Cleo Baskin and Geiske Zijlstra contributed equally to this paper.

1 Department of Primary Care and Public Health, School of Public Health, Imperial College London, Reynolds Building, St Dunstan's Road, London, W6 8RP.

2 Department of Sport and Exercise Sciences, Durham University, 42 Old Elvet, Durham, DH1 3HN,

3 Division of Psychiatry, UCL, Maple House, 149 Tottenham Court Road, London, W1T 7NF.

4 Department of Primary Care and Population Health, UCL Royal Free Campus, Rowland Hill Street, London NW3 2PF

5 Population Health Sciences Institute, Baddiley-Clark Building, Newcastle upon Tyne, NE2 4AX.

6 Cambridge Institute of Public Health, Forvie Site, Robinson Way, Cambridge, CB2 0SR.

7 Camden and Islington NHS Foundation Trust, NW1OPE

Corresponding author: Cleo Baskin

Department of Primary Care and Public Health, Imperial College London

Reynolds Building, St Dunstan's Road, London, W6 8RP

Phone: +447366416304

Email: cleo.baskin18@ic.ac.uk

Word count: 3998

For peer review only

Abstract

Objectives: Undertake a scoping review to determine the effectiveness of community-centred interventions designed to improve the mental health and well-being of adults from Black, Asian and Minority Ethnic (BAME) populations in the United Kingdom.

Methods: We searched six electronic academic databases for studies published between January 1990-September 2019: Medline, Embase, PsychINFO, Scopus, CINAHL and Cochrane. We used the Preferred Reporting Items for Systematic Reviews and Meta-Analyses (PRISMA) extension for Scoping Reviews checklist and Template for Intervention description and replication (TIDieR) guide for intervention description and data extraction. Quality was assessed using Cochrane Risk of Bias tools. Grey literature results were deemed beyond the scope of this review; preliminary searches found numerous (>50) small scale community interventions without available outcomes data.

Results: Of 4,501 studies, seven met the eligibility criteria of UK-based community interventions targeting mental health in BAME adults: four randomised control trials (RCT), one pre- and post-pilot study, one cross-sectional study and one ethnographic study. Interventions included therapy-style sessions, peer-support groups, educational materials, gym access, and a family services programme. Common components included a focus on tackling social isolation, using lay health workers from within the community, signposting, and cultural adaptation. Only three studies reported a statistically significant positive outcome on mental health outcomes and six were appraised as having a high risk of bias. Study populations were ethnically heterogenous and targeted people mainly from South Asia. Knowledge gaps emerged around effective interventions for men, populations

For peer review only

such as Chinese, Arab and Travellers, and tackling the wider determinants of mental health.

Conclusions: There is a paucity of high-quality evidence regarding community-based interventions focussed on improving public mental health among BAME groups. Decision makers need scientific evidence to inform effective approaches to mitigating health disparities. Our next steps are to map promising community activities and interventions that are currently being provided to help identify emerging evidence.

Keywords

Mental health, well-being, Black, Asian and Minority Ethnic groups, minority groups, ethnicity, community-based

Strengths and limitations of this study

- This scoping review addresses the large evidence gap which exists on the effectiveness of community-centred interventions in improving public mental health of UK Black, Asian and Minority Ethnic (BAME) populations, who are disproportionately affected by poor mental health.
- A six-stage scoping review framework, including stakeholder consultation, was applied to summarise the evidence for community-centred interventions.
- We included all community interventions irrespective of setting and type.
- Analysis of review data were limited to studies published in peer-reviewed journals and citation tracking of references.
- We used national definitions for BAME categories but acknowledge challenges in viewing ethnic minority groups as homogenous in their mental health needs.

For peer review only - http://bmjopen.bmj.com/site/about/guidelines.xhtml

Introduction

Poor mental well-being disproportionately affects people from minority ethnic populations in the UK(1-8). Bias, racism and discrimination have had implications on many societal, structural and institutional risk factors for poor mental health, including worse employment(9), housing conditions(10), and healthcare(11) and increased likelihood to enter the criminal justice system(12) and live in poverty(13). Stigma toward mental illness is also reported to be higher among minority ethnic groups(14).

These risks affect mental health through numerous pathways, for example stigma and the lack of racially, culturally and/or ethnically appropriate healthcare may cause delays in help-seeking, ultimately leading to poorer outcomes(15,16). Additionally, stress- both acute and chronic- can affect physical health and mental wellbeing. The COVID-19 pandemic has exacerbated these risks, and has had a disproportionate impact on ethnic minorities in the UK(17).

In the UK, the term Black, Asian, and Minority Ethnic(BAME) is frequently used to refer to individuals who are in a racial or ethnic minority(18). However, this umbrella term groups together a population with differential and complex risks for poor mental health(19,20). For example, the risk of psychosis among the Black Caribbean population is nearly seven times higher than the White British population(21), but not for Asian groups(22).

Strengthening public mental health involves both the promotion of mental health and wellbeing and the prevention of mental illness. It has the potential to effectively and sustainably reduce social and racial inequalities in mental health outcomes(23,24). In

For peer review only

For peer review only - http://bmjopen.bmj.com/site/about/guidelines.xhtml

pursuit of this, many UK initiatives have been implemented, such as the 2005 'Delivering Race Equality in Mental Healthcare Action Plan'(25), but have failed to successfully narrow the observed mental health gap(26,27).

Community-centred interventions (i.e., those that take place in a community setting, or are delivered by the community and/or voluntary sector) have potential to influence the cultural and social factors that protect and promote mental health and wellbeing. These include social connectedness, access to safe and affordable housing, and power in local decision making(28,29). In 2017 the National Health Service (NHS) England introduced local financial incentives to improve mental health outcomes for BAME groups(30). However, local decision making has been hindered by a lack of evidence. The aim of this scoping review was to summarise the evidence for community-centred interventions focussed on improving the public mental health of BAME groups in the UK.

For peer review only

For peer review only - http://bmjopen.bmj.com/site/about/guidelines.xhtml

Methods

We undertook a scoping review, an approach for summarizing evidence and identifying knowledge gaps in unclear and emerging fields(31,32), using Arksey and O'Malley's six-stage methodological framework(33). The sixth stage, stakeholder consultation, was undertaken by involving stakeholders and peer researchers as part of a wider research programme(34). Scoping reviews were not eligible for prospective registration with the international prospective register for systematic reviews (PROSPERO) at the time of conducting the review(35).

I) Study identification

A systematic search of six electronic databases between January 1990 and September 2019 was conducted: Medline, Embase, PsycINFO, Scopus, CINAHL and Cochrane. The search strategy (Appendix 1) was created with the support of a medical librarian and was based on the PICOC (population; intervention; comparison; outcomes; context) framework. It included key terms for ethnicity, age range, geography and mental health outcomes, but no intervention and comparison terms were included to optimise capture of all relevant studies.

We excluded evidence from the grey literature as an initial search of primary data from local government, relevant third sector and NHS websites identified numerous activities and possible interventions (over 50 in a small geographical area). Information was provided in formats such as a flyer or a website or Facebook page describing services and activities, but with limited descriptions of the community intervention and outcomes data. Consequently, data synthesis exceeded the methodological approach of a scoping review; a mapping methodology would be more appropriate(36).

For peer review only - http://bmjopen.bmj.com/site/about/guidelines.xhtml

Eligibility criteria

Table 1 outlines the inclusion and exclusion criteria used to determine the eligibility of a study. We included studies published 1990 onwards so that our findings would inform contemporary policy and practice. Furthermore, systematic ethnicity data collection in the UK began with the 1991 Census(37). We included only UK studies due to variation in how race, ethnicity and ancestry are represented in different countries. This is a consequence of each countries' unique pattern of migration and its political and social context.

We included studies where participants were working aged adults (aged 18-64 years adapted from the UK definition of 16-64 years) who were either well or had a common mental disorder, such as anxiety and depression. Common mental disorders were included to reflect the high prevalence of these conditions in the general population, one in six adults in England(38). They often go undiagnosed and so are more amenable to interventions focused on prevention and the promotion of positive mental health(39). Studies focusing on people with severe mental illness (including suicide and psychotic disorders), or people affected by young onset dementia, were not included due to the need for specialist mental health treatment and tailored support. Studies that specifically targeted new migrants and refugee groups were also excluded due to the specific and complex mental health needs of this population, such as the high prevalence of post-traumatic stress disorder (40,41). Studies were included if new migrants or refugees happened to be recruited as participants but not if the study's intervention was specifically designed for them.

For peer review only - http://bmjopen.bmj.com/site/about/guidelines.xhtml

Only community-centred interventions were eligible for inclusion. We defined community-centred broadly to include all interventions that were not clinical in nature, that is, did not involve the treatment of patients by health professionals such as cognitive behavioural therapy and drug therapy(42,43). Therapy-style sessions that were delivered by lay health workers were included. There is a wide range of overlapping concepts in community- based and community- centred terms, and these were captured by our broad search strategy.

Table 1. Eligibility criteria

Inclusion criteria	Exclusion criteria
Adults aged 18 – 64 years	Children aged 0 -17 or adults aged over 64 years
Individuals from Asian/ Asian British, Black/ African/ Caribbean/ Black British, Mixed/Multiple ethnic groups and Arab ethnic groups	All White British ethnicities other than Gypsy or Irish Traveller, refugees and asylum seekers
Adults with no known mental disorder or diagnosed with a common mental disorder, such as anxiety and depression	Adults with severe mental illness and dementia
Community-centred interventions, i.e., take place in a community setting, or in a health setting but delivered by the community and/or voluntary sector	Clinical interventions that involved treatment by a clinician in any setting
Study measures any domain or aspect of mental health and well-being	No outcome measures for mental health or well-being
All studies based on primary research	Abstracts, posters, books or chapters, editorials and letters
Study conducted in the UK	Study conducted outside of the UK
Published from 1990 onwards	Published before 1990
Full-text research article available	No full text research article available

The UK Office of National Statistics 2011 Census ethnicity terms were used (Table 2)(44). Ethnic groups were deemed a minority if they fell under 'Mixed', 'Asian', 'Black'

For peer review only - http://bmjopen.bmj.com/site/about/guidelines.xhtml

and 'Other' categories. All ethnic groups under the 'White' category were excluded, except for the 'Gypsy or Irish traveller' group due to their comparatively higher social and economic disadvantage and observed health inequalities(8).

Table 2. Ethnicity as Classified by the Office for National Statistics

**White**	**English / Welsh / Scottish / Northern Irish / British**
	Irish
	Gypsy or Irish Traveller
	Any other White background
**Mixed / Multiple ethnic groups**	White and Black Caribbean
	White and Black African
	White and Asian
	Any other Mixed / Multiple ethnic background
**Asian / Asian British**	Indian
	Pakistani
	Bangladeshi
	Chinese
	Any other Asian background
**Black / African / Caribbean / Black British**	African
	Caribbean
	Any other Black / African / Caribbean background
**Other ethnic group**	Arab
	Any other ethnic group

II) Study selection

Two independent reviewers(GZ,CB) screened non-duplicate titles and abstracts against eligibility criteria (n=4501). The abstracts that matched criteria (Table 1) were reviewed in full be GZ and CB (n=45). All conflicts were resolved by a third reviewer (SG); this was needed for 31% (n=14) of full text articles. Full texts that were not freely available were accessed via the British Library. Additional articles were identified by examining references and forward-backward citation searching (Figure 1). Covidence systematic review software(45) was used to remove duplicates and screen titles.

III) Charting of data

Data were extracted by two independent reviewers(GZ,CB). A data extraction framework was developed to capture key study characteristics: author; year of

For peer review only - http://bmjopen.bmj.com/site/about/guidelines.xhtml
For peer review only

publication; setting; study design; population demographics; sample size; theory of change; and outcomes and results. Intervention details were extracted using the Template for Intervention Description and Replication(TIDieR) guide: rationale; materials; procedures; provider; methods; location; timing; tailoring; modifications; and assessment of intervention adherence(46).

IV) Collation and summary of results

Quality appraisal was performed by two reviewers(GZ,CB) using the Cochrane tool for risk of bias for RCTs(47). Qualitative studies were appraised using the Cochrane Qualitative and Implementation Methods Group guidance and the Mixed-Method Appraisal Tool(MMAT) for mixed methods studies(48,49). A narrative data synthesis of the papers was conducted by identifying themes and mechanisms common to the community-based interventions. Review findings were reported using The Preferred Reporting Items for Systematic Reviews and Meta-Analyses (PRISMA) extension for Scoping Reviews checklist(50).

V) Patient and Public Involvement

The research question was informed by peoples' experiences and stakeholder workshops. This study did not involve the recruitment of patients, and no patients were involved in the design, or conduct of the study.

For peer review only - http://bmjopen.bmj.com/site/about/guidelines.xhtml

Results

We identified a total of 4,483 non-duplicate titles and an additional 18 from forward-backward citation searching (Figure 1). Seven out of the 45 studies reviewed in full-text matched the eligibility criteria and were included in this review: four RCTs (including one pilot); one pre- and post-intervention pilot study; one cross sectional; and one ethnographic study (Table 3).

Intervention type and focus

The interventions were highly heterogenous in design and focus. They included therapy-style sessions delivered by lay health workers(51,52), peer-support groups(53,54), an educational leaflet(55), free gym access(56), and a family services programme(57). All interventions were conducted in England. Certain subgroups were targeted more than others with interventions specifically tailored to individuals of Indian, Pakistani and Bengali heritage(n=3), women(n=4), and people with anxiety or depression(n=5).

Interventions were either a single session(n=1) or took place over a minimum of 10-weeks to eight months(n=6). The duration of the family services programme is unclear. Four studies aimed to improve mental health by expanding social networks and facilitating social support(51,53,54,57) and four aimed to increase access to services by facilitating linkages or signposting to complementary social care and mental health services (51,52,53,57).

Qualitative data commonly found a reduction in social isolation and stress, and improved mood and self-confidence(52,54,56,57). One RCT reported significant

For peer review only - http://bmjopen.bmj.com/site/about/guidelines.xhtml

improvement in social functioning(53) and two had a statistically significant positive impact on depression outcomes(54,55). All but one study(53) were appraised as having a high risk of bias with limitations relating to small sample sizes (n=9-123, mean=43, median=30), short follow-up periods (ranging from two to nine months), and participant recruitment.

For peer review only

For peer review only - http://bmjopen.bmj.com/site/about/guidelines.xhtml

Table 3. Summary of community-based intervention studies targeted at Black, Asian and Minority Ethnic groups to improve mental health and well-being (N=7)

Author; year	Study design	Participant characteristics	Theory of Change	Intervention description	Results
**Jacob et al; 2002 (55)**	RCT	Asian women with depression in Ealing, London	Educate patients on depression to positively affect their perspective and outcomes of depression	Participants (n=70) received an educational leaflet about depression or usual care	**GHQ-12**: recovery from common mental disorder (defined as $\leq$ 2): OR 3.4 (95% CI: 1.01 to 11.5) at 2 months
**Gater et al; 2010 (53)**	RCT	British Pakistani women with depression in Northwest England	Improve mental health through reducing social isolation	Participants (n=123) received a social group intervention or antidepressant medication, or both	**HRSD** (depression): no significant effects<br><br>**Social functioning**: Significant effect at 3 months for social intervention vs. antidepressants, 6.1 (95% CI: 1.4 to 10.8), p= 0.02, and combined group vs. antidepressants, 5.9 (95% CI: 1.5 to 10.2), p= 0.017. No significant effect at 9 months<br><br>**Satisfaction**: Significant effects at 3 and 9 months for social vs. antidepressants, 4.9 (95% CI: 2.9 to 7.0), p=0.002; 4.7 (95% CI: 2.6 to 6.7), 0.002 and combined v. antidepressants, 4.3 (95% CI: 2.6 to 6.0), p=0.001; 4.0 (95% CI: 1.8 to 6.2), p=0.005<br><br>Qualitative feedback found a major hindrance to participation was resistance from family members. Participants engaged in the trial because of the culturally appropriate format and content of the sessions. They described their experience as ‘relief from worries’, ‘feeling fresh’ and ‘better than their expectations’.
**Afuwape et al; 2010 (51)**	RCT	Black adults with anxiety and/or depression in Southwark, London	Improve psychosocial functioning and feelings of hope through a culturally acceptable care package	Participants (n=40) received individual therapy and group sessions on advice on services, health education and mentoring	**GHQ-29**: Significant effect at 3 months: adjusted mean difference 7.76 (95% CI: 0.86 to 14.65), p = 0.03<br><br>**GAF**: no significant effect<br><br>**SF-36**: mental health components had significant effect, −11.93 (95% CI: −21.99 to −1.88), p=0.02.<br>No effect on physical health. No significant differences in cost

For peer review only - http://bmjopen.bmj.com/site/about/guidelines.xhtml

**Lovell et al; 2014 (52)**	Pilot RCT	South Asian and Somalian women with moderate depression and/or anxiety in Northwest England	Improve treatment of depression and anxiety using social and community interventions	Participants (n=20) received individual or group therapy-style sessions, or usual care. Activities focussed on health and wellbeing; sign posting to service and education opportunities	**CORE-OM:** no effect<br><br>Small non-significant improvements in depression (PHQ9), health related quality of life (EQ5D) and functioning (WSAS) at 5 months<br><br>Qualitative data suggested that patients found the intervention acceptable, both in terms of content and delivery.
**Chaudhry et al; 2009 (54)**	Observational: Pre- and post-intervention	British Pakistani women diagnosed with depression In Manchester	Informal social support and mental and physical health education to reduce depression	Participants (n=9) received 10 weekly group sessions in various locations: psycho education, personal grooming, exercise and yoga	**SRQ**: reduction in depression scores pre- 15 (SD = 3.08) to post - 11.7 (SD = 5.95), p = 0.039.<br>SCAN interviews post-intervention diagnosed 2 participants as no longer depressed<br><br>**SCAN:** interviews post-intervention diagnosed 2 participants as no longer depressed<br><br>Anecdotal feedback from the participants identified that the relationships developed between the participants and facilitators and the provision of transport were the most important components of the intervention.
**Rabiee et al; 2015 (56)**	Cross-sectional; mixed methods	BAME groups in a deprived constituency in Birmingham	Regular exercise can help improve mood, self-esteem, confidence and quality of life	Gym for Free pilot project providing adults free access to leisure centres	Results indicate increased energy levels, confidence, mental well-being, reduction in stress and anxiety improved stress relief, and anger management
**Gray; 2003 (57)**	Ethnographic investigation	BAME families in Tower Hamlets, London	Reducing social isolation and poverty by improving parenting skills, self-esteem and meeting a welfare advisor	Personalised family support workers; trained volunteers of similar ethnic and cultural backgrounds	Family case records and interviews suggest close relationships were formed with the support workers of the same ethnic and cultural identity. They also portray a reduction in social isolation, bullying and racism Increased advocacy with other professionals and access to relevant services

For peer review only - http://bmjopen.bmj.com/site/about/guidelines.xhtml

Abbreviations: GHQ-12, General Health Questionnaire-12; HRSD, Hamilton Rating Scale for Depression; GHQ-29, General Health Questionnaire 29; GAF, Global assessment of Functioning; SF-36, Short form survey 36; CORE-OM, Clinical Outcomes in Routine evaluation- outcome measure scale; SRQ, Self-Reporting Questionnaire; SCAN, Schedule for clinical assessment in Neuropsychiatry

For peer review only

Outcome measures

Mental health outcomes were predominantly measured using standardised screening instruments for common mental disorders and social functioning (Table 4). Qualitative data were predominantly collected through focus groups and semi-structured interviews(52-54,56,57). Only one study reported on costs, calculated by measuring the difference in use of health and social services between the intervention and control group(51).

Table 4. Mental health and wellbeing outcome measures reported in the included studies (n = 7)

Author; year	Quantitative measures	Qualitative outcomes
Jacob et al; 2002	• **GHQ-12:** General Health Questionnaire 12<br>• **Short Explanatory Model Interview (SEMI):** self-reported perspective on depression	None
Gater et al; 2010	• **HRSD:** Hamilton Rating Scale for Depression<br>• **Verona Service Satisfaction Scale** (adapted)<br>• **Social functioning** (measured using a tool specifically created for British Pakistani women)	Feedback forms and focus groups
Afuwape et al; 2010	• **GHQ-28:** General Health Questionnaire 28<br>• **GAF:** Global assessment of Functioning<br>• **SF-36:** Short form survey (measuring quality of life)<br>• **LEDS:** Life Events and Difficulties Schedule	None
Lovell et al; 2014	• **PHQ-9:** Patient Health Questionnaire (measuring depression<br>• **COREOM:** Clinical outcomes in routine evaluation – outcome measure scale (measuring global distress)<br>• **GAD-7:** Generalised Anxiety Disorder Assessment<br>• **WSAS:** Work and Social Adjustment Scale<br>• **EQ-5D** (measuring health related quality of life)	Semi-structured interviews regarding the acceptability of the intervention
Chaudhry et al; 2009	• **SRQ:** Self Reporting Questionnaire (Urdu version; screening for mental disorders)<br>• **SCAN:** The Schedule for Clinical Assessment in Neuropsychiatry	Anecdotal feedback collected by facilitators
Rabiee et al; 2015	• Self-completed questionnaire evaluating experience in accessing services and perceived changes in health and well-being	Focus groups analysed using established frameworks and guidelines (58,59)
Gray; 2003	None	Family case records and interviews analysed using established frameworks (60,61)

For peer review only - http://bmjopen.bmj.com/site/about/guidelines.xhtml

Following narrative synthesis, we identified three themes with the perspective of informing the delivery of public mental health interventions for BAME communities: addressing social isolation and loneliness, promoting access and use of services, and being delivered by lay health workers.

Addressing social isolation and loneliness

Chaudhry et al.(54) evaluated the efficacy and cultural acceptability of establishing a social group for British Pakistani women with depression. Women were diagnosed with depression using the Schedule for Clinical Assessment in Neuropsychiatry (SCAN) and were recruited from an ongoing population-based study. Participants attended at least six of ten weekly meetings at a community centre for the Pakistani community. Meetings included one session of psychoeducation and self-selected activities including personal grooming, exercise, yoga, and visits to museums and local shopping malls.

The intervention was successful in improving mental health outcomes; a significant reduction in depressive symptoms was found at the end of the 10 sessions (SRQ scores pre-intervention:15, SD=3.08; post-intervention:11.7, SD= 5.95, p=0.039) and three women reported a reduction in suicidal ideas. These outcomes, and participants' anecdotal feedback, suggest that a reduction in depressive symptoms is partly attributed to reducing social isolation by bringing the women together. Participants highly valued the provision of free transport by female Urdu speakers to avoid any objections from family and community members. Heterogeneity in the content and attendance of each session makes it difficult to assess the mechanism for improved

For peer review only - http://bmjopen.bmj.com/site/about/guidelines.xhtml

outcomes. The small sample size and lack of control group further limit the generalisability of these findings.

Chaudhry's pilot study formed the basis of the RCT conducted by Gater et al.(53). British Pakistani women (n=123) with depression were recruited from general practice surgeries. The women were randomised into one of three intervention arms: a social group intervention, antidepressant medication, or both the social group intervention and antidepressant medication. The social group involved ten women, who attended ten weekly sessions of social activities at a community centre. Facilitators were Urdu-speaking women who had completed a five-day training programme.

The intervention had no significant change in depression in all three arms. Social functioning significantly improved in both the social intervention and combined treatment groups at three months (6.1, 95% CI: 1.4,10.8 and 5.9, 95% CI:1.5,10.2) but not at 9 months. Focus groups, held at the end of each session, found that the intervention was too short and that a major barrier to participating was resistance from family members. Participants found it helpful to confide in others and were intending to maintain new friendships. Overall, this study was appraised as low risk and provides evidence in favour of social group interventions for short-term improvements of positive mental health.

Promoting access and use of services

Gray(57) evaluates a Family Support Service which provides home-based support and parenting lessons for families; support for parents with a severe mental illness; and help from support workers and the local authority in child protection. The service was

For peer review only - http://bmjopen.bmj.com/site/about/guidelines.xhtml

delivered by support workers who were recruited from the local community or had similar backgrounds to the client group.

Thirty case records of families were examined, and 22 interviews were conducted with support workers, families themselves, and professionals managing or referring to these services. Support workers were found to have had a positive impact on families by bringing them together; reducing social isolation, bullying and racism; promoting equality of access and pertinent use of services; and liaising successfully with other health and social care services. It was highly valued that projects were culturally sensitive and that support workers were of the same ethnicity. Power dynamics were important as facilitators' decisions were of potentially significant consequence to the families. This makes the support workers not only essential to delivering the intervention but also integral to achieving a positive outcome for families.

Jacob et al.(55) evaluated the effect of a culturally appropriate educational leaflet on depression delivered in general practice to women of Indian, Bangladeshi and Pakistani heritage (n=70). All participants had a common mental disorder, diagnosed through the General health Questionnaire (GHQ). Participants had their perspective (explanatory models) on depression assessed using the Short Explanatory Model Interview (SEMI). They then randomly received either a leaflet about the nature, causes, prevalence and treatment of depression or to usual care from their general practitioner. The leaflet was available in English, Hindi and Punjabi and read out to illiterate participants. After two months all participants repeated the GHQ and re-discussed the SEMI, particularly focusing on the idea of depression as an illness and whether medical help is necessary.

For peer review only - http://bmjopen.bmj.com/site/about/guidelines.xhtml

There was no statistically significant difference between the intervention and control group on the explanatory model of depression at follow-up. The number of women who recovered from depression (defined as GHQ-12 ≤ 2) at two months was significantly associated with receiving the educational leaflet (42.9% vs. 20% for controls, $p<0.05$; no median score of confidence intervals were reported). However, women in the intervention group had significantly lower baseline levels of psychiatric morbidity at entry into the trial, potentially explaining the observed differences between the two groups.

Rabiee et al.(56) conducted a survey questionnaire(n=257) and focus groups to evaluate the physical and mental health impact of a 'Gym for Free' scheme in four leisure centres. The survey was intended to elicit participants' experiences of accessing the service as well as information about their height and weight and perceived changes in their general health and wellbeing. Benefits of the scheme included improved confidence(n=92) and energy(n=121), reduced stress(n=77), and improved anger management(n=8).

Only nine participants participated in three focus groups: two men (one White and one Black) and seven women (four Pakistani, two Indian and one White). Participants valued building their social networks through the scheme and acknowledged the links between their physical and mental well-being. Although the authors suggest a financial barrier inhibiting people joining the gym, no formal data were collected on cost or participant finances. The generalisability of these findings is limited as participants

For peer review only - http://bmjopen.bmj.com/site/about/guidelines.xhtml

were selected through opportunistic sampling and may over-represent younger adults who are motivated to use the gym.

Delivered by lay health workers

Afuwape et al.(51) evaluated the London Cares of Life Project(CoLP) which is a complex social intervention designed to improve the mental health of adults of black African or black Caribbean origin(n=40). Participants with anxiety and/or depression, diagnosed using the World Health Organisation checklist criteria, were randomised to receive the intervention immediately or to a three-month waiting list control and information on local mental health services. During the three-month intervention, community health workers (psychology graduates trained to deliver the intervention) provided: practical advice to address social needs; health education; and gave brief therapies based on cognitive behavioural therapy principles.

Those who received the intervention showed significant improvement at 3-months in depressive symptoms (adjusted mean difference=7.76, CI 95%: 0.86 to 14.65) but not in general functioning (adjusted mean difference= -0.78, CI 95%: -10.40 to 8.84). The effects beyond three months are unknown. There was no difference in costings between the intervention and control group; health and social care utilisation costs were calculated for three months. The risk of bias is increased by the small sample size and participants in the non-intervention arm having a significantly greater prevalence of past psychiatric history. The generalisability to the wider BAME population is potentially limited due to the exclusion of non-English speaking participants and the use of screening tools that do not have clear evidence of validity in these populations.

For peer review only - http://bmjopen.bmj.com/site/about/guidelines.xhtml

Lovell et al.(52) examined the effect of a wellbeing intervention, based on cognitive behavioural strategies, aimed at decreasing anxiety, depression and social isolation in both BAME and elderly populations. Only results from the BAME group were included in this analysis (aged 21-58 years, n=20). Participants chose between receiving the intervention individually, as part of a group, or as signposting sessions. Interventions in all three arms were delivered by wellbeing facilitators, after a three-day training programme, who liaised with the participants' health and social care professionals. Individual interventions comprised eight 30-minute sessions over 16 weeks, groups were delivered 8-10 weekly two-hour sessions, and those who participated in signposting sessions were seen three times over a 16-week period.

This intervention had no statistically significant effect on mental health outcomes. The semi-structured interviews established that acceptability was enhanced by involving family and the community, having a health professional's recommendation, and having an empathetic facilitator. This study is limited by its small sample size and by having patients choose their intervention arm, albeit reflecting the realities of engagement and participation when delivering interventions.

For peer review only

Discussion

Main findings

We found seven studies of community-centred interventions that aimed to improve mental health and wellbeing among BAME groups in the UK. Although studies varied in design, four key interventional characteristics emerged.

First, interventions aimed to address social isolation through building peer-to-peer support and social networks(51,53,54,56). Evidence suggests BAME populations find it easier to start conversations on mental health within their own cultural networks rather than with health professionals; formal mental health services perceived as a last resort(62,63). We found that social support interventions mainly targeted women and it is unclear whether men would find these acceptable.

Second, interventions aimed to overcome cultural and other barriers in accessing care. For example, by translating educational materials(55), addressing practical considerations, such as travel(53,54) and using facilitators that shared the same ethnicity and/or life experience as participants(53-55,57). This concurs with emerging evidence that culturally adapted interventions lead to better outcomes among minority ethnic groups(64,65).

Third, interventions were delivered by lay health workers(51,52,57). Over recent years, task-shifting in healthcare from health professionals to lay health workers has been common due to the need to meet escalating demand for care(66). Lay health workers from the same community as patients may be perceived as more accessible(67) and help reduce the associated stigma of accessing mental health

For peer review only - http://bmjopen.bmj.com/site/about/guidelines.xhtml

services(68,69). They may also overcome language and cultural barriers that minority ethnic groups face when trying to communicate mental health needs to their healthcare provider(70,71). However, lay health workers, unless they have received anti-stigma training, may hold beliefs that are more stigmatising than those held by health professionals(72).

Four, interventions had an emphasis on signposting and or facilitating linkages to complementary, or additional services(51,52,53,57). Signposting has been widely adopted in England through social prescribing schemes, but to date there has been little emphasis on cultural appropriateness(73). Evidence suggests that mental health services provided by the voluntary and community sector and embedded in communities increases trust among BAME communities, which in turn promotes awareness of mental health problems and access to mental health services(2).

Of the community interventions we identified, we found more targeted South Asian ethnic groups, which may reflect these groups making up a greater proportion of the UK population. There were no interventions designed for and targeting men, despite men being less likely to seek help for common mental health problems(74).

Limitations

A limitation of this study was the omission of grey literature. Preliminary grey literature searching found over 50 community-centred services, which confirms that most relevant interventions reside outside publication in peer review journals and indicates significant publication bias. We were unable to include these interventions in this review due to a large number of individual activities/ interventions and the lack of

For peer review only

For peer review only - http://bmjopen.bmj.com/site/about/guidelines.xhtml

available primary data. We consider a mapping methodology to be more appropriate to comprehensively summarise this evidence(36). We also only included community-centred interventions that targeted BAME groups. It is possible that BAME groups access public mental health interventions through universal provision, yet uptake of screening programmes and utilisation of mental health care services shows BAME populations have lower use(75).

A further limitation is the generalisability of our results that include all BAME populations in the UK; homogenous recommendations cannot be made to a culturally and ethnically heterogenous population. Furthermore, we excluded all white ethnic minority groups apart from Irish Travellers. However, the UK has a white ethnic minority European population, such as people from Poland, who may be subject to similar systemic health inequalities and challenges to their mental health and well-being as other BAME groups(2).

Interventions specifically targeting refugees, asylum seekers and new migrants were also excluded. In the UK, refugee's makeup approximately 0.2%(76) of the population and new migrants 1.1%(77) and research indicates that these groups are more likely to require specialised clinical interventions focussed on reducing psychological trauma(78,79). However, due to the overall paucity of evidence in this field and the likelihood of shared need, future research should consider whether this exclusion is necessary.

For peer review only

Implications for policy makers and future research

We recognise that the evidence for ethnic differences in some common mental disorders is complex, variable and indeed equivocal for specific ethnic groups. However, there is a disconnect between the scale of community-centred intervention provisions and published evidence. This may imply a bias in terms of scientific priorities and funding that needs urgent rectification, as current evidence is limited and weak.

It is important that future research seeks to understand *how* successful interventions work to improve mental health and for whom, taking into account intersectionality such as between gender and ethnicity. This requires resources to be appropriately allocated to third sector organisations, who typically provide these interventions, as they are often bound to deliver short-term projects and lack resources for robust evaluations. Evaluations should also include economic analysis; community interventions are potentially an affordable means to improving public mental health(80).

These findings also suggest the need for more activity on preventing mental illness and promoting wellbeing; most of the studies focussed on people with existing common mental conditions. Health promotion and primary prevention alongside universal approaches are critical components of strong public mental health and sustainable health systems(81). Additionally, further research is needed to understand the societal, structural and institutional challenges affecting community-centred public mental health interventions for BAME groups to help identify potential solutions.

For peer review only - http://bmjopen.bmj.com/site/about/guidelines.xhtml

Lastly, labels such as ‘Asian’ or ‘Black African’ present challenges to mental health research by viewing ethnic minority groups as homogenous in their mental health needs, despite evidence indicating otherwise(82-84). We recommend that national census categories are consistently used across all sectors, so that important variations and inequities can be identified and investigated further.

Conclusion

Despite well-documented ethnic disparities in mental health, there is a paucity of high-quality evidence regarding community-centred interventions that focus on improving public mental health among BAME groups. Decision makers need scientific evidence to help commission appropriate services and to inform effective approaches to mitigating these health disparities. Our next steps are to map the promising community activities and interventions that are currently being provided to help identify emerging evidence.

Data Sharing

Data sharing not applicable as no datasets generated and/or analysed for this study. This was a scoping review with no original individual participant data.

Figure legends

Figure 1. PRISMA flow chart of search strategy

Legend: The PRIMSA diagram details our search and selection process applied during the overview.

For peer review only - http://bmjopen.bmj.com/site/about/guidelines.xhtml

Acknowledgements

Many thanks to the library staff at Imperial College London for their insights in the design of search terms and their support in the use of resources.

Funding Statement

This study is funded by the National Institute for Health Research (NIHR) School for Public Health Research (SPHR) (Grant Reference Number PD-SPH-2015). The views expressed are those of the author(s) and not necessarily those of the NIHR or the Department of Health and Social Care.

The NIHR School for Public Health Research is a partnership between the Universities of Sheffield; Bristol; Cambridge; Imperial; and University College London; The London School for Hygiene and Tropical Medicine (LSHTM); LiLaC - a collaboration between the Universities of Liverpool and Lancaster; and Fuse - The Centre for Translational Research in Public Health a collaboration between Newcastle, Durham, Northumbria, Sunderland and Teesside Universities.

For peer review only

Competing Interests

The authors have no competing interests to declare.

Authors' Contributions

SG conceived the idea. GZ, CB and SG designed and conducted the study, which was supervised by SG. GZ and CB wrote the original draft of the manuscript. MM, CL, FD, EO, DO, JD, EK, LL, KW and JBK contributed to the interpretation of the results and

revised the manuscript critically for intellectual content and technical and language edits. Further reviewing and editing was undertaken by GZ, CB and SG.

References

1. Bhui KS, McKenzie K. Rates and Risk Factors by Ethnic Group for Suicides Within a Year of Contact With Mental Health Services in England and Wales. *Psychiatr Serv*. 2008 Apr;59(4):414–20.doi: 10.1176/ps.2008.59.4.414
2. Weich S, Nazroo J, Sporston K, et al. Common mental disorders and ethnicity in England: The EMPIRIC study. *Psychol Med*. 2004 Nov;34(8):1543–51.doi: 10.1017/s0033291704002715
3. Williams ED, Tillin T, Richards M, et al. Depressive symptoms are doubled in older British South Asian and Black Caribbean people compared with Europeans: Associations with excess co-morbidity and socioeconomic disadvantage. *Psychol Med*. 2015;45(9):1861–71.doi: 10.1017/S0033291714002967
4. Adkison-Bradley C, Maynard D, Johnson P, Carter S. British African Caribbean women and depression. *Br J Guid Couns*. 2009 Feb;37(1):65–72.doi: 10.1080/03069880802535887
5. Ngwena J. Black and minority ethnic groups (BME) suicide, admission with suicide or self-harm: An inner city study. *J Public Heal*. 2014;22(2):155–63.doi: 10.1007/s10389-013-0600-9
6. Karlsen S, Nazroo JY, McKenzie K, et al. Racism, psychosis and common mental disorder among ethnic minority groups in England. *Psychol Med*. 2005 Dec;35(12):1795–803.doi: 10.1017/S0033291705005830
7. Morgan C, Fearon P, Lappin J, et al. Ethnicity and long-term course and outcome of psychotic disorders in a UK sample: The ÆSOP-10 study. *Br J Psychiatry*. 2017 Aug 2;211(2):88–94.doi: 10.1192/bjp.bp.116.193342
8. Bignall T, Jeraj S, Helsby E, et al. Racial disparities in mental health: literature and evidence review — NHS Networks. 2020 [cited 2020 Mar 19]. Available

For peer review only - http://bmjopen.bmj.com/site/about/guidelines.xhtml

from: https://www.networks.nhs.uk/news/racial-disparities-in-mental-health-literature-and-evidence-review

9. Powell A, Francis-Devine B, Foley N. Unemployment by ethnic background. House of Commons Library. 2020.
10. "Overcrowded households," Ethnicity facts and figures, September 9, 2020, service.gov.uk.
11. Memon A, Taylor K, Mohebati LM, et al. Perceived barriers to accessing mental health services among black and minority ethnic (BME) communities: A qualitative study in Southeast England. *BMJ Open*. 2016 Nov 1;6(11):e012337.
12. Uhrig N. Black, Asian and Minority Ethnic disproportionality in the Criminal Justice System in England and Wales. Ministry of Justice Analytical Services. 2016.
13. Barnard H, Turner C. Poverty and ethnicity: A review of evidence. York:JRF.
14. Mantovani N, Pizzolati M, Edge D. Exploring the relationship between stigma and help-seeking for mental illness in African-descended faith communities in the UK. *Heal Expect*. 2017 Jun;20(3):373–84.doi: 10.1111/hex.12464.
15. Abbey S, Charbonneau M, Tranulis C, Moss P, et al. Stigma and discrimination. Can J Psychiatry. 2012;56(10):1–9.
16. Henderson C, Noblett J, Parke H, et al. Mental health-related stigma in healthcare and mental health–care settings. Lancet Psychiatry. 2014;1(6):467–482.
17. Problems amid Progress: Improving Lives and Livlihood for ethnic minorities in the United Kingdom. October 2020, McKinsey.

For peer review only

For peer review only - http://bmjopen.bmj.com/site/about/guidelines.xhtml

18. Definitions | Institute of Race Relations. [cited 2020 May 6]. Available from: http://www.irr.org.uk/research/statistics/definitions/
19. Khunti K, Routen A, Pareek M, Treweek S, Platt L. The language of ethnicity. BMJ. 2020 Nov 23;371.
20. Bignall T, Jeraj S, Helsby E, et al. Racial disparities in mental health: literature and evidence review — NHS Networks. 2020 [cited 2020 Mar 19]. Available from: https://www.networks.nhs.uk/news/racial-disparities-in-mental-health-literature-and-evidence-review
21. Fearon P, Kirkbride JB, Morgan C, et al. Incidence of schizophrenia and other psychoses in ethnic minority groups: Results from the MRC AESOP Study. Psychol Med. 2006 Nov;36(11):1541–50.doi: 10.1017/S0033291706008774.
22. McKenzie K, Bhui K, Nanchahal K, et al. Suicide rates in people of South Asian origin in England and Wales: 1993-2003. *Br J Psychiatry*. 2008 Nov;193(5):406–9.doi: 10.1192/bjp.bp.107.042598.
23. Public mental health - GOV.UK [Internet]. [cited 2020 Mar 19]. Available from: https://www.gov.uk/government/collections/public-mental-health
24. Davies SC. Annual Report of the Chief Medical Officer: Public Mental Health Priorities: Investing in the Evidence. 2014.
25. Department of Health. Delivering race equality in mental health care, an Action Plan for reform inside and outside services; and the Government's response to the indpeendent inquiry into the death of David Bennett. London: 2005.
26. Care Quality Commission. Count me in 2010: Results of the 2010 national census of inpatients and patients on supervised community treatment in mental health and learning disability services in England and Wales. London: 2011.
27. Salway S, Holman D, Lee C, et al. Transforming the health system for the UK's multiethnic population. *BMJ*. 2020; 368:m268
28. Health matters: community-centred approaches for health and wellbeing - GOV.UK [Internet]. [cited 2020 Mar 16]. Available from: https://www.gov.uk/government/publications/health-matters-health-and-

For peer review only - http://bmjopen.bmj.com/site/about/guidelines.xhtml

wellbeing-community-centred-approaches/health-matters-community-centred-approaches-for-health-and-wellbeing

29. NICE. Impact Mental Health. NICE 2019.

30. Local Government Improvement and Development. Culturally responsive JSNAs: a review of race equality and Joint Strategic Needs Assessment (JSNA) practice. 2010.

31. Peters MDJ, Godfrey CM, Khalil H, et al. Guidance for conducting systematic scoping reviews. *Int J Evid Based Healthc*. 2015;13(3):141–6.doi: 10.1097/XEB.0000000000000005

32. Tricco AC, Lillie E, Zarin W, et al. A scoping review on the conduct and reporting of scoping reviews. *BMC Med Res Methodol.* 2016;16(1):1–10.doi: 10.1186/s12874-016-0116-4

33. Arksey H, O'Malley L. Scoping studies: Towards a methodological framework. *Int J Soc Res Methodol Theory Pract*. 2005 Feb;8(1):19–32.doi: 10.1080/1364557032000119616

34. Public mental health programme - NIHR School for Public Health Research NIHR SPHR [Internet]. [cited 2020 May 6]. Available from: https://sphr.nihr.ac.uk/category/research/public-mental-health/

35. Booth A, Clarke M, Dooley G, et al. The nuts and bolts of PROSPERO: An international prospective register of systematic reviews. *Syst Rev.* 2012 Feb 9;1;2.doi: 10.1186/2046-4053-1-2.

36. Duncan, F. H., McGrath, M., Baskin, C., Osborn, D., Dykxhoorn, J., Kaner, E. F., Oliver & Kirkbride, J. (2020). Delivery of community-centred public mental health interventions in diverse areas in England: a mapping study protocol. *BMJ open*, *10*(7), e037631.

37. 50 years of collecting ethnicity data - History of government [Internet]. [cited 2020 Apr 7]. Available from: https://history.blog.gov.uk/2019/03/07/50-years-of-collecting-ethnicity-data/

38. Adult Psychiatric Morbidity Survey: Survey of Mental Health and Wellbeing,

For peer review only

England, 2014. NHS Digital. [cited 2020 Mar 20]. Available from: https://webarchive.nationalarchives.gov.uk/20180328140249/http://digital.nhs.uk/catalogue/PUB21748

39. National Institute for Health and Care Excellence. Common mental health problems: identification and pathways to care. 2011.

40. Porter M, Haslam N. Predisplacement and postdisplacement of refugees and internally displaced persons. *J Am Med Assoc*. 2005;294(5):610–2.doi: 10.1001/jama.294.5.602

41. Steel Z, Chey T, Marnane C, et al. Association of Torture and Other Potentially Traumatic Events With Mental Health Outcomes Among Populations Exposed to Mass Conflict and Displacement: A Systematic Review and Meta-analysis. *J Am Med Assoc*. 2019;302(5).doi: 10.1001/jama.2009.1132.

42. Depression in adults: recognition and management - Clinical Guideline 90. 2009.

43. Generalised anxiety disorder and panic in adults: management - Clinical guideline 113. 2011.

44. National Office for Statistics. 2011 Census Analysis: Ethnicity and Religion of the Non-UK Born Population in England and Wales. 2015;(June):5.

45. Veritas Health Innovation. Covidence systematic review software. Veritas Health Innovation, Melbourne, Australia.

46. Hoffmann TC, Glasziou PP, Boutron I, Milne R, Perera R, Moher D, et al. Better reporting of interventions: Template for intervention description and replication (TIDieR) checklist and guide. *BMJ*. 2014 Mar;348:g1687

47. Higgins JP, Altman DG. Assessing Risk of Bias in Included Studies. In: Cochrane Handbook for Systematic Reviews of Interventions: Cochrane Book Series. 2008.

48. Noyes J, Booth A, Flemming K, et al. Cochrane Qualitative and Implementation Methods Group guidance series—paper 3: methods for assessing methodological limitations, data extraction and synthesis, and confidence in synthesized qualitative findings. *J Clin Epidemiol*. 2018 May;97:49–58.doi: 10.1016/j.jclinepi.2017.06.020. Epub 2017 Dec 13.

49. Pluye P, Gagnon MP, Griffiths F, et al. A scoring system for appraising mixed methods research, and concomitantly appraising qualitative, quantitative and

For peer review only - http://bmjopen.bmj.com/site/about/guidelines.xhtml

mixed methods primary studies in Mixed Studies Reviews. Int J Nurs Stud. 2009 Apr; 46 (4):529-46.doi: 10.1016/j.ijnurstu.2009.01.009.Epub 2009 Feb 23.

50. Tricco AC, Lillie E, Zarin W, et al. PRISMA extension for scoping reviews (PRISMA-ScR): Checklist and explanation. Vol. 169, Annals of Internal Medicine. American College of Physicians; 2018. p. 467–73.

51. Afuwape SA, Craig TKJ, Harris T, et al. The Cares of Life Project (CoLP): An exploratory randomised controlled trial of a community-based intervention for black people with common mental disorder. *J Affect Disord*. 2010;127(1–3):370–4.doi: 10.1016/j.jad.2010.05.017. Epub 2010 Jun 14.

52. Lovell K, Lamb J, Gask L, et al. Development and evaluation of culturally sensitive psychosocial interventions for under-served people in primary care. *BMC Psychiatry*. 2014 Aug 1;14:217.doi: 10.1186/s12888-014-0217-8.

53. Gater R, Waheed W, Husain N, Tomenson B, Aseem S, Creed F. Social intervention for British Pakistani women with depression: Randomised controlled trial. *Br J Psychiatry.* 2010;197(3):227–33.doi: 10.1192/bjp.bp.109.066845.

54. Chaudhry N, Waheed W, Husain N, et al. Development and pilot testing of a social intervention for depressed women of Pakistani family origin in the UK. *J Ment Heal.* 2009;18(6):504–9.doi: 10.3109/09638230902968209.

55. Jacob KS, Bhugra D, Mann AH. A Randomised Controlled Trial of an Educational Intervention for Depression Among Asian Women in Primary Care in the United Kingdom. *Int J Soc Psychiatry*. 2002 Jun;48(2):139-48.doi: 10.1177/002076402128783190.

56. Rabiee F, Robbins A, Khan M. Gym for Free: The short-term impact of an innovative public health policy on the health and wellbeing of residents in a deprived constituency in Birmingham, UK. *Health Educ J.* 2015;74(6):691–704.

57. Gray B. Social Exclusion, Poverty, Health and Social Care in Tower Hamlets: The Perspectives of Families on the Impact of the Family Support Service. *Br J Soc Work*. 2003;33(3):361–80.doi: 10.1093 bjsw33.3.361

58. Krueger R, Casey M. Focus groups: A practical guide for applied research. Thousand Oaks, CA: Sage Publications 2000.

59. Rabiee F. Focus-group interview and data analysis. *The Proceedings of the*

For peer review only

Nutrition Society. 2004, 63(4):655-660.doi: 10.1079/pns2004399

60. Garfinkel E, Cliffs H. Studies in ethnomethodology.1967.
61. Glaser BG, Strauss AL. Discovery of grounded theory: Strategies for qualitative research. Discovery of Grounded Theory: Strategies for Qualitative Research. Taylor and Francis; 1967. 1–271 p.
62. Tabassum R, Macaskill A, Ahmad I. Attitudes towards mental health in an urban Pakistani community in the United Kingdom. *Int J Soc Psychiatry*. 2000;46(3):170–81.doi: 10.1177/002076400004600303.
63. Sheikh S, Furnham A. A cross-cultural study of mental health beliefs and attitudes towards seeking professional help. *Soc Psychiatry Psychiatr Epidemiol*. 2000;35(7):326–34.doi: 10.1007/s001270050246.
64. Chowdhary N, Jotheeswaran AT, Nadkarni A, et al. The methods and outcomes of cultural adaptations of psychological treatments for depressive disorders: a systematic review. *Psychol Med.* 2014 Apr;44(6):1131-46.doi: 10.1017/S0033291713001785. Epub 2013 Jul 19.
65. Benish S, Quintana S, Wampold B. Culturally Adapted Psychotherapy and the Legitimacy of Myth: A Direct-Comparison Meta-Analysis. *J Couns Psychol*. 2011 Jul;58(3):279-89.doi: 10.1037/a0023626.
66. Shahmalak U, Blakemore A, Waheed MW, et al. The experiences of lay health workers trained in task-shifting psychological interventions: a qualitative systematic review. *Int J Ment Health Syst*. 2019;13:64.doi: 10.1186/s13033-019-0320-9.
67. Tabassum R, Macaskill A. Attitudes towards mental health in an urban Pakistani community in the United Kingdom. *Int J Soc Psychiatry*. 2000;46(3):170–81.doi: 10.1177/002076400004600303.
68. Li PL, Logan S, Yee L, et al. Barriers to meeting the mental health needs of the Chinese community. *J Public Health Med*. 1999 Mar;21(1):74–80.doi: 10.1093/pubmed/21.1.74.

For peer review only - http://bmjopen.bmj.com/site/about/guidelines.xhtml

69. Singla D, Lazarus A, Atif N, et al. "Someone like us": Delivering maternal mental health through peers in two South Asian contexts. *J Affect Disord.*2014 Oct;168:452-8.doi: 10.1016/j.jad.2014.07.017.

70. McClay CA, Morrison J, McConnachie A, et al. A community-based group-guided self-help intervention for low mood and stress: Study protocol for a randomized controlled trial. *Trials.* 2013; 14:392.doi: 10.1186/1745-6215-14-392.

71. Mueller M, Kennerley H, McManus F et al. Oxford Guide to Surviving as a CBT Therapist. Oxford University Press 2010.

72. Iheanacho, T., Kapadia, D., Ezeanolue, C.O., Osuji, A.A., Ogidi, A.G., Ike, A., Patel, D., Stefanovics, E., Rosenheck, R., Obiefune, M. and Ezeanolue, E.E., 2016. Attitudes and beliefs about mental illness among church-based lay health workers: experience from a prevention of mother-to-child HIV transmission trial in Nigeria. *International Journal of Culture and Mental Health*, *9*(1), pp.1-13

73. Bickerdike L, Booth A, Wilson PM, et al. Social prescribing: Less rhetoric and more reality. A systematic review of the evidence. *BMJ Open*. 2017 Apr;7(4).doi:10.1136/bmjopen-2016-013384

74. Oliver MI, Pearson N, Coe N, et al. Help-seeking behaviour in men and women with common mental health problems: Cross-sectional study. *Br J Psychiatry*. 2005 Apr;186:297-301.doi: 10.1192/bjp.186.4.297.

75. Mcdaid D, Park A-L, Wahlbeck K. The Economic Case for the Prevention of Mental Illness. *Annu Rev Public Heal*. 2019 Apr 1;40:373–89.doi: 10.1146/annurev-publhealth-040617-013629. Epub 2019 Jan 2

76. Global Trends: Forced Displacement in 2019, 2019. UNHCR. [cited 2020 Dec

For peer review only

17] Available from: https://www.unhcr.org/5ee200e37.pdf

77. Migration Statistics Quarterly Report: August 2020, 2020. ONS. [cited 2020 Dec 17] Available from: https://www.ons.gov.uk/peoplepopulationandcommunity/populationandmigration/internationalmigration/bulletins/migrationstatisticsquarterlyreport/august2020

78. Byrow Y, Pajak R, McMahon T, et al. Barriers to mental health help-seeking amongst refugee men. *Int J Environ Res Public Health*. 2019 Aug 1;16(15):2634.doi: 10.3390/ijerph16152634.

79. Hynie M. The Social Determinants of Refugee Mental Health in the Post-Migration Context: A Critical Review. *Can J Psychiatry.* 2018 May;63(5):297-303.doi: 10.1177/0706743717746666.

80. McLaughlin KA. The Public Health Impact of Major Depression: A Call for Interdisciplinary Prevention Efforts, *Prevention Science*. 2011 Nov; 12(4): 361-371. doi: 10.1007/s11121-011-0231-8

81. Mcdaid D, Park A-L, Wahlbeck K. The Economic Case for the Prevention of Mental Illness. *Annu Rev Public Heal*. 2019 Apr 1;40:373–89.doi: 10.1146/annurev-publhealth-040617-013629. Epub 2019 Jan 2.

82. Care Quality Commission. Count me in: 2010 Results of the 2010 national census of inpatients and patients on supervised community treatment in mental health and learning disability services in England and Wales. London, UK: Care Quality Commission; 2011.

83. Care Quality Commission. Count Me In: Results of the 2009 National Census of Inpatients and Patients on Supervised Community Treatment Orders in Mental Health and Learning. London, UK: Care Qiuality Commission; 2010.

For peer review only - http://bmjopen.bmj.com/site/about/guidelines.xhtml

84. Weich S, Nazroo J, Sprostoin K, et al. Common mental disorders and ethnicity in England: the EMPIRIC Study. *Psychol Med.* 2004; 34(8):1543-1551.doi:10.1017/s0033291704002715

For peer review only

For peer review only - http://bmjopen.bmj.com/site/about/guidelines.xhtml

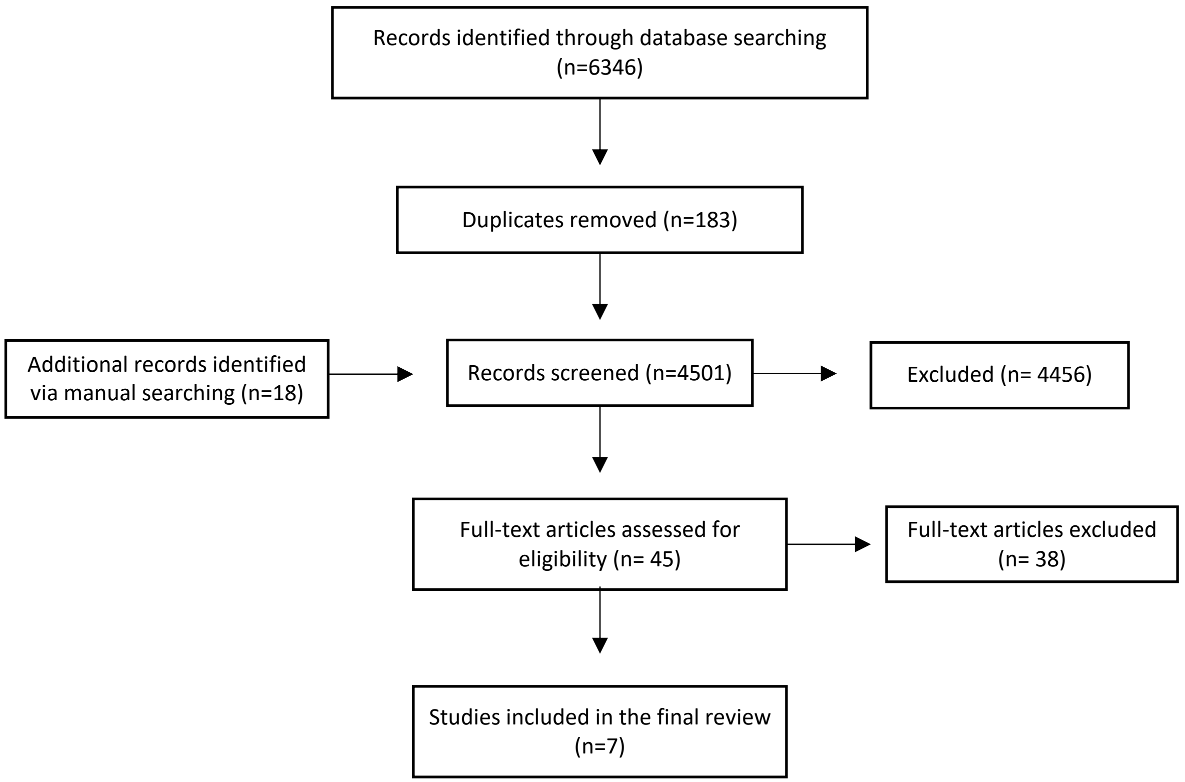
Records identified through database searching (n=6346)
Duplicates removed (n=183)
Additional records identified via manual searching (n=18)
Records screened (n=4501)
Excluded (n= 4456)
Full-text articles assessed for eligibility (n= 45)
Full-text articles excluded (n= 38)
Studies included in the final review (n=7)

Appendix 1. Search strategy in all databases

Database Searched	Ethnicity Terms	Outcome Terms (Mental Health)	Geography Terms	Population terms
**Medline**	minority groups.mp. OR Minority Groups/ OR ethnic groups.mp. OR exp Ethnic Groups/ OR ethnic*.mp. OR Race.mp. OR exp Continental Population Groups/ OR racial.mp. OR<br><br>Black.mp. OR exp African Continental Ancestry Group/ OR Black African*.mp OR British African*.mp OR (Black adj1 British).mp. OR Afro$Caribbean*.mp OR Black adj1 caribbean.mp OR (Afr* adj3 Caribbean) OR Black*.mp. OR<br><br>Asian.mp. OR exp Asian Continental Ancestry Group/ OR Indian*.mp. OR exp Pakistan/ OR Pakistan*.mp. OR Bangladesh*.mp. OR exp Bangladesh/ OR Bengali*.mp/ OR Chinese.mp. OR<br><br>Arab*.mp. OR exp Arabs OR	Mental Health.mp. OR exp Mental Health/ OR mental well$being.mp OR exp Stress, Psychological/ OR psychological well$being.mp. OR Resilience, Psychological/ OR resilien*.mp. OR exp Occupational Stress OR stress.mp.	exp United Kingdom/ OR UK.mp OR United Kingdom.mp. OR Great Britain.mp OR Britain.mp OR GB.mp OR England.mp OR Scotland.mp OR Wales.mp OR North* Ireland.mp OR British.mp OR Scottish.mp OR Welsh.mp OR North* Irish.mp	Adult*<br>Wom?n<br>Men<br>people

	Gypsy or "Irish traveller" OR<br><br>(white and black African*).mp. OR (white and black caribbean).mp. OR (white and Asian).mp. OR mixed ethnicit*.mp			
**Embase**	minority groups.mp. OR exp Minority Groups/ OR ethnic groups.mp. OR exp Ethnic Group/ OR ethnic*.mp. OR exp race/ OR "ethnic or racial aspects"/ OR Race.mp. OR racial.mp. OR<br><br>Black*.mp. OR exp Black person/ OR black african*.mp OR British African*.mp OR (Black adj1 British).mp. OR Afro$Caribbean*.mp OR (Black adj1 caribbean).mp OR (Afr* adj3 Caribbean).mp. OR<br><br>Asian.mp. OR exp Asian/ OR exp Asian continental ancestry group/ OR Indian*.mp. OR exp Indian/ OR Pakistan*.mp. OR exp Pakistan/ OR exp Bangladesh/ OR Bangladesh*.mp. OR Bengali*.mp OR exp "Bengali (people)"/ OR Chinese.mp. OR exp Chinese/ OR	Mental Health.mp. OR exp Mental Health/ OR [mental well$being.mp] OR exp psychological well-being/ OR psychological stress.mp. OR exp mental Stress/OR psychGological well$being.mp. OR exp psychological resilience/ OR resilien*.mp OR job stress/ OR life stress/ OR chronic stress/ OR emotional stress/ OR social stress/ OR behavioural stress/ OR stress.mp.	Exp united Kingdom/ OR United Kingdom.mp. OR UK.mp. OR Great Britain.mp OR Britain.mp OR GB.mp OR England.mp OR Scotland.mp OR Wales.mp OR North* Ireland.mp OR British.mp OR Scottish.mp OR Welsh.mp OR North* Irish.mp	Adult*<br>Wom?n<br>Men<br>people

For peer review only - http://bmjopen.bmj.com/site/about/guidelines.xhtml

	Arab*.mp. OR exp Arab/ OR<br><br>Gypsy or "Irish traveller" OR<br><br>(white and black African*).mp. OR (white and black Caribbean*).mp. OR (white and Asian*).mp.] OR mixed ethnic*.mp			
**PsychINFO**	minority groups.mp. OR exp Minority Group/ OR ethnic groups.mp. OR exp 'Racial and Ethnic groups'/ OR ethnic*.mp. OR Race.mp. OR racial.mp. OR 'race and ethnic discrimination'/ OR<br><br>exp Blacks/ OR black*.mp. OR exp African Cultural Groups/ or black African*.mp. OR British African*.mp OR (Black adj1 British).mp. OR Afro$Caribbean*.mp OR (Black adj1 caribbean).mp OR (afr* adj3 Caribbean).mp. OR Asian.mp. OR exp Asians/ OR Indian*.mp. OR Pakistan*.mp. OR Bangladesh*.mp. OR Bengali*.mp OR Chinese.mp. OR<br><br>Arab*.mp. OR<br><br>Gypsy or "Irish traveller" OR	Mental Health.mp. OR exp Mental Health/ OR mental well$being.mp OR exp Well Being/ OR exp Psychological Stress/or psychological well$being.mp. OR exp "Resilience (Psychological)"/OR resilien*.mp. OR exp Occupational Stress/ OR stress.mp. OR exp Chronic stress/ OR exp Social Stress/	United Kingdom.mp. OR UK.mp. OR Great Britain.mp OR Britain.mp OR GB.mp OR England.mp OR Scotland.mp OR Wales.mp OR North* Ireland.mp OR British.mp OR Scottish.mp OR Welsh.mp OR North* Irish.mp	Adult*<br>Wom?n<br>Men<br>people

For peer review only - http://bmjopen.bmj.com/site/about/guidelines.xhtml

	(white and black African*).mp. OR (white and black Caribbean*).mp. OR (white and Asian*).mp. OR<br><br>mixed ethnicit*.mp			
**SCOPUS**	("minority group*" OR ethnic* OR race OR racial OR african* OR (black W/1 british) OR afro$caribbean* OR (black W/1 caribbean) OR indian OR pakistani OR arab* OR asian OR indian OR bangladesh* OR bengali OR chinese OR "mixed ethnic*" OR "mixed race")	("mental health" OR "mental well?being" OR "psychological well?being" OR "psychological stress" OR "psychological resilien*")	"united kingdom" OR "Great Britain" OR England OR Scotland OR Wales OR (North* Ireland) OR British OR Scottish OR Welsh OR (North* Irish)	Adult* Wom?n Men<br><br>people
**CINAHL**	(minority group*) OR (ethnic* group*) OR ethnic* OR race OR racial OR "black African" OR "African Cultural Groups" OR (british W/1 african) OR (black W/1 british) OR Afro$ Caribbean* OR (black W/1 caribbean) OR (afr* W/3 caribbean) OR asian OR indian OR pakistan* OR bangladesh* OR bengali OR chinese OR arab* OR	(Mental Health) OR (mental well?being) OR (Well?Being) OR (Psychological Stress) OR (psychological well?being) OR exp (psychological resilien*) OR (Chronic stress) OR (Social Stress)	"united kingdom" OR "Great Britain" OR England OR Scotland OR Wales OR (North* Ireland) OR British OR Scottish OR Welsh OR (North* Irish)	Adult* Wom?n Men<br><br>people

For peer review only - http://bmjopen.bmj.com/site/about/guidelines.xhtml

	"mixed ethnicit*" or Gypsy or "Irish traveller"			
**Cochrane**	"minority group*" OR ethnic* OR race OR racial OR african* OR (black near/1 british) OR afro$caribbean* OR (black near/1 caribbean) OR asian OR indian OR pakistan* OR bangladesh* OR bengali* OR chinese OR arab* OR "mixed ethnicit*" OR gypsy OR "Irish traveller"	(Mental Health) OR (mental well?being) OR (Well?Being) OR (Psychological Stress) OR (psychological well?being) OR exp (psychological resilien*) OR (Chronic stress) OR (Social Stress)	"united kingdom" OR "Great Britain" OR England OR Scotland OR Wales OR (North* Ireland) OR British OR Scottish OR Welsh OR (North* Irish)	Adult* Wom?n Men<br><br>people

For peer review only - http://bmjopen.bmj.com/site/about/guidelines.xhtml

Preferred Reporting Items for Systematic reviews and Meta-Analyses extension for Scoping Reviews (PRISMA-ScR) Checklist

SECTION	ITEM	PRISMA-ScR CHECKLIST ITEM	REPORTED ON PAGE #
**TITLE**			
Title	1	Identify the report as a scoping review.	1
**ABSTRACT**			
Structured summary	2	Provide a structured summary that includes (as applicable): background, objectives, eligibility criteria, sources of evidence, charting methods, results, and conclusions that relate to the review questions and objectives.	2
**INTRODUCTION**			
Rationale	3	Describe the rationale for the review in the context of what is already known. Explain why the review questions/objectives lend themselves to a scoping review approach.	5,6
Objectives	4	Provide an explicit statement of the questions and objectives being addressed with reference to their key elements (e.g., population or participants, concepts, and context) or other relevant key elements used to conceptualize the review questions and/or objectives.	5
**METHODS**			
Protocol and registration	5	Indicate whether a review protocol exists; state if and where it can be accessed (e.g., a Web address); and if available, provide registration information, including the registration number.	6
Eligibility criteria	6	Specify characteristics of the sources of evidence used as eligibility criteria (e.g., years considered, language, and publication status), and provide a rationale.	7,8
Information sources*	7	Describe all information sources in the search (e.g., databases with dates of coverage and contact with authors to identify additional sources), as well as the date the most recent search was executed.	6
Search	8	Present the full electronic search strategy for at least 1 database, including any limits used, such that it could be repeated.	appendix
Selection of sources of evidence†	9	State the process for selecting sources of evidence (i.e., screening and eligibility) included in the scoping review.	9
Data charting process‡	10	Describe the methods of charting data from the included sources of evidence (e.g., calibrated forms or forms that have been tested by the team before their use, and whether data charting was done independently or in duplicate) and any processes for obtaining and confirming data from investigators.	10
Data items	11	List and define all variables for which data were sought and any assumptions and simplifications made.	10
Critical appraisal of individual sources of evidence§	12	If done, provide a rationale for conducting a critical appraisal of included sources of evidence; describe the methods used and how this information was used in any data synthesis (if appropriate).	10,11

For peer review only - http://bmjopen.bmj.com/site/about/guidelines.xhtml

SECTION	ITEM	PRISMA-ScR CHECKLIST ITEM	REPORTED ON PAGE #
Synthesis of results	13	Describe the methods of handling and summarizing the data that were charted.	10,11
**RESULTS**			
Selection of sources of evidence	14	Give numbers of sources of evidence screened, assessed for eligibility, and included in the review, with reasons for exclusions at each stage, ideally using a flow diagram.	10
Characteristics of sources of evidence	15	For each source of evidence, present characteristics for which data were charted and provide the citations.	14
Critical appraisal within sources of evidence	16	If done, present data on critical appraisal of included sources of evidence (see item 12).	17-22
Results of individual sources of evidence	17	For each included source of evidence, present the relevant data that were charted that relate to the review questions and objectives.	17-22
Synthesis of results	18	Summarize and/or present the charting results as they relate to the review questions and objectives.	14,15
**DISCUSSION**			
Summary of evidence	19	Summarize the main results (including an overview of concepts, themes, and types of evidence available), link to the review questions and objectives, and consider the relevance to key groups.	23
Limitations	20	Discuss the limitations of the scoping review process.	24,25
Conclusions	21	Provide a general interpretation of the results with respect to the review questions and objectives, as well as potential implications and/or next steps.	26,27
**FUNDING**			
Funding	22	Describe sources of funding for the included sources of evidence, as well as sources of funding for the scoping review. Describe the role of the funders of the scoping review.	27

JBI = Joanna Briggs Institute; PRISMA-ScR = Preferred Reporting Items for Systematic reviews and Meta-Analyses extension for Scoping Reviews.
* Where *sources of evidence* (see second footnote) are compiled from, such as bibliographic databases, social media platforms, and Web sites.
† A more inclusive/heterogeneous term used to account for the different types of evidence or data sources (e.g., quantitative and/or qualitative research, expert opinion, and policy documents) that may be eligible in a scoping review as opposed to only studies. This is not to be confused with *information sources* (see first footnote).
‡ The frameworks by Arksey and O'Malley (6) and Levac and colleagues (7) and the JBI guidance (4, 5) refer to the process of data extraction in a scoping review as data charting.
§ The process of systematically examining research evidence to assess its validity, results, and relevance before using it to inform a decision. This term is used for items 12 and 19 instead of "risk of bias" (which is more applicable to systematic reviews of interventions) to include and acknowledge the various sources of evidence that may be used in a scoping review (e.g., quantitative and/or qualitative research, expert opinion, and policy document).

From: Tricco AC, Lillie E, Zarin W, O'Brien KK, Colquhoun H, Levac D, et al. PRISMA Extension for Scoping Reviews (PRISMAScR): Checklist and Explanation. Ann Intern Med. 2018;169:467–473. doi: 10.7326/M18-0850.

For peer review only - http://bmjopen.bmj.com/site/about/guidelines.xhtml
